# The Cultured Microbiome of Pollinated Maize Silks Shifts after Infection with *Fusarium graminearum* and Varies by Distance from the Site of Pathogen Inoculation

**DOI:** 10.3390/pathogens12111322

**Published:** 2023-11-06

**Authors:** Michelle E. H. Thompson, Anuja Shrestha, Jeffrey Rinne, Victor Limay-Rios, Lana Reid, Manish N. Raizada

**Affiliations:** 1 Department of Plant Agriculture, University of Guelph, Guelph, ON N1G 2W1, Canada; michelleehthompson@gmail.com (M.E.H.T.);; 2 Department of Plant Agriculture, University of Guelph Ridgetown Campus, 120 Main Street E, Ridgetown, ON N0P 2C0, Canada; 3 Ottawa Research and Development Centre, Agriculture and Agri-Food Canada, Central Experimental Farm, 960 Carling Avenue, Ottawa, ON K1A 0C6, Canada

**Keywords:** maize, microbiome, cultured, silk, style, *Fusarium graminearum*, pollen tube, heterotic group, North America, reproduction

## Abstract

Styles transmit pollen-derived sperm nuclei from pollen to ovules, but also transmit environmental pathogens. The microbiomes of styles are likely important for reproduction/disease, yet few studies exist. Whether style microbiome compositions are spatially responsive to pathogens is unknown. The maize pathogen *Fusarium graminearum* enters developing grain through the style (silk). We hypothesized that *F. graminearum* treatment shifts the cultured transmitting silk microbiome (TSM) compared to healthy silks in a distance-dependent manner. Another objective of the study was to culture microbes for future application. Bacteria were cultured from husk-covered silks of 14 *F. graminearum*-treated diverse maize genotypes, proximal (tip) and distal (base) to the *F. graminearum* inoculation site. Long-read 16S sequences from 398 isolates spanned 35 genera, 71 species, and 238 OTUs. More bacteria were cultured from *F. graminearum*-inoculated tips (271 isolates) versus base (127 isolates); healthy silks were balanced. *F. graminearum* caused a collapse in diversity of ~20–25% across multiple taxonomic levels. Some species were cultured exclusively or, more often, from *F. graminearum*-treated silks (e.g., *Delftia acidovorans*, *Klebsiella aerogenes*, *K. grimontii*, *Pantoea ananatis*, *Stenotrophomonas pavanii*). Overall, the results suggest that *F. graminearum* alters the TSM in a distance-dependent manner. Many isolates matched taxa that were previously identified using V4-MiSeq (core and *F. graminearum*-induced), but long-read sequencing clarified the taxonomy and uncovered greater diversity than was initially predicted (e.g., within *Pantoea*). These isolates represent the first comprehensive cultured collection from pathogen-treated maize silks to facilitate biocontrol efforts and microbial marker-assisted breeding.

## 1. Introduction

The style channel transmits pollen-derived sperm nuclei inside a growing pollen tube to an ovule but also acts as a conduit for environmental pathogens [1,2]. In maize, silks are a susceptible entry point for many pathogens, including fungi which produce mycotoxins, some of which are carcinogenic [3]. Silk-invading, mycotoxigenic fungi are a global problem, and silks are thus an important route to defend. Maize is frequently infected by *Fusarium graminearum*, which causes Gibberella Ear Rot (GER) and Gibberella stalk rot [4], and produces mycotoxins including trichothecene deoxynivalenol (DON, 3,7,15-trihydroxy-12,13-epoxytrichothec-9-ene-8-one, or vomitoxin), as well as zearalenone in the grain before and after harvest [5,6,7]. *F. graminearum* conidia germinate on silk surfaces, and hyphae subsequently grow through the silks, reaching the developing grain [8]. This process can take 7–15 days [8]. The fungus can also exit an individual silk and access other parts of the cob [8]. Ears are more susceptible after silk emergence, but once silks have dried, *F. graminearum* can no longer travel through them [9,10]. The most susceptible period for *F. graminearum* infection of maize silks is after pollination, as silks begin to senesce [3,11]. Thus, silks in the pollen transmission stage are susceptible to *F. graminearum*. *F. graminearum* is a global pathogen and is of importance to Ontario and Canada [12,13,14,15], where the current study was conducted. This mycotoxigenic fungus negatively impacts human and animal health, and has consequent economic impacts [5,7,16]. There has also been an increase in popularity of no-till agriculture [17], which perpetuates the life cycle of *F. graminearum* [18]. Specifically, this fungal pathogen overwinters in plant debris, and no-till agriculture allows infected crop residue to persist in the soil, causing transmission of *F. graminearum* to the next growing season [18].

Maize has an endogenous defense system in its silks, including the production of maysin which suppresses corn earworm larvae (*Heliothis zea* (Boddie)) [19,20,21]. In response to *F. graminearum* infection, plants also produce phenolic compounds which likely contribute to host defense [22]. Resistance can be categorized as “silk resistance” and “kernel resistance” [23]. Due to significant breeding efforts, some genotypes are moderately resistant to GER, due to having lower susceptibility to infection or slower infection rates [24,25]. Maize lines that slow the infection process may give the kernels more time to develop, thus rendering the plant moderately resistant [8]. Lines such as the inbreds CO272, CO433 and CO441, and hybrid Pride K127, have shown moderate resistance to *F. graminearum* [4,8,23,26,27]. However, especially in years with conducive weather, *F. graminearum* still poses a significant threat despite long-term breeding efforts [28,29]. Resistant maize lines are still notably susceptible to *F. graminearum* [11,29]. Chemical approaches such as fungicide treatments increase inputs and the cost to growers. *F. graminearum* could potentially develop resistance to fungicides, which would increase the need for new approaches to combat this pathogen [30]. A natural strain of *F. graminearum* was reported to be resistant to tebuconazole in 2014 [31], and fungicide resistance is a current area of concern for *F. graminearum* in North America [32]. Additionally, fungicides used to kill *F. graminearum* could allow other pathogens to flourish [33]. Overall, the current strategies to suppress GER are inadequate, especially during severe outbreaks.

Plants are known to possess microbiomes consisting of bacteria and fungi that live as shoot-surface epiphytes, sub-surface endophytes, and root-surface microbes associated with soil [34,35,36,37]. Endophytes are organisms that live within plants without causing disease symptoms [34,35,37]. Limited evidence suggests that some endophytic microbes can be inherited through seeds [34,37]. Endophytes spend part of their life cycle within the plant and receive benefits from the plant such as shelter and nutrients [34,35,37]. Aside from causing no apparent harm, an endophyte may provide benefits to the plant as well, such as promoting plant growth and health [34,35,37].

It has been recognized in the literature that flowers of dioecious species possess microbial diversity and composition that are different in male and female flowers [38,39,40]. The microbiomes of individual flower structures are intriguing [1,2]. The stigma of apple, susceptible to the fire blight pathogen *Erwinia amylovora*, was found to have a dynamic microbiome that shifted after pathogen inoculation [41].

Few studies exist on the microbiome of the style, despite its importance for plant reproduction and disease. Using standard short-read 16S Illumina sequencing (V4-MiSeq), it was recently reported that transmitting silks of maize, the silks stage with the greatest susceptibility to *F. graminearum*, have a microbiome, which the authors termed, “the transmitting silk microbiome (TSM)” [42]. The TSM of the silk tip tissue displayed seasonal responsiveness, but possessed a reproducible core of 7–11 MiSeq-amplicon sequence variants (ASVs) dominated by a single *Pantoea* MiSeq-taxon (15–26% of sequence-counts). Khalaf et al. [42] observed an increase in total read counts and a collapse in diversity associated with *F. graminearum* treatment. *F. graminearum*-infection elevated 7–25 V4-MiSeq-ASVs; these taxa were hypothesized to be anti-fungal, or otherwise associated with *F. graminearum*. The MiSeq sequencing was limited to 245 bp reads, and different kits were used for DNA isolation from tip and base tissue samples, preventing spatial comparisons, as well as close comparisons amongst genotypes and heterotic groups. Nevertheless, the study by Khalaf et al. [42] generated novel hypotheses and suggested bacteria from transmitting silks may represent a novel source of biocontrol agents to combat GER in maize.

Beneficial microbes can interact with pathogens directly or indirectly by inducing host resistance [43,44,45]. A recent study reported culturing of bacteria from maize silks in Brazil and found promising candidates for biocontrol of *Fusarium verticillioides* stalk rot and stored grains [46,47]. This study sampled environmentally exposed silk tissue from cobs randomly collected from fields. The Brazilian study, combined with the study by Khalaf et al. [42], showed potential for anti-*F. graminearum* microbes to be found in maize silks. 

Although others have suggested that in dioecious species the inheritance of microbes is only relevant for female flowers and fruits [38], it is reasonable to assume that pollen, via the pollen tube growing inside transmitting silks, may contribute to the TSM. The pollen microbiome is also a relatively novel area of active research [48,49,50,51]. To the best of our knowledge, there are no prior studies of biocontrol candidates isolated from maize pollen or pollinated silks.

Most of the previous efforts at biocontrol of *F. graminearum* have used microbes isolated from non-silk and non-pollen sources [52,53]. For example, bacterial endophytes including *Paenibacillus* and *Citrobacter* sp. isolated from maize and teosinte roots, shoots and seeds were shown to reduce GER when sprayed onto silks [54]. A potent *Enterobacter* strain (M6) isolated from finger millet roots was similarly shown to suppress GER when sprayed onto maize silks [45,55]. Despite these efforts, to the best of our knowledge, no successful anti-*F. graminearum* biocontrol agent has been widely adopted commercially due to limited efficacy under field conditions. One reason for the lack of prior success may be that the biocontrol agents did not originate from, and hence were not adapted to, the tissue and developmental stage relevant to the pathogenic phase of the *F. graminearum* life cycle—namely, transmitting silks. Furthermore, the current literature points to microbial consortia, rather than individual strains, being integral to a healthy plant microbiome [56,57] and, more specifically, to control *Fusarium* pathogens [58,59,60,61,62]. Combined, these observations indicate that it may be important to evaluate transmitting silk microbiomes as a whole and employ complex consortia.

It has been proposed that anti-*F. graminearum* microbes may be applied at the time of plant reproduction and be integrated into the vertically transmitted microbiome [34,43]. Furthermore, the microbiome can be used in breeding efforts [63,64], particularly with a focus on host plant genes which encourage anti-*F. graminearum* members of the microbiome [43]. More generally, cereal crop breeding is known to impact the microbiome in the roots and rhizosphere [43,65,66]. 

Spatial evaluation of the microbiomes of individual plant organs is limited but is of emerging interest; for example, attention has been paid to colonization patterns across the surface of leaves [67], along the length of roots [68], or in non-plant species such as across the surface of kelp [69]. Given its uniformity, particularly regarding the portion enclosed within protective husk leaves, the silk represents a novel model system for studying a microbiome spatially, including the impact of the distance from a pathogen. The silk tip is proximal to environmental microbes including pathogens, whereas the silk base is more insulated.

The cultured TSM of non-infected (healthy) transmitting maize silks is being reported in a parallel study by Thompson et al. [70] Here, we hypothesized that (1) *F. graminearum* treatment causes a shift in the cultured microbiome of transmitting silks compared to healthy silks and that (2) distance from *F. graminearum* application affects the cultured microbiome of transmitting silks. Our additional objective was to culture isolates that taxonomically matched *F. graminearum*-induced taxa previously defined by V4-MiSeq to obtain higher taxonomic resolution and to contribute to future studies and applications (e.g., biocontrol).

## 2. Materials and Methods

### 2.1. Overview of Methods

Bacteria were cultured from non-infected open pollinated silks of 14 genotypes of maize grown outdoors at the Ridgetown Campus, University of Guelph in Ridgetown, Ontario, Canada, in 2017 (Table 1). The field trial was a randomized split block design, with non-infected plots adjacent to those intentionally infected with *F. graminearum* and divided by guard rows (see below). Here, we report the bacteria that were cultured from the silks infected with *F. graminearum* and compare the cultured microbiome to the results for healthy, uninfected silks that are being presented in a parallel study by Thompson et al. [70]. The cultured bacteria underwent a research pipeline (Supplementary Text S1) that encompassed the initial culturing, purification, full-length 16S rDNA sequencing, contig assembly and BLAST searching to enable taxonomic predictions and OTU assignments, followed by phylogenetic tree construction and comparisons to V4-MiSeq results (see below). 

For one isolate, further investigation was needed to determine its identity, specifically the isolate identified as *Obesumbacterium proteus*. This species was historically split into two biogroups, of which Biogroup 2 was reclassified to *Shimwellia pseudoproteus* gen. nov., sp. nov. [71]. However, the *O. proteus* isolated from silks had a 16S sequence similar to *Serratia* and *Hafnia*, which indicates that it likely belongs to Biogroup 1, which is a true *O. proteus.*

### 2.2. Comparison to V4-MiSeq Results

The 16S sequences were compared to the shorter-read V4-MiSeq results of Khalaf et al. [42], which analyzed both non-infected silks [70] and *Fusarium*-infected silks across two trial years (2016 and 2017) with 4 blocks. The bacteria here were cultured from split, crushed silk tissue samples from one block of the 2017 trial; half of each sample was used earlier for the V4-MiSeq study and the remainder was used here for culturing. The V4-MiSeq core OTUs from Khalaf et al. [42] represented the most prevalent taxa (appearing in at least 50% of samples) and dominant taxa (relative abundance of ≥1% in at least some samples) and were also shared across tip and base tissues (in at least one year/treatment). The V4-MiSeq OTUs which significantly increased in relative abundance following *F. graminearum* treatment (induced by *F. graminearum*) and were also prevalent across samples were labeled *F. graminearum*-indicators. Some taxa were members of both the V4-MiSeq core and *F. graminearum*-indicator groups. These OTUs were BLAST-searched within the 16S sequences in the current study.

The thresholds for matches between isolate sequences and V4-MiSeq-amplicon sequence variants required a ≥96.06% identity with only N mismatches allowed (or a single gap directly adjacent to multiple N’s). When an isolate matched multiple V4-MiSeq OTUs, the match with the higher percent identity was kept. The terms “cultured OTUs” (generated from longer-length sequences cultured bacteria in the current study) and “V4-MiSeq OTUs” (previously generated from shorter-length V4-MiSeq data in Khalaf et al. [42]) were used to differentiate the two types of OTUs.

### 2.3. Fusarium graminearum Field Treatments

For *F. graminearum* treatment details, readers can refer to the MiSeq study by Khalaf et al. [42] because the silk samples used for culturing in this study were a subset of the samples used in the previous study. Briefly after the silks had emerged, open pollination was allowed, and once the silks began to show slight browning (a sign of early senescence) on the primary ear, a pure *F. graminearum* inoculum was applied. For each maize genotype, the split plot consisted of two rows (each 2 m long, with 20 seeds), planted on approximately 15 May 2017, with one randomly chosen row selected for *Fusarium* treatment, while the other row was left untreated (healthy) and separated by single guard rows. Inoculum was sprayed from ~3 cm away onto the silks, as a dose per ear of roughly 2 mL (~20,000 spores/mL). Two inoculations were applied to each *Fusarium*-treated plant within a 24 to 48 h period between 11–14 August 2017 based on maturity. Overhead misting was applied before and after inoculation and until harvest to promote infection and disease progression (0.5 min on, 8–10 min off, from 10:00 to 18:00 daily, 206 kPa, ~0.6 L of water per min).

### 2.4. Source and Preparation of F. graminearum Treatment

The source of *Fusarium* inoculum and the preparation of the spray treatment under sterile conditions were previously described [42]. To ensure successful infection under unpredictable weather conditions, three isolates of *F. graminearum,* collected from farmers’ fields in Southern Ontario, Canada, by co-author V.L.-R. were mixed to prepare the inoculum. One of the isolates was from infected maize and the other two were from wheat, and the pure strains were extensively verified for pathogenicity. Isolates of *F. graminearum* are known to infect both maize and wheat [72]. To prepare inoculum, the host seeds were surface-sterilized and plated on PDA, followed by serial subculturing onto fresh PDA plates from the leading edge of the fungal growth. The spores were harvested, diluted and spread onto water agar. Single spores were isolated and plated independently on fresh PDA. Cubes of PDA were cut from the plates and inoculated into autoclaved, modified Bilay’s medium and were incubated in flasks for 3–5 d shaking at 25 °C. The purity of the fungal spores was confirmed, and the concentration of the spores was adjusted using a hemocytometer and dilution. The final spore spray treatment was produced by combining equal amounts of the three *F. graminearum* isolates to a final concentration of ~20,000 spores per mL. A surfactant, 1.0% *v*/*v* Agral ^®^ 90 (Syngenta Canada Inc.), was added to the spore mix.

**Table 1:** Summary of the pedigrees of the host maize genotypes tested in this study from the Agriculture and Agri-Food Canada breeding program (Ottawa Research and Development Centre, Canada). Adapted from Khalaf et al., [42], with additional notes from [73,74,75].

Inbred/Hybrid Line	Year Released	Derivation	Heterotic Group	Heterotic Group Description	Grain Type	Days to Silking	* Gibberella Ear Rot/(Silk)	* Gibberella Ear Rot/(Kernel)
**CO462**	2016	CO388 × W153R	BSSS/Minnesota 13	US Hybrid era stiff stalk/Minnesota US Pre-Hybrid era	Dent	75	S	S
**CO452**	2014	(CO388 × CO328) × CO388(4)	BSSS	US Hybrid era stiff stalk	Dent	80	I	I
**CO444**	2007	S1381 × CO382	European flint	European flint, Pre-Hybrid era	Flint	79	I	I
**CO448**	2012	CO273 × CO431	P3990/Iodent	Pioneer Hybrid/US Pre-Hybrid era Corn Belt Dent	Dent	70	I	I
**CO325**	1991	(CO256 × CO264) × CO264 (2)	Early Butler	New York, Pennsylvania US Pre-Hybrid era	Dent	76	I	I
**CO449**	2012	CO432 × CO433	Minnesota 13	Minnesota US Pre-Hybrid era	Dent	75	MR-R	MR-R
**CO441**	2002	Jacques 7700 × CO298	Lancaster	Pennsylvania US Pre-Hybrid Corn Belt Dent	Dent	72	R	R
**CO431**	1999	Fusarium Resistant Synthetic	Iodent	US Pre-Hybrid Corn Belt Dent	Dent	71	R	I
**CO433**	2000	Pride K127	Minnesota 13	Minnesota US Pre-Hybrid era	Dent	77	R	R
**CO430**	1999	Fusarium Resistant Synthetic	P3990	Pioneer Hybrid	Dent	69	HR	HR
**CO432**	2000	Fusarium Resistant Synthetic C1	Minnesota 13	Minnesota US Pre-Hybrid era	Dent	74	HR	I
**P35837◊**	NA	NA	NA	Pioneer Hybrid	Dent	NA	NA	NA
**P38157◊**	NA	NA	NA	Pioneer Hybrid	Dent	NA	NA	NA
**P9855HR◊**	NA	NA	NA	Pioneer Hybrid	Dent	NA	NA	NA

* Abbreviations: **◊** = commercial Pioneer hybrid; S = sensitive; I = intermediate; MR = moderately resistant; R = resistant; HR = highly resistant; NA = Not Available.

## 3. Results

### 3.1. Overview of Culturing, Sequencing, and Taxonomy

**Figure 1:**
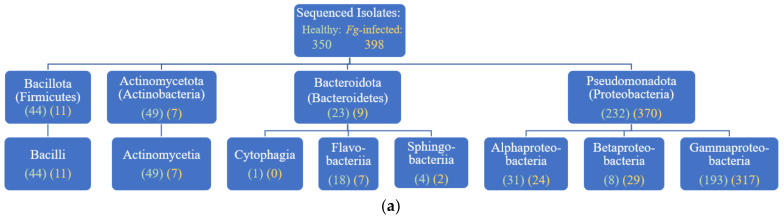

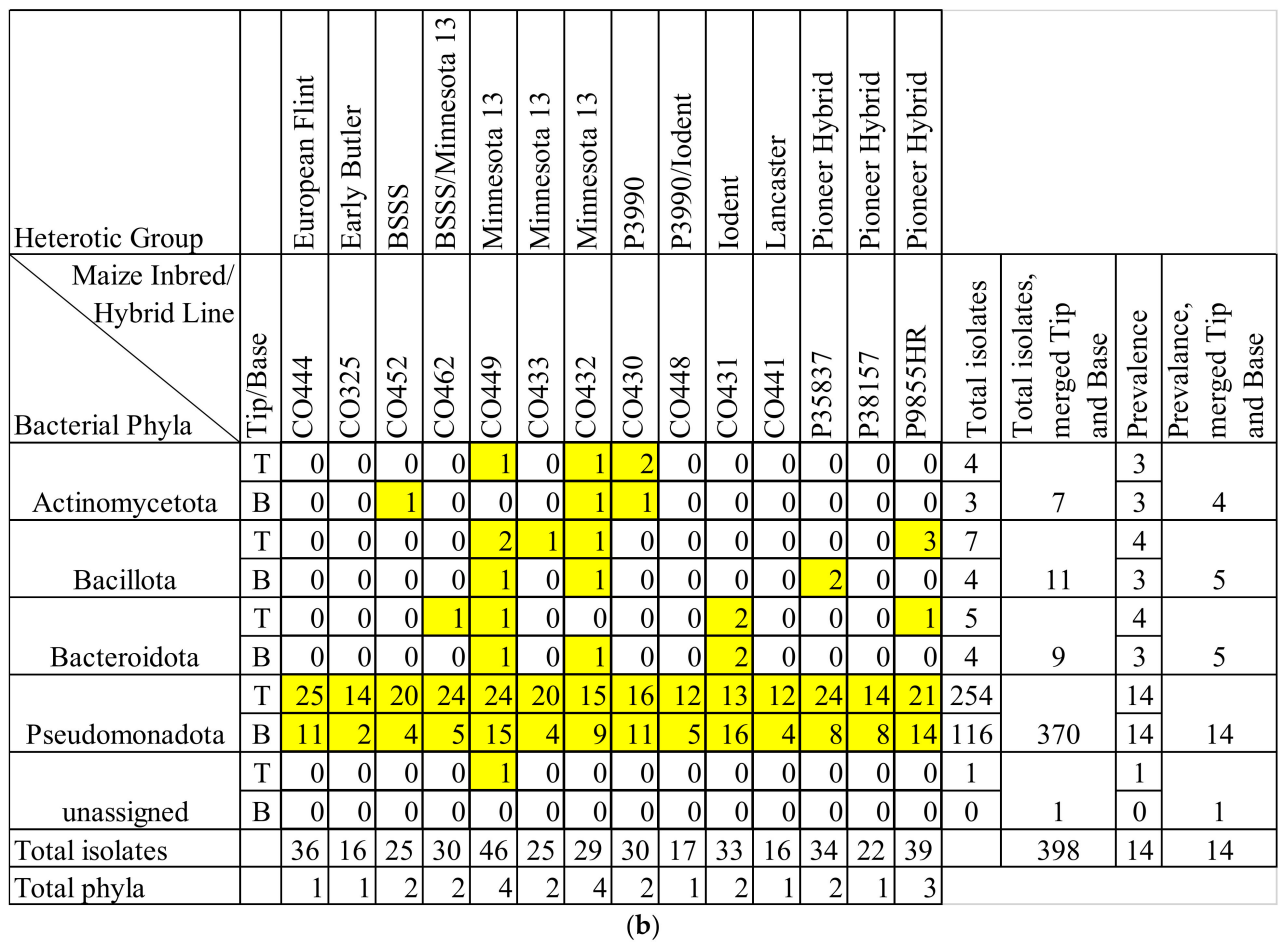
Taxonomic overview of cultured isolates. (**a**) Taxonomic overview tree of bacteria cultured from pollinated healthy (green text) and *Fusarium graminearum*-infected (orange text) maize silks (tip and base combined) to the class level. Numbers indicate the total number of isolates within each category. Two isolates from non-infected silks and one isolate from *F. graminearum*-infected silks were unassigned at the phylum level and thus were not included in the overview. (**b**) Number of isolates belonging to the cultured *F. graminearum*-infected transmitting silk microbiome at the phylum taxonomic level. Isolates were cultured separately from the tip (T) and base (B) of maize silks spanning diverse host inbred/hybrid lines and heterotic groups. Yellow cells indicate the presence of isolate(s). Prevalence refers to the number of maize genotypes that gave rise to at least one cultured isolate from that phylum. Further details are included in Table S1.

In total, 430 bacterial isolates from *F. graminearum*-treated transmitting silks (husk-contained, tip and base separated) of 14 genotypes of field grown maize were sequenced following amplification with 16S rRNA primers (Figure S1 and Table S1). Following filtering (removing sequences of <500 base pairs or >5% N’s), 398 isolates remained. There were 271 isolates from the silk tips, and 127 were from silk base samples. These isolates spanned four phyla, seven classes, 15 orders, and 22 families (Figure 1 and Figure S2–S4). The phylum Pseudomonadota overwhelmed the library of *F. graminearum*-infected silk microbes: it comprised 93% of the isolates (370/398) and was found in every tissue sample (Figure 1). The majority of isolates belonged to the class Gammaproteobacteria (317 isolates, from 14/14 genotypes), while the classes Alphaproteobacteria and Betaproteobacteria were also relatively prevalent, isolated from 10 and eight of the 14 maize genotypes, respectively.

The orders Enterobacterales (213 isolates, across 14 genotypes), Xanthomonadales (62 isolates across 13 genotypes), and Pseudomonadales (38 isolates across 12 genotypes) were the most commonly cultured and prevalent.

The most commonly cultured families included *Erwiniaceae* (103 isolates across 13 genotypes), *Enterobacteriaceae* (98 isolates across 14 genotypes), and *Xanthomonadaceae* (62 isolates across 13 genotypes). Also prevalent were *Pseudomonadaceae* (29 isolates, 12 genotypes) and *Comamonadaceae* (28 isolates, 8 genotypes).

**Figure 2:**
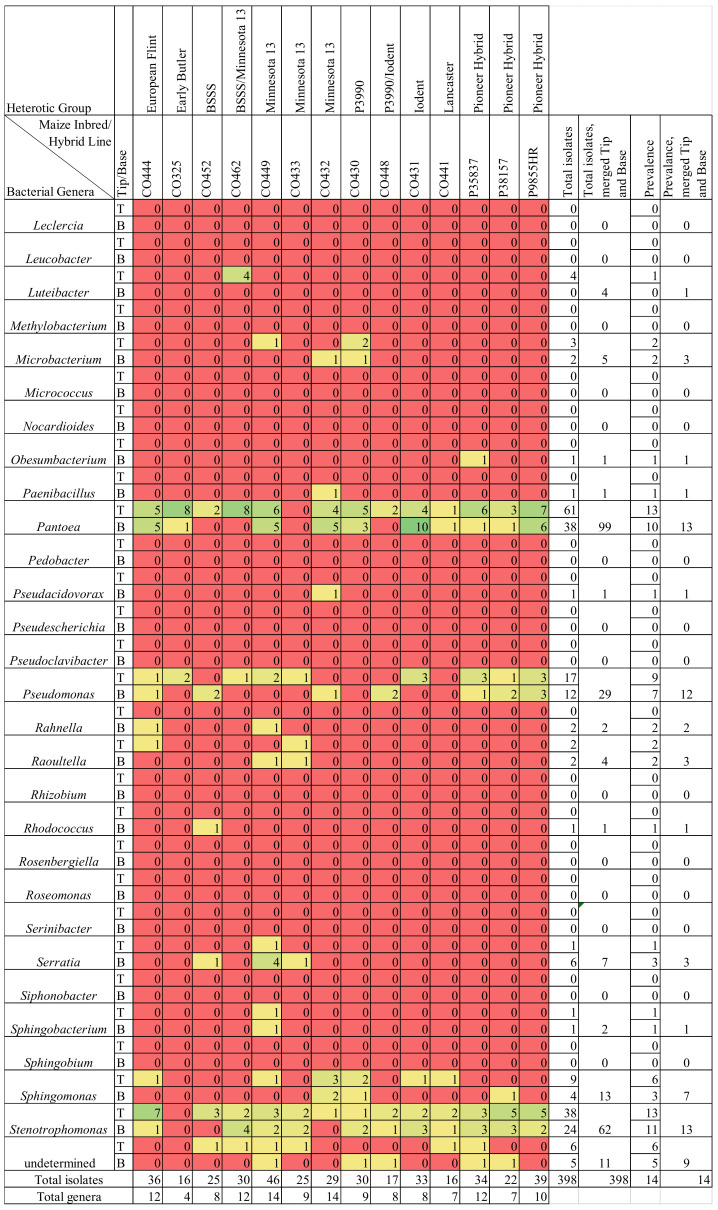
Number of isolates belonging to the cultured *Fusarium graminearum*-infected transmitting silk microbiome at the genus taxonomic level. Isolates were cultured separately from the tip (T) and base (B) of maize silks spanning diverse host inbred/hybrid lines and heterotic groups. Yellow-to-green cells indicate the presence of isolate(s). Red cells indicate the absence of isolates. Prevalence refers to the number of maize genotypes that gave rise to at least one cultured isolate from that genus. Further details can be found in Table S1.

The *F. graminearum*-treated silk bacterial library contained 35 genera based on moderate stringency thresholds, or 30 genera based on high stringency thresholds (Figure 2 and Figure S5). Combining isolates from the tip and the base tissues, commonly cultured genera included *Stenotrophomonas* (range 53–62 isolates, depending on stringency thresholds), *Klebsiella* (range 43–52 isolates), *Pantoea* (range 27–99 isolates), *Pseudomonas* (29 isolates at both thresholds) and *Delftia* (range 16–18 isolates) (Figure S5). *Stenotrophomonas* and *Pantoea* were cultured from 13 genotypes (24/28 and 23/28 tissue samples, respectively), *Pseudomonas* was cultured from 12 genotypes, and *Klebsiella* was cultured from 11 genotypes.

The *F. graminearum*-treated silk bacterial library contained 71 species based on moderate stringency thresholds or 49 species based on high stringency thresholds. Combining isolates from the tip and the base tissues, commonly cultured species included *Pantoea ananatis* (range 6–46 isolates), *Stenotrophomonas pavanii* (range 30–41 isolates), *Pantoea agglomerans* (range 4–32 isolates), *Klebsiella aerogenes* (range 19–25 isolates), *Delftia acidovorans* (range 14–17 isolates), and *Klebsiella grimontii* (16 isolates at both thresholds) (Figures S6 and S7). The most prevalent species, *Pantoea ananatis* and *Stenotrophomonas pavanii*, were cultured from 13/14 genotypes. 

Moving forward with the rest of the Results, it was decided to use a single set of stringency threshold criteria with respect to improving the clarity of taxonomic predictions, because key objectives of the paper were comparative (e.g., tip versus base silk microbiota; healthy versus *F. graminearum*-infected silk microbiota). Only those sequences that had high-quality reads were accepted for taxonomic analysis (moderate or high stringency), yet the high stringency criteria were found to exclude many isolates from being assigned a genus and a species-level classification. This was due to the high stringency threshold requiring the first match to have either a 100% identity match, or at least five more base pair matches than the next best genus predicted in NCBI (and at least 98.5% identity or the genus to be consistent in the top 20 results) to assign it to the genus of the top match; ≥1–2 mismatches were required at the species level. These exclusions appeared to be artifacts for highly prevalent genera such as *Pantoea,* which have high diversity within the genus, but low 16S sequence diversity between species of that genus, causing lower percent matches at the genus level while simultaneously reducing the power to distinguish between, and hence assign, species classification. For these reasons, it was decided to use the moderate stringency criteria (focused on top-match results from the NCBI database) for the remainder of the manuscript and the comparative analyses.

### 3.2. Silk Tip versus Base Comparisons

**Figure 3:**
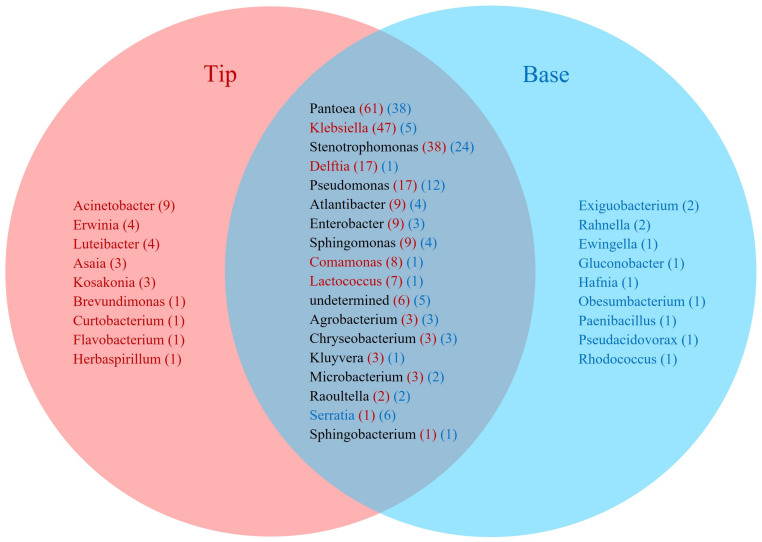
Comparison of bacterial genera which were cultured from the tip and/or the base tissues of *F. graminearum*-treated transmitting maize silks spanning diverse host inbred/hybrid lines and heterotic groups. Numbers in brackets indicate the number of isolates cultured: red from the tip and blue from the base. Further details are in Table S1.

**Figure 4:**
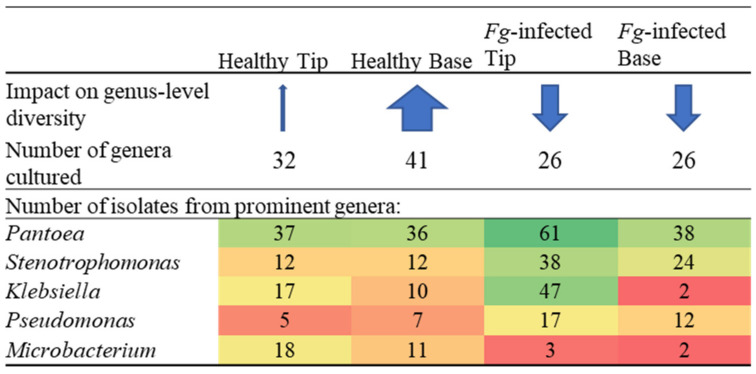

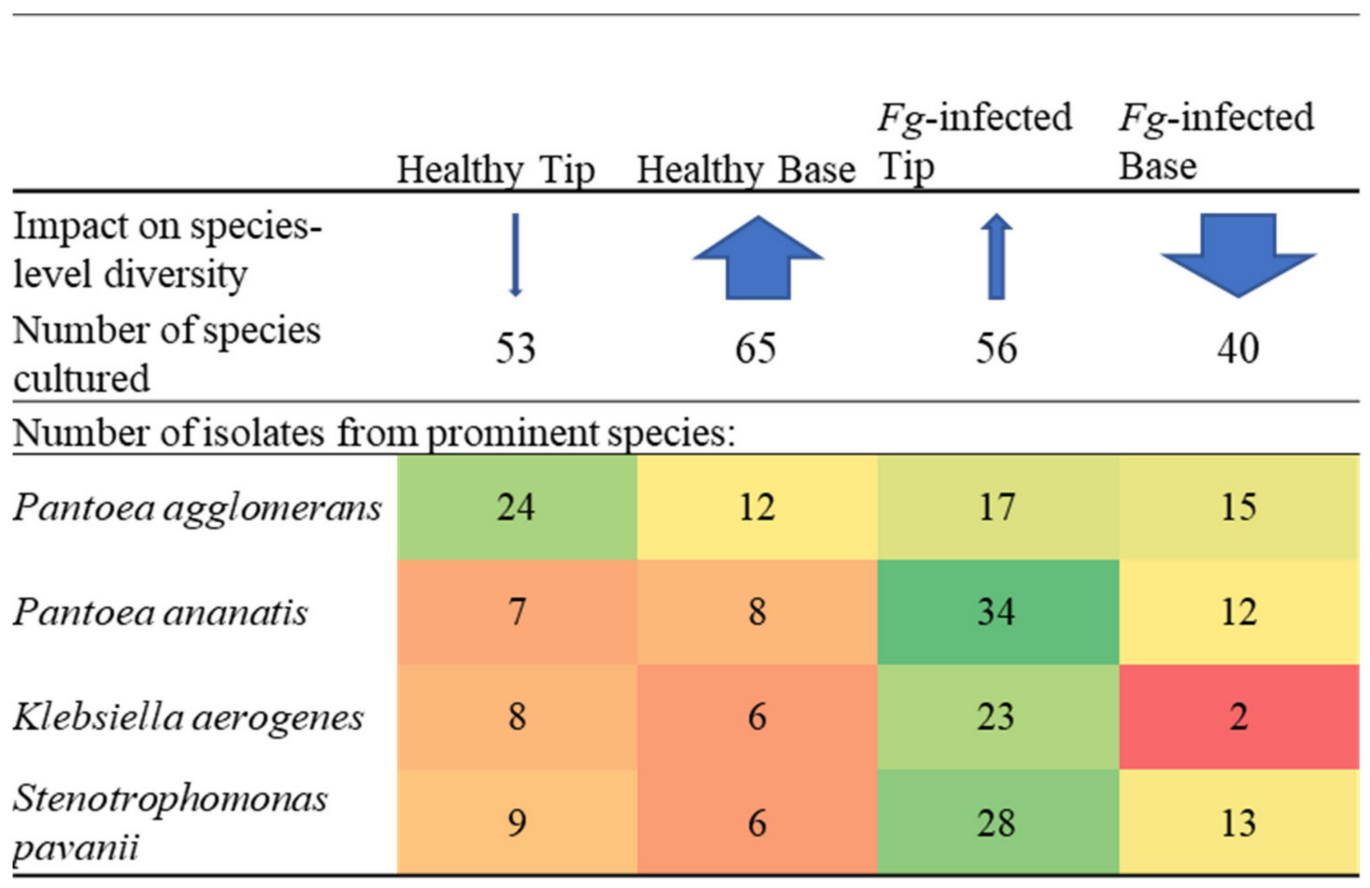
Genus- and species-level summary: Effects of *Fusarium graminearum* (*Fg*) treatment and tip versus base location on the diversity of the transmitting silk cultured microbiome. The thickness of the blue arrows represents the relative impact on the number of genera or species present in each category. The values of the numbers were denoted by a colour spectrum from red (low) to green (high).

**Figure 5:**
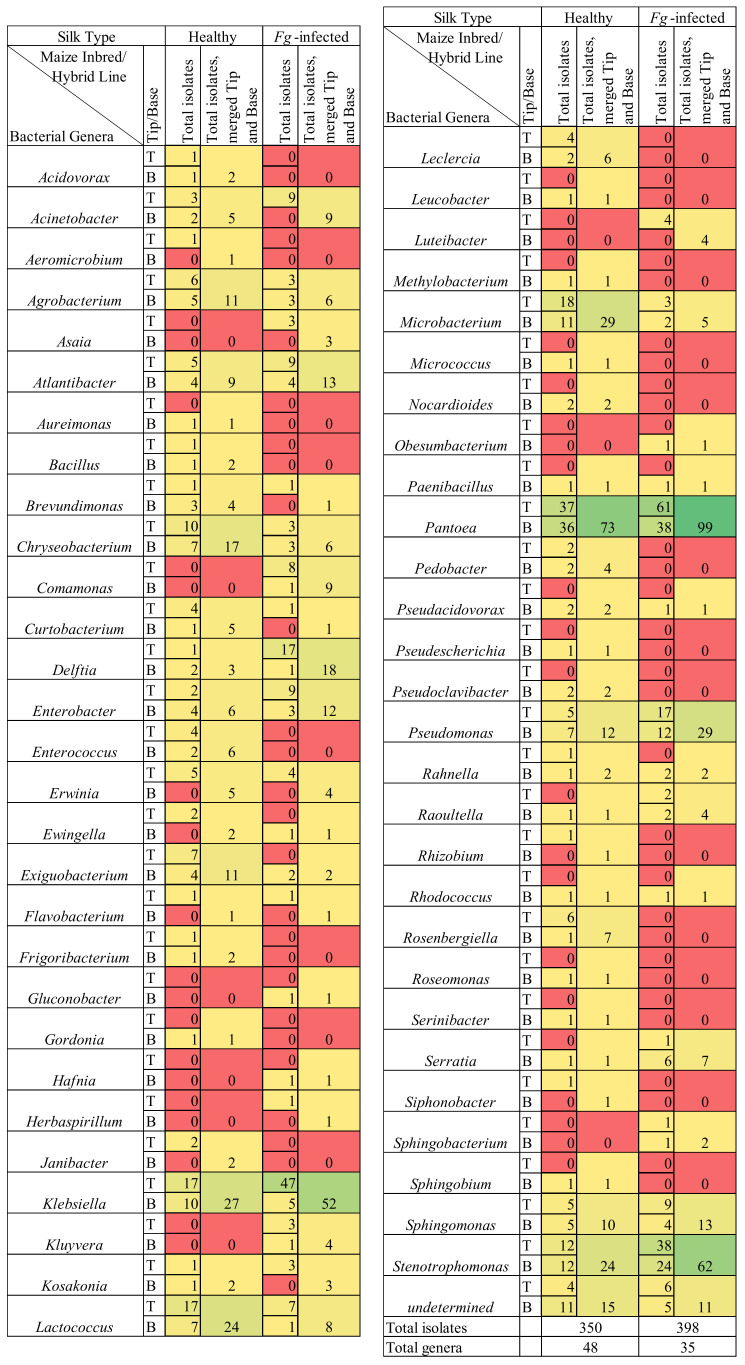
Comparison of the number of isolates belonging to the cultured healthy (non-infected) and the Fusarium graminearum-infected transmitting silk microbiomes at the genus taxonomic level. Isolates were cultured separately from the tip (T) and the base (B) of maize silks spanning diverse host inbred/hybrid lines and heterotic groups. Yellow-to-green cells indicate the presence of isolate(s). Red cells indicate the absence of isolates. Further details are found in Table S1.

Overall, there were more isolates cultured from the tip than the base of transmitting silks infected with *F. graminearum* (Figure 3). This was reflected at all taxonomic levels, from phyla to species (Figure 1b, Figure 4, Figure 5 and Figure S2–S4). Within each phylum, more isolates were cultured from the tip than from the base, most notably within Pseudomonadota where there were 254 isolates from the tip tissues, and 116 isolates from the base tissues (Figure 1b). At the order level, this pattern was strongly shown in Burkholderia (29 isolates from the tip, 3 from the base), Enterobacterales (144 isolates from the tip, 69 from the base), Pseudomonadales (26 isolates from the tip, 12 from the base), and Enterobacterales (38 isolates from the tip, 24 from the base) (Figure S3). A prominent example at the species level was *Delftia acidovorans,* which was cultured 17 times from tip samples, but never from base samples (Figure S7). Overall, the *F. graminearum*-treated bases contained 110 cultured OTUs, while the tips contained 189, and healthy base samples contained 165 cultured OTUs, while the tips contained 177.

### 3.3. Maize Genotype Comparisons

Transmitting silks of maize genotypes CO449 and CO432, belonging to the heterotic group Minnesota 13, gave rise to the greatest cultured bacterial diversity after infection with *F. graminearum*. They were the only genotypes to produce isolates from all four phyla, often in both the tip and the base (Figure 1b). These two genotypes gave rise to cultured bacteria from 10 and nine bacterial orders, respectively, and they each covered 12 bacterial families and 14 genera. Interestingly, the class Sphingobacteriia only appeared in the genotype CO449 (Minnesota 13); unique isolates of the genus *Sphingobacterium* appeared in both the tip and the base of this host genotype (Figure 2 and Figure S2).

One genotype of maize, CO325 (from the Early Butler heterotic group), contained low cultured bacterial diversity in *F. graminearum*-infected transmitting silks. This genotype only produced isolates from the class Gammaproteobacteria, the orders Enterobacterales and Pseudomonadales, and gave rise to only three families and four genera.

### 3.4. Comparing Taxonomy of Healthy and F. graminearum-Infected Silks

When comparing the diversity of the microbes cultured from the healthy and the *F. graminearum*-infected transmitting silks, specific taxa increased and decreased in frequency, but overall, a ~20–25% reduction in diversity at different taxonomic levels was observed in the *F. graminearum*-infected silks (Figure 4), while the remaining isolates increased in number. Despite the number of isolates increasing from 350 in the healthy silks to 398 in the *F. graminearum*-infected silks, the total number of families cultured were reduced from 28 in the healthy silks to 23 in the *F. graminearum*-infected silks; the total number of genera were reduced from 48 in the healthy silks to 35 in the *F. graminearum*-infected silks; and the total number of species were reduced from 94 in the healthy silks to 71 in the *F. graminearum*-infected silks. However, the differences in the cultured microbiomes between these two treatments were even greater, as the taxa that were present in *F. graminearum*-infected silks were often different than those in healthy silks.

Specifically, compared to the healthy silks, the *F. graminearum*-infected silks had fewer isolates cultured from the phyla Bacillota, Actinomycetota, and Bacteriodota, and more isolates from Pseudomonadota (Figure 1b). Furthermore, there was a class-level shift towards more Betaproteobacteria (2% of isolates in the healthy silks versus 7% in the *F. graminearum*-infected silks) and especially more Gammaproteobacteria (55% of isolates in the healthy silks versus 80% in the *F. graminearum*-infected silks) (Figure 1 and Figure S2). The cultured microbiome of the *F. graminearum*-infected silks contained bacteria from the order Lysobacterales (Figure S3), which were not found in the non-infected silks [70]. Conversely, the cultured microbiome of the *F. graminearum*-infected silks was lacking bacteria from the orders Cytophagales and Propionibacteriales (Figure S3), which were cultured from the non-infected silks [70]. There were nine bacterial families (*Alcaligenaceae*, *Aurantimonadaceae*, *Beutenbergiaceae*, *Enterococcaceae*, *Gordoniaceae*, *Intrasporangiaceae*, *Methylobacteriaceae*, *Micrococcaceae* and *Spirosomaceae*) which were cultured from the healthy silks, but not the *F. graminearum*-treated silks, whereas there were only three families (*Hafniaceae, Oxalobacteraceae* and *Rhodanobacteraceae*) exclusively cultured from the *F. graminearum*-treated silks (Figures S4 and S8). The number of isolates cultured from the families *Microbacteriaceae*, *Bacillaceae*, *Streptococcaceae* and *Weeksellaceae* were notably reduced in the *F. graminearum*-treated silks (Figure S8). In contrast, specific families were cultured more frequently in the *F. graminearum*-treated silks, notably *Comamonadaceae*, *Enterobacteriaceae*, *Erwiniaceae*, *Pseudomonadaceae* and *Xanthomonadaceae*.

**Figure 6:**
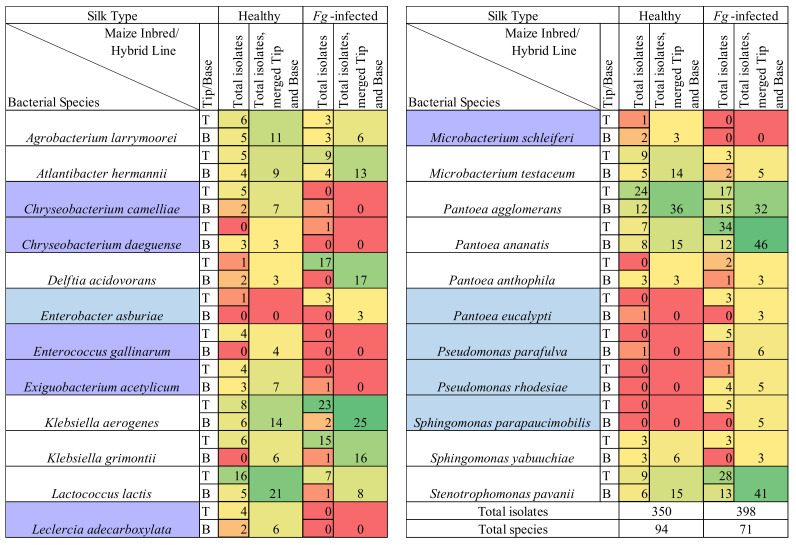
Comparison of number of isolates belonging to the cultured healthy (non-infected) and *Fusarium graminearum*-infected transmitting silk microbiome at the species taxonomic level. Isolates were cultured separately from the tip (T) and the base (B) of maize silks spanning diverse host inbred/hybrid lines and heterotic groups. Yellow-to-green cells indicate the presence of isolate(s). Red cells indicate the absence of isolates. Only species which were cultured from three or more maize genotypes in either healthy or *F. graminearum*-infected silks were included in this figure. Blue cells indicate the species which met this threshold in *F. graminearum*-treated silks but not in healthy silks. Purple cells indicate the species which met this threshold in healthy silks but not in *F. graminearum*-treated silks. Further details are in Table S1.

There were 22 bacterial genera which were cultured from the healthy silks, rather than the *F. graminearum*-treated silks, whereas there were only nine genera exclusively cultured from the *F. graminearum*-treated silks (Figure 2 and Figure 5). Among the overlapping genera, the number of isolates cultured from *Chryseobacterium*, *Exiguobacterium*, *Lactococcus* and *Microbacterium*, were notably reduced in the *F. graminearum*-treated silks (Figure 5). In contrast, specific genera were cultured more frequently in the *F. graminearum*-treated silks, most notably *Delftia*, *Klebsiella*, *Pantoea*, *Pseudomonas* and *Stenotrophomonas*. Many prevalent species were different from the healthy silks as compared to the *F. graminearum*-treated silks. There were six species (*Chryseobacterium camelliae*, *Chryseobacterium daeguense*, *Enterococcus gallinarum*, *Exiguobacterium acetylicum*, *Leclercia adecarboxylata* and *Microbacterium schleiferi*) that were cultured from at least three maize genotypes in healthy silks but never cultured from the *F. graminearum*-treated silks; by contrast there were five species (*Enterobacter asburiae*, *Pantoea eucalypti*, *Pseudomonas parafulva*, *Pseudomonas rhodesiae* and *Sphingomonas parapaucimobilis*) that were cultured from at least three genotypes in the *F. graminearum*-infected silks that were never cultured from the healthy silks (Figure 6, purple and blue cells). Some prevalent species were cultured less frequently in the *F. graminearum*-treated silks, including *Lactococcus lactis* and *Microbacterium testaceum.* In contrast, some prevalent species were cultured more frequently in the *F. graminearum*-treated silks, including *Delftia acidovorans*, *Klebsiella aerogenes*, *Klebsiella grimontii*, *Pantoea ananatis* and *Stenotrophomonas pavanii*. It appears that the impact of the *F. graminearum* infection and the distance from the site of pathogen inoculation may be species-specific, perhaps with select species increasing in the *F. graminearum*-infected silk tips (Figure 4).

### 3.5. F. graminearum-Treated Transmitting Silk Taxonomy at the Cultured OTU Level

**Figure 7:**
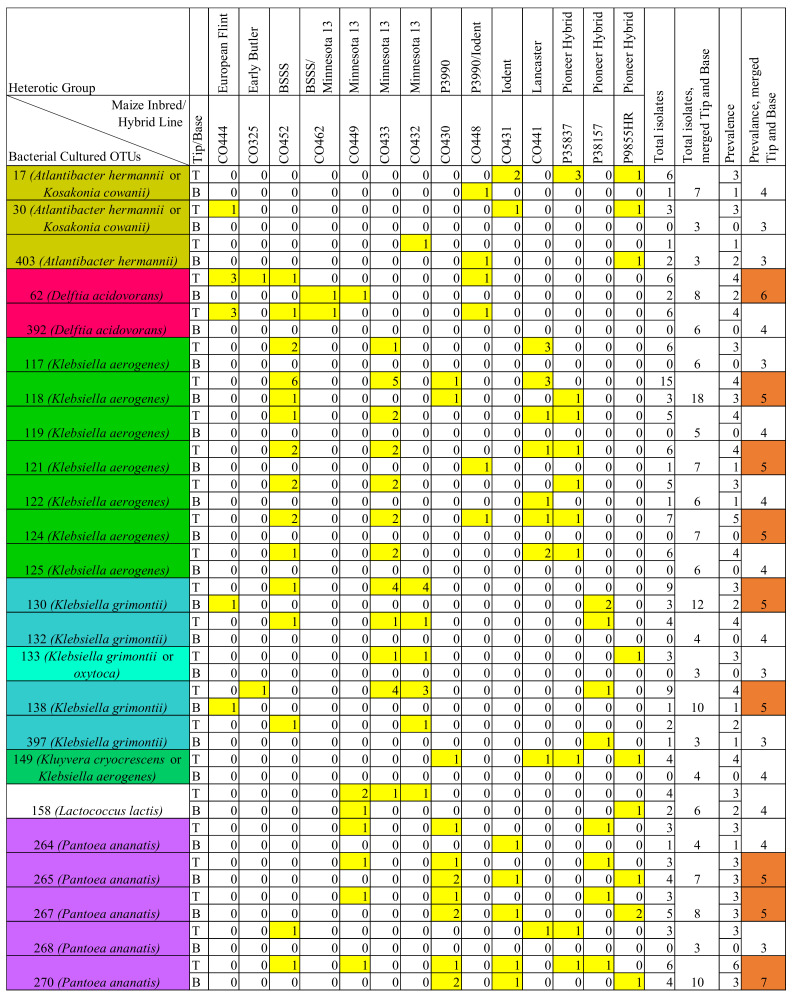

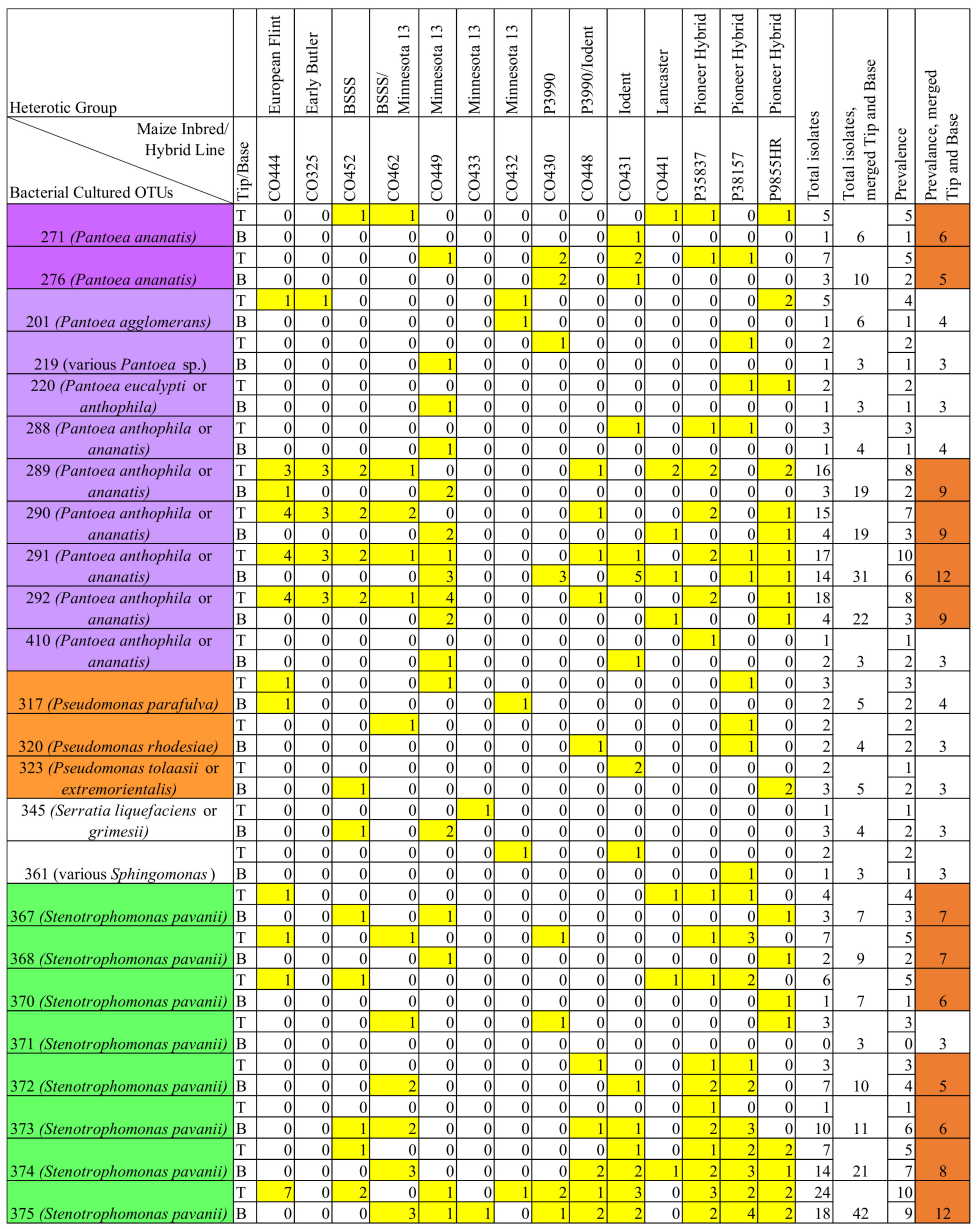
Number of isolates from prevalent cultured OTUs identified from the *Fusarium graminearum*-infected transmitting silks. Isolates were cultured separately from the tip (T) and the base (B) of maize silks spanning diverse host inbred/hybrid lines and heterotic groups. Only OTUs which were found in three or more genotypes were included. Yellow cells indicate the presence of isolate(s). Orange cells indicate cultured OTUs which occurred in five or more host genotypes. The colors in the leftmost column indicate OTUs that were grouped by genus and/or species. Prevalence refers to the number of maize genotypes that gave rise to at least one cultured isolate from that OTU. Further details are in Table S1.

When combining all samples, the *F. graminearum*-treated silks produced 238 OTUs. This number was based on OTU assignments in which the healthy and the *F. graminearum*-infected silks were analyzed together as a complete group. The OTUs were based on two sequence lengths, because, as described in Supplementary Text S1, the primer pair was changed from 799F/1492R to 27F/1492R midway through the experiment to allow for direct comparison between the cultured sequences and earlier MiSeq OTUs from Khalaf et al. [42]. Of the 238 OTUs in the *F. graminearum*-treated silks, 143 OTUs were cultured from only one genotype, 47 OTUs were cultured from two genotypes and 48 OTUs were cultured from three or more genotypes (Figure 7 and Figure S9). Specific species contained high strain-level diversity, such as *Pantoea agglomerans* and *P. ananatis*, which contained 27 and 25 OTUs, respectively (Figure S9). Some species displayed greater uniformity (e.g., *S. pavanii*), with the same OTUs being cultured across genotypes and the tip and the base samples. 

In terms of prevalence across host genotypes, the most prevalent cultured OTUs in the *F. graminearum*-treated silks belonged to *Pantoea* (mainly *P. ananatis*, or a close match between *P. ananatis* and *P. anthophila*), *Stenotrophomonas pavanii*, and *Klebsiella aerogenes* (Figure 7). The most commonly cultured and prevalent OTUs were OTU 375 (a taxon of *S. pavanii* with 42 isolates across 12 genotypes) and OTU 291 (a taxon of *P. anthophila* or *P. ananatis* with 31 isolates across 12 genotypes) (Figure 7 and Figure S9). OTU 375 appeared across all heterotic groups except Early Butler and Lancaster, while OTU 291 appeared across all heterotic groups: European Flint, Early Butler, Iodent, Lancaster, BSSS, BSSS/Minnesota 13, Minnesota 13, P3990, P3990/Iodent and Pioneer Hybrid. 

OTU 375 was cultured rather evenly from both the tip and the base samples (Figure 7). Specific OTUs were found more frequently in the base, such as OTU 373 and OTU 374 (both *S. pavanii*), while other OTUs were found more frequently in the tip, such as OTU 118 (*Klebsiella aerogenes*), as well as OTUs 289, 290 and 292 (all three *Pantoea anthophila* or *P. ananatis*) (Figure 7).

Of the aforementioned 143 OTUs which were isolated from only one genotype in the *F. graminearum*-treated silks, 20 were cultured multiple times (2–4 isolates). Some of these OTUs were cultured from both the tip and base of the single genotype (OTUs 143, 190, 230, 262, 322, 328), perhaps suggesting colonization of the full length of the silk tissue; others were exclusively cultured from either the tip or the base. For example, OTU 75 and OTU 170 were cultured four times from the tip of their respective genotype.

Genotype CO432 had high diversity overall (as noted above) but had a lower frequency of prevalent OTUs (17 occurrences), which indicates a greater incidence of unique isolates (Figure 7). The three commercial hybrids had a relatively high number of prevalent cultured OTUs (35, 41, 42) (Figure 7). A commercial hybrid, P9855HR, contained the most OTUs (55), while CO325 (Early Butler) contained the fewest OTUs (18) (Figure S9).

### 3.6. Comparison of Prevalent OTUs in Healthy versus F. graminearum-Treated Silks

As noted above, the *F. graminearum*-treated silks produced 238 OTUs, while the healthy silks gave rise to 271 OTUs, when the two populations were analyzed together as a complete group. Because of overlap between the two treatments, the total number of unique OTUs was 389. The number of OTUs from the healthy silks when analyzed alone was 221 [70], compared to 271 here; this discrepancy is due to the fact that some 16S sequences from the healthy silk population were assigned to additional OTUs when analyzed alongside the *F. graminearum*-treated silks because they matched multiple longer or higher quality 16S sequences from the *F. graminearum*-treated population that were revealed to be distinct. After the assignments were updated, healthy silks gave rise to 26 OTUs which were found in three or more genotypes, whereas the *F. graminearum*-treated silks produced 48 OTUs with this level of prevalence.

**Figure 8:**
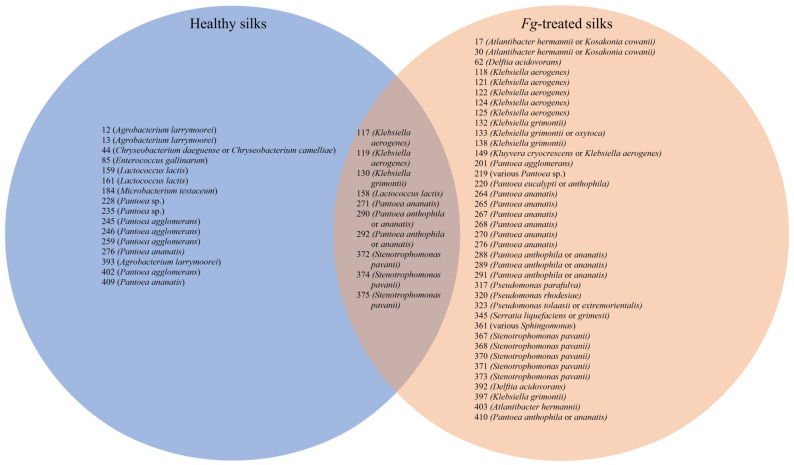
Comparison of cultured OTUs which were prevalent in untreated (healthy) transmitting silks, *Fusarium graminearum*-treated transmitting silks and those which were prevalent in both. Transmitting maize silks spanned diverse host inbred/hybrid lines and heterotic groups. Prevalent cultured OTUs were those that occurred in three or more maize genotypes. The tip and the base isolates were analyzed together. Further details are found in Table S1.

In terms of conservation, some OTUs were prevalent in both the healthy and the *F. graminearum*-treated transmitting silks, including OTUs 117, 119, 130, 158, 271, 276, 290, 292, 372, 374 and 375 (Figure 8). OTU 375 was the most prevalent cultured OTU in the healthy silks (across six genotypes), and the most prevalent and frequently cultured in *F. graminearum*-infected silks (across 12 genotypes). Combining the healthy and the *F. graminearum*-infected silks, OTU 375 was retrieved from 13/14 genotypes.

The *F. graminearum*-treated silks introduced prevalent OTUs belonging to new species (compared to the prevalent OTUs in healthy silks), including *Delftia acidovorans* which was exclusively cultured from the tips of the *F. graminearum*-treated silks, as noted above.

### 3.7. Comparison of V4-MiSeq Predicted Taxa and Isolates Cultured from the Transmitting Silk Microbiome

#### 3.7.1. Overview

Previously, members of our research group [42] used 16S V4-MiSeq (~254 bp) to define the transmitting silk microbiome of maize in terms of core taxa (V4-MiSeq core) and taxa that were induced after treatment with *F. graminearum* (*F. graminearum*-indicators). As described in the Materials and Methods, on the day of the 2017 harvest, the silk tissues were divided, and a subset was frozen with glycerol to allow culturing for this study, while the remainder was frozen without glycerol and underwent DNA extraction for the short-length V4-MiSeq study. Khalaf et al. [42] predicted, using V4-MiSeq, that the transmitting silks had a stable and abundant core microbiome (11 members), from which some members increased in abundance with the *F. graminearum* treatment (seven of the 11 core members); there were also non-core members that increased in abundance following the *F. graminearum* treatment (10 members). The *F. graminearum*-induced V4-MiSeq taxa that were also prevalent were termed the *F. graminearum*-indicators.

**Figure 9:**
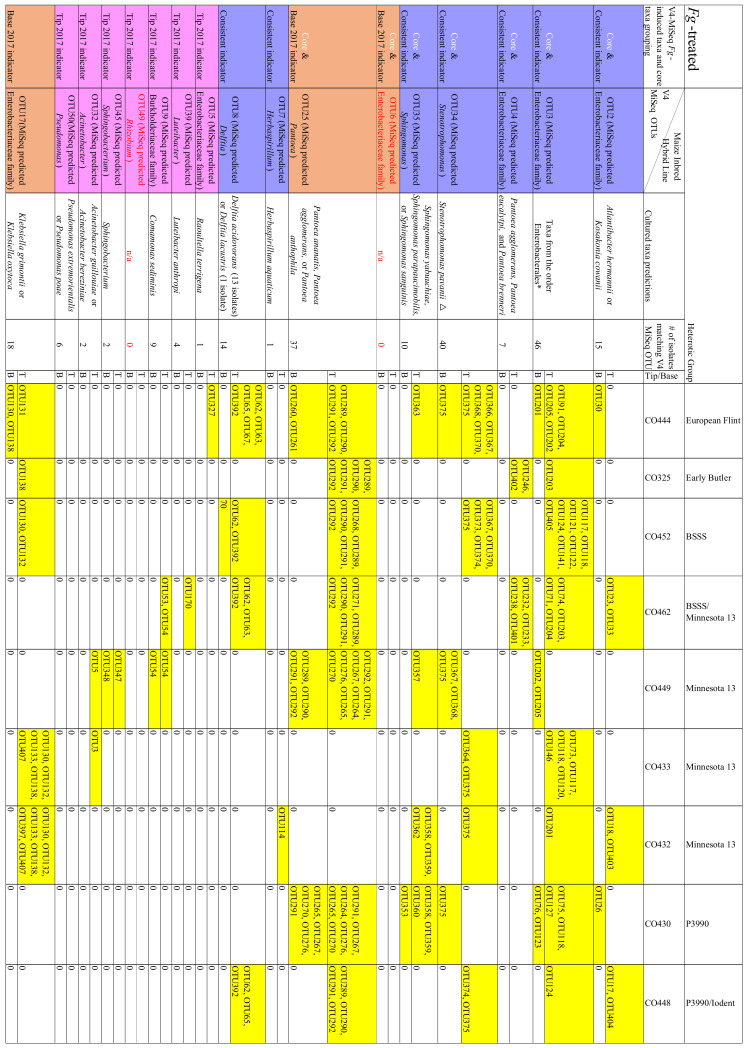
Comparison of the *Fusarium graminearum*-induced V4-MiSeq OTUs (*F. graminearum*-indicators) to matching cultured OTUs isolated from the *F. graminearum*-treated transmitting silks of maize. Isolates were cultured separately from the tip (T) and the base (B) of maize silks spanning diverse host inbred/hybrid lines and heterotic groups. V4-MiSeq *F. graminearum*-induced OTUs (noted as *F. graminearum* indicators in extreme left column) from Khalaf et al. [42] were taxa which had elevated abundance in the transmitting silks treated with *F. graminearum* compared to the untreated silks (healthy silks); it is indicated whether these taxa were elevated in the 2017 silk tip or base tissues exclusively, or consistent indicators across the tip tissues in both 2016 and 2017. Some of the *F. graminearum*-induced indicator taxa were also members of the predicted core transmitting silk microbiome from Khalaf et al. [42] (white text in extreme left column). The asterisk (*) indicates that Enterobacterales isolate predictions include *Pantoea agglomerans*, *Klebsiella aerogenes*, *Klebsiella variicola*, *Klebsiella pneumoniae*, *Enterobacter asburiae*, *Enterobacter ludwigii*, *Erwinia persicina*, *Enterobacter mori*, and *Erwinia aphidicola*. Yellow cells indicate the presence of isolate(s). The triangle (Δ) indicates that 37 of the isolates matching V4-MiSeq OTU 34 were clearly identified as *Stenotrophomonas pavanii*, while two isolates had equal first matches to *S. pavanii* and *S. maltophilia*, and a remaining isolate had a lower (96.45%) first match to *S. maltophilia*. Thus, *S. pavanii* is a high confidence taxonomic prediction for V4-MiSeq OTU 34 based on sequence data of 40 cultured isolates. Red text indicates that no cultured isolate matched the V4-MiSeq OTU. Prevalence refers to the number of maize genotypes that gave rise to at least one cultured isolate from that OTU. Further details are found in Table S1.

The change in 16S primers (for individually cultured isolates) from 799F/1492R to 27F/1492R midway through the study allowed for direct comparison between many cultured isolates and the V4-MiSeq taxa. Of the earlier 11 core taxa, isolates matching eight were cultured from both the *F. graminearum*-treated and the healthy silks (Figures S10 and S11). Of the earlier seven *F. graminearum*-induced taxa that also belonged to the predicted core microbiome based on V4-MiSeq data, isolates matching six were cultured from the *F. graminearum*-treated and/or the healthy silks (Figure 9 and Figure S12). The exception was V4-MiSeq OTU 6 (core and base 2017 *F. graminearum*-indicator) which was not cultured in this study. Of the earlier 10 non-core *F. graminearum*-indicator taxa, nine were cultured from the *F. graminearum*-treated silks and two were cultured from the healthy silks.

#### 3.7.2. Improved Species Level Taxonomic Resolution within the Previously Defined V4-MiSeq Transmitting Silk Microbiome for Core Taxa and *F. graminearum*-Induced Taxa

Given the short 16S reads and subsequently low taxonomic resolution of V4-MiSeq (to the genus or family level), here the taxonomy was clarified using longer-length 16S sequences from the cultured isolates in this study (Figure 9 and Figure S10–S12). For example, V4-MiSeq OTU 9 was previously identified broadly as belonging to the *Burkholderiaceae* family, but longer 16S sequencing from cultured isolates revealed nine matching isolates from *F. graminearum*-treated silks, all of which were identified as *Comamonas sediminis* (Figure 9). Likewise, V4-MiSeq OTU 17 was previously identified broadly as being a member of the *Enterobacteriaceae* family, but longer 16S sequencing from cultured isolates revealed 18 matching isolates from *F. graminearum*-treated silks and five additional matching isolates from healthy silks, all of which were identified as *Klebsiella grimontii* or *Klebsiella oxytoca* (Figure 9 and Figure S12). 

#### 3.7.3. Diversity amongst Cultured OTUs That Matched Previously Defined V4-MiSeq Core and *F. graminearum*-Induced Taxa in the Transmitting Silk Microbiome

The short 16S sequence of many core and *F. graminearum*-induced taxa predicted by V4-MiSeq was found to mask bacterial diversity within silks: multiple cultured OTUs were found to match individual V4-MiSeq OTUs. For example, V4-MiSeq OTU 3 matched sequences with 27 cultured OTUs from the *F. graminearum*-treated silks (representing up to 27 unique strains). An *individual sample* of the silk tips of commercial hybrid P9855HR contained 11 cultured OTUs matching core and consistent indicator V4-MiSeq OTU 2, revealing great diversity within the V4-MiSeq predictor.

#### 3.7.4. Comparing *F. graminearum*-Treated and Healthy Silk Isolates Matching V4-MiSeq Core and V4-MiSeq *F. graminearum*-Predictors

Healthy silks contained many V4-MiSeq-predicted core taxa. Interestingly, it was extremely rare for the healthy silks to contain any *F. graminearum*-indicator taxa that were not part of the core. Of the ten V4-MiSeq OTUs which were *F. graminearum*-induced taxa (but not part of the core), healthy silks contained isolates from only two: a single isolate matching 2017 tip *F. graminearum*-indicator V4-MiSeq OTU 49 *Rhizobium* in commercial hybrid P38157, and five isolates matching 2017 base *F. graminearum*-indicator V4-MiSeq OTU 17 *Enterobacteriaceae* family in CO432, CO431, and commercial hybrids P38157 and P9855HR. Of the healthy silk isolates matching V4-MiSeq OTU 17, all of the isolates found in the tips matched cultured OTU 130; in contrast, in the 18 isolates matching V4-MiSeq OTU 17 in *F. graminearum*-infected silks, 11 cultured OTUs were identified.

In terms of the effect of the host genotype, the *F. graminearum*-treated silk pools from individual maize genotypes contained isolates from as many as eight V4-MiSeq *F. graminearum*-indicator taxa (CO444, European Flint) and never fewer than four. By contrast, in healthy silks, three maize genotypes contained no V4-MiSeq *F. graminearum*-indicator taxa. Individual maize genotypes contained isolates from three to six core taxa in the *F. graminearum*-treated silks, but zero to five core taxa from healthy silks.

Isolates matching most *F. graminearum*-indicators and core taxa were more frequently isolated from the tip tissues of maize genotypes than from the base tissues (Figure 9 and Figure S10); *F. graminearum* was inoculated at the tip. 

In Khalaf et al. [42], some bacterial taxa were induced by *F. graminearum* only in the base or the tip in the 2017 field. Here, the isolates matching V4-MiSeq-predicted Base 2017 *F. graminearum* indicator taxa were found in both the tip and the base, and matches were found across many maize genotypes (Figure 9). Specifically, the cultures matching V4-MiSeq OTU 6, OTU 17, and OTU 25 were cultured from zero, seven, and 12 genotypes, respectively. By contrast, Tip 2017 *F. graminearum* indicator taxa were rarely cultured in comparison (Figure 9). Specifically, the cultures matching V4-MiSeq OTU 5, OTU 39, OTU 9, OTU 49, OTU 45, OTU 32, OTU 50 were only found in 0–2 genotypes each.

#### 3.7.5. Prevalent Individual Cultured OTUs That Matched Previously Defined V4-MiSeq Core and *F. graminearum*-Induced Taxa in the Transmitting Silk Microbiome

Since the V4-MiSeq predictions had limited resolution, an important objective of this study was to evaluate whether there are specific bacteria that constitute part of the transmitting silk core microbiome or the *F. graminearum*-indicator groups and to recover these microbes for future basic and applied studies. Some specific cultured OTUs that matched the V4-MiSeq OTUs from Khalaf et al. [42] appeared somewhat consistently across the maize genotypes, and across the healthy and *F. graminearum*-treated silks (Figure 9 and Figure S10–S12):

- Multiple prevalent cultured OTUs matched core V4-MiSeq OTU 25 (*Pantoea*), the most prevalent and abundant silk OTU identified by Khalaf et al. [42]: cultured OTU 291 was found in 11 maize genotypes; cultured OTU 289 was found in nine maize genotypes; cultured OTU 290 was found in eight maize genotypes, and cultured OTU 292 was found in eight maize genotypes and also appeared in two genotypes in healthy silks. Cultured OTU 271 (predicted as *Pantoea ananatis*) matched V4-MiSeq OTU 25 and was isolated from three maize genotypes in the healthy silks and five host genotypes in the *F. graminearum*-treated silks.
- Cultured OTU 375 (identified as *Stenotrophomonas pavanii*) matched core V4-MiSeq OTU 34, and was cultured from six maize genotypes in the healthy silks; in the *F. graminearum*-treated silks, cultured OTU 375 appeared in 12 genotypes.
- Cultured OTU 117 matched V4-MiSeq OTU 3 and was isolated from three maize genotypes in the healthy silks. Cultured OTU 117 was also cultured from three maize genotypes in the *F. graminearum*-treated silks, although only one of the genotypes (CO452, of BSSS heterotic group) overlapped with those in the healthy silks.
- There were five isolates from the *F. graminearum*-treated silks that matched V4-MiSeq OTU 28 (V4-MiSeq core taxa), and of these, four matched a single cultured OTU (OTU 317) (Figure S10). By contrast, in the healthy silks, V4-MiSeq OTU 28 was only cultured from one sample, which did not match cultured OTU 317.
- Similarly, of the isolates matching V4-MiSeq OTU 31 (V4-MiSeq core taxa), three of the four isolates from the *F. graminearum*-treated silks corresponded to a single cultured OTU (OTU 7). In the healthy silks, one of the two isolates matching V4-MiSeq OTU 31 also matched cultured OTU 7.
- Cultured OTU 246 (a taxon of *Pantoea agglomerans*) matched core V4-MiSeq OTU 4, and was cultured from four maize genotypes in healthy silks. However, cultured OTU 246 was only identified once in *F. graminearum*-treated silks.

Interestingly, in terms of the isolates that matched V4-MiSeq *F. graminearum*-induced OTUs which were not members of the core transmitting silk microbiome, the situation was different: isolates matching nearly all of these OTUs were detected in *F. graminearum*-treated silks, but scarcely any from healthy silks (Figure 9 and Figure S12). Specifically, of 10 non-core *F. graminearum*-induced taxa predicted by V4-MiSeq, many isolates matching 9 were cultured from the *F. graminearum*-infected silks (57 isolates total), while the healthy silks only contained cultures from two non-core *F. graminearum*-indicators [V4-MiSeq Base 2017 *F. graminearum*-indicator OTU 17 (5 isolates), and V4-MiSeq Tip 2017 *F. graminearum*-indicator OTU 49 (1 isolate)]. Referring to V4-MiSeq OTU 17, the matching cultured OTUs found in healthy silks (OTU 130, OTU 138, and OTU 140) were also found in the *F. graminearum*-treated silks. For V4-MiSeq OTU 49, there were no matching cultured isolates from the *F. graminearum*-treated silks.

In terms of consistency within the *F. graminearum*-treated isolates matching the non-core *F. graminearum*-predictors, cultured OTU 62 was found to match V4-MiSeq OTU 8 and was retrieved from five genotypes of *F. graminearum*-treated maize; the tips of all five genotypes contained cultured OTU 62 (Figure 9). Additionally, cultured OTU 54 was matched to V4-MiSeq OTU 9 and was found in three *F. graminearum*-treated samples. Furthermore, two cultured OTUs (OTU 130 and 138) matched V4-MiSeq OTU 17 and were each retrieved in *F. graminearum*-treated silks of five host genotypes.

The order Lysobacterales was only isolated four times from one tissue, the tip of CO462 (BSSS/Minnesota 13), and was not found in the healthy silks (Figure S3). These four isolates were all identified as cultured OTU 170 of *Luteibacter anthropi*, and all matched V4-MiSeq OTU39.

## 4. Discussion

### 4.1. Overview

Microbe–microbe interactions affect *Fusarium* infection and mycotoxin production in cereal crops, and this is an important and evolving area of research [43]. The current study improves the understanding of the maize-transmitting silk microbiome (TSM), and with improved taxonomic resolution of individual cultured bacteria, we have discovered some of the biases and details that were previously hidden within V4-MiSeq analysis [42]. More generally, this research helps to address a gap that exists in the research of style tissue microbiomes. Specifically, 430 isolates were sequenced from the tips and the bases of *F. graminearum*-treated silks, in addition to the 367 tip/base isolates sequenced from healthy silks [70], from 14 maize genotypes belonging to diverse heterotic groups and spanning a variety of Gibberella ear rot (GER) resistance levels. The same silk samples were previously analyzed using V4-MiSeq (254 bp) [42], while this study used the longer-read 16S Sanger sequencing on individual isolates for a better taxonomic resolution and to match isolates to the previously defined MiSeq taxa. Furthermore, as a relatively uniform tissue, the silk offers a rare opportunity to study true spatial differences within the microbiome of an individual plant organ, which has seldom been investigated (e.g., Schlechter et al., 2019; Simmons et al., 2020). This library of microbes and the key lessons from this study may eventually lead to novel biocontrol or abiotic stress treatments for farmers, or tools to help maize breeders select for improved plant health, a beneficial silk microbiome, and disease resistance. In addition to *F. graminearum*, maize silks are impacted by other mycotoxigenic fungi and pathogens as well as by insects [3]. Drought, which prevents silk emergence and hence pollen capture, threatens reproduction and grain yield in maize and may worsen due to climate change [42,76]. Degrading and nitrogen-limited soils, including in Sub-Saharan Africa [77], similarly prevent silk emergence [78,79,80]. 

### 4.2. Effects of F. graminearum Treatment on the TSM in Comparison to Healthy Silks

The comparisons between the *F. graminearum*-treated TSM and the healthy TSM were based on one pooled replicate from the tip and the base samples of each of the 14 maize genotypes tested, from a single field season, so that consistency and the presence of some strains could be assessed, but differences should be investigated with more replicates and years before making confident conclusions. 

Within these study limitations, overall, an increase in the number of isolates and a collapse in the bacterial diversity across taxonomic levels was associated with the *F. graminearum* treatments in comparison to the healthy silks. This echoes the results from Khalaf et al. [42], which observed an increase in total 16S read counts and a collapse in diversity in the *F. graminearum*-treated silks. Other studies have made similar conclusions, with a collapse of microbial diversity accompanying *Fusarium* infection. For example, Bakker and McCormick [81] analyzed the microbiome of wheat kernels in relation to the *F. graminearum* infection in conditions that were conducive to disease, and found that bacterial and fungal diversity were negatively correlated with disease severity. Additionally, a study by Zhou et al. [82] showed a negative correlation between *Fusarium* wilt disease incidence and the diversity of bacteria and fungi in the tomato rhizosphere. It was previously suggested that the taxa which increased in abundance upon the *F. graminearum* infection may be part of a response against the fungus [42]. Similar to what was observed in the healthy silks [70], *Pantoea agglomerans* and *Pantoea ananatis* were found to contain high strain-level diversity that persisted in the *F. graminearum*-infected transmitting silks (27 and 25 OTUs, respectively) (Figure S9). Perhaps some of the bacteria which remain and multiply during an *F. graminearum* infection are defending the host plant’s reproduction to ensure their own survival, whether on the pollen or on/in the transmitting silks [70]. Interesting changes were observed in the taxonomies of transmitting-silk isolates upon the *F. graminearum* infection at different taxonomic levels. Many taxa increased upon the *F. graminearum* treatment, as observed at the level of phyla, class, family and genus, and some genera were seen to decrease upon the *F. graminearum* treatment; details are expanded upon in Supplementary Text S2.

#### Species Level Differences

Five species (*Enterobacter asburiae*, *Pantoea eucalypti*, *Pseudomonas parafulva*, *Pseudomonas rhodesiae* and *Sphingomonas parapaucimobilis*) were cultured from at least three genotypes in the *F. graminearum*-infected silks, but never from the healthy silks (Figure 6, purple and blue cells). There are some possible reasons why these species were not cultured from healthy silks: similar to the note above, they may have a low titre and were missed due to the limitations of culturing; or alternatively they could be truly absent in the healthy silks, and introduced via the environment or *F. graminearum*. Another study from our lab has shown the presence of 3 of these species in pollen from exotic maize (*E. asburiae*, *P. parafulva*, *S. parapaucimobilis*) suggesting they may have had a low titre here [51]. *E. asburiae* was previously identified as a maize seed endophyte [83], while *S. paucimobilis*, closely related to *S. parapaucimobilis*, has been identified as a rice root endophyte [84]. Bakker and McCormick [81] found that OTUs identified as *Sphingomonas* were negatively correlated with the pathogen load in *F. graminearum*-infected wheat kernels, and it was suggested that *Sphingomonas* may inhibit *Fusarium* or simply decline in abundance as *Fusarium* colonizes the grain. In the flowering tree *Mallotus japonicus*, compared to male flowers, female flower samples were shown to be more abundant in *Sphingomonas* ASVs [40]. Perhaps *Sphingomonas* is an important endophyte in the style tissues of many plants.

Likewise, some species were cultured more frequently in *F. graminearum*-infected silks than healthy silks, specifically *Delftia acidovorans*, *Klebsiella aerogenes*, *Klebsiella grimontii, Pantoea ananatis*, and *Stenotrophomonas pavanii*) (Figure 6). *Delftia* has been mentioned as a biocontrol agent above [85,86] and *Delftia acidovorans* specifically is a known endophyte [87]. A *Delftia* ASV was more abundant in the female flowers of *M. japonicus* [40], a plant that is susceptible to the pathogen *Erwinia amylovora,* which enters via the style and other reproductive tissues [88]. Though some strains of *P. ananatis* are maize pathogens, it has also been shown to promote maize growth [89]. *Pantoea ananatis* is present in maize seeds [83] and is the most prevalent and diverse species in maize pollen [51]. Especially relevant, *P. ananatis* has been shown to reduce the *F. graminearum*-derived mycotoxin deoxynivalenol in wheat [53]. *Stenotrophomonas pavanii* is an endophyte shown to combat *Fusarium oxysporum* in lupin roots [90].

Some species were cultured from both healthy and *F. graminearum*-treated silks, but were notably reduced in *F. graminearum*-treated silks, including *Lactococcus lactis* and *Microbacterium testaceum*. As mentioned above, they have potential to be endophytes but could provide other benefits to the host, aside from *F. graminearum*-defense. For example, *L. lactis* strains isolated from maize aerial roots have been shown to have robust biological nitrogen fixation activity [91,92].

### 4.3. Tip versus Base Location Effect on the Cultured TSM

In the MiSeq study by Khalaf et al. [42], the tip and the base silk samples could not be directly compared because different DNA kits and protocols were used for DNA isolation, and internal controls revealed differences in the quality of the results. In this current study, culturing allowed for some direct comparisons between the tip and the base tissues, albeit limited to a single field block and season. This was of interest, because *F. graminearum* was inoculated at silk tips which thus represented the first line of defense against this pathogen. Here, more isolates were cultured from the tip tissues (271 isolates) than the base tissues (127 isolates) in the *F. graminearum*-infected silks, based on filtered sequences (Figure 3). For example, *Klebsiella*, *Acinetobacter* and *Delftia* were cultured much more frequently from the *F. graminearum*-infected tip than from the base samples. *Delftia acidovorans* was cultured from the tip but never from the base. In addition to bacterial abundance, the tips of the *F. graminearum*-infected maize silks gave rise to greater taxonomic diversity. This was apparent across all taxonomic levels. At the OTU level, the *F. graminearum*-treated tips had 189 OTUs compared to 110 OTUs in the base. These results contrast the healthy silks in which the diversity was relatively evenly distributed between the tip (188 isolates, 177 OTUs) and the base (162 isolates, 165 OTUs), suggestive of a continuous tissue from a microbial perspective. Furthermore, isolates matching *F. graminearum*-induced and core V4-MiSeq taxa were more frequently found in the tip tissues (Figure 9 and Figure S10). 

This diverse, tip-heavy library cultured from the *F. graminearum*-treated silks may exist because these diverse strains provide a variety of benefits or advantages to the host plant, such as defending the susceptible entryway to the grain against pathogens/insects or providing protection against abiotic stress or UV radiation. Alternatively, the diversity of bacteria in the tips may simply be due to their proximity to the external environment during a susceptible interval, perhaps being introduced by or with *F. graminearum* which itself has a microbiome [93]. More studies are needed to verify whether the tip-associated bacteria were anti-fungal, or were simply taking advantage of the infected environment. However, these results suggest that upon *F. graminearum* infection, the silks may no longer represent a continuous tissue. Perhaps these natural biocontrol members or innate plant defenses [94] cordon the invading fungi and bacteria to the tip, or perhaps not enough time passed for them to colonize the base of the silks. 

Although some cultured OTUs were found more frequently in either the base or the tip samples, there was some consistency (Figure 7). In particular, the cultured OTU 375 (*Stenotrophomonas pavanii*) was rather consistent across the tip and the base samples, indicating that some resilient core taxa may be endemic across the full length of silks. OTU 375 is further discussed below.

### 4.4. Maize Genotype Effect on the Cultured TSM

In the V4-MiSeq study by Khalaf et al. [42], the effect of maize genotype on the TSM was not a focus. In this study, phyla-level bacterial diversity within a host genotype was found to be lower in the *F. graminearum*-treated silks than in the healthy silks. In the healthy silks, 9/14 host genotypes were each the source of bacterial cultures from four phyla [70], whereas in the *F. graminearum*-treated silks, only two genotypes possessed four cultured phyla, and only one genotype spanned three phyla (Figure 1b). This observation demonstrates that the overall collapse in TSM diversity after *F. graminearum* infection occurred within individual genotypes, and was not simply an average effect, as noted above.

Two genotypes (CO449 and CO432) of the Minnesota heterotic group contained the most diversity when infected with *F. graminearum.* This was in stark contrast to the findings from the healthy silk microbiome, in which Minnesota 13 heterotic group inbreds had low diversity overall [70]. CO449 is defined as moderately resistant-resistant for both silk and kernel Gibberella ear rot resistance, while CO432 is defined as having highly resistant silks and intermediate resistant kernels. Perhaps, upon *F. graminearum* infection, these maize genotypes upregulate bacteria which were previously present in trace amounts, or “import” more bacteria into the silks from the environment or the vascular system through the developing cob.

### 4.5. Cultured Core and V4-MiSeq Taxa Evaluations

#### 4.5.1. Defining the Cultured Core Transmitting Silk Microbiome at the OTU Level

The large culture collection and high taxonomic resolution permitted an opportunity to predict a cultured core transmitting silk microbiome within the limitations of the study. Out of 389 unique cultured OTUs (from healthy and *F. graminearum*-treated silks), there were 11 OTUs which were prevalent in both healthy and *F. graminearum*-treated silks, defined here as cultured from at least three host genotypes of each treatment. These conserved taxa were OTUs 117, 119, 130, 158, 271, 276, 290, 292, 372, 374 and 375 (Figure 8). Apart from cultured OTUs that were prevalent in both treatments, we were also interested in identifying OTUs that were prevalent specifically to the *F. graminearum*-treated silks, which could be used to develop microbial molecular markers to help breeders select potentially defensive silk microbes—increasing their abundance and stability and/or stacking them together in a single hybrid. In terms of cultured OTUs which were found in at least three host genotypes, the *F. graminearum*-treated silks had 48 compared to 26 in the healthy silks. The taxa that survived and thrived through the taxonomic collapse were also more prevalent: not only more abundant in numbers overall, but also across more genotypes, revealing a more consistent microbiome in maize silks after *F. graminearum* infection. Cultured OTU 375 and 291 marked the strongest members of the cultured core microbiome that could be identified for the *F. graminearum*-treated transmitting silks, having been found in 12 maize genotypes (Figure 7). 

Cultured OTU 375 (*Stenotrophomonas pavanii*) was the most prevalent cultured OTU, not only in the *F. graminearum*-treated silks but also in the healthy silks, but its prevalence increased from across six genotypes in the healthy silks to 12 genotypes in the *F. graminearum*-infected silks, where it was also the most frequently cultured. When combined, cultured OTU 375 appears to be the most conserved taxa in the cultured TSM, found in 13/14 maize genotypes. As mentioned above, *Stenotrophomonas* are known endophytes, many with anti-*Fusarium* traits [95,96,97,98,99,100] including against lupin root rot caused by *Fusarium oxysporum* [90]. These observations raise the possibility that cultured OTU 375 may have biocontrol or other beneficial properties in maize and should be investigated further.

Cultured OTU 291 (a taxon of *Pantoea anthophila* or *P. ananatis*) was tied with OTU 375 as the most prevalent cultured OTU in the *F. graminearum*-treated silks, and it was cultured from all of the heterotic groups. Moreover, cultured OTU 291 was frequently assigned to isolates which were identified as *P. ananatis* (covering 11 genotypes in Figure S9), although it also appeared in multiple isolates identified as *P. anthophila*, underscoring how genetically similar the 16S sequences are between *Pantoea* species. *Pantoea ananatis* may be a pathogen or endophyte, although *Pantoea* pathogens of maize are uncommon in Ontario [13]. “*Anthophila*” means “flower-loving,” and *P. anthophila* was named as such because strains have been isolated from flowers [101], so it is interesting that (1) this species was cultured from the transmitting style tissue of the female maize flower, and (2) a particular strain of this species was isolated across all of the heterotic groups of maize tested in this study. Perhaps this strain of *Pantoea* plays an important role in this flowering structure.

Isolates with long-read (V1-V9) 16S-sequences from the cultured bacteria were also compared to the short-read V4-MiSeq-defined core and the *F. graminearum*-indicator taxa. Matches were detected, which helped to improve the taxonomic resolution of the earlier predictions. Further details can be found in Supplementary Text S3.

#### 4.5.2. Evaluating Diversity within V4-MiSeq Taxa at the Cultured OTU Level

We hypothesize that the short 16S V4 sequences masked diversity within the TSM. Indeed here, for example, the *F. graminearum*-treated silks contained 46 cultured isolates that matched V4-MiSeq OTU 3 (core and consistent *F. graminearum*-indicator) which spanned 27 cultured OTUs (Figure 9 and  Figure S10). A single silk tip sample (from P9855HR) gave rise to 11 cultured OTUs matching V4-MiSeq OTU 2, another core and consistent V4-MiSeq *F. graminearum*-indicator. These results demonstrate the diversity of cultured OTUs within V4-MiSeq core taxa OTUs. Likewise, diversity within V4-MiSeq non-core *F. graminearum*-indicator taxa was observed via cultured OTUs.

Some individual V4-MiSeq OTUs matched many unique cultured OTUs, without unifying or consistent OTUs. Rather than individual strains or species of bacteria having core membership or increasing upon *F. graminearum*-infection, perhaps a broader taxonomic consortium is responsible, because the host plant or *F. graminearum* provide specific nutrient profiles or a habitat which supports related microbes.

#### 4.5.3. Conserved Cultures at the OTU Level within V4-MiSeq Taxa

When isolates which matched a V4-MiSeq prediction were cultured from multiple silk samples and belonged to the same cultured OTU, it suggested that a single strain may be responsible for the V4-MiSeq prediction. These isolates may be especially useful in basic and applied studies in the future. Examples include cultured OTU 317, which matched V4-MiSeq core OTU 28; cultured OTU 7, which matched V4-MiSeq core OTU 31; cultured OTU 117, which matched V4-MiSeq core OTU 3; cultured OTU 62, which matched V4-MiSeq *F. graminearum*-indicator OTU 8; cultured OTU 54, which matched V4-MiSeq *F. graminearum*-indicator OTU 9; and cultured OTUs 130 and 138, which matched V4-MiSeq *F. graminearum*-indicator OTU 17 (Figure 9 and Figure S10–S12). It is possible that there are additional individual strains that are conserved, but were missed here due to suboptimal culturing conditions for those microbes.

Khalaf et al. [42] highlighted that a single V4-MiSeq taxon (*Pantoea* OTU25) represented 15–25% of the healthy TSM, and was also prevalent in 99% of healthy and *F. graminearum*-treated silk samples. We wanted to discover the underlying strain, but discovered that multiple cultured OTUs matched V4-MiSeq OTU 25; these cultured OTUs appeared across many maize genotypes, most notably, cultured OTU 291 (*Pantoea anthophila* or *P. ananatis*) in 11 genotypes, cultured OTU 289 (*P. anthophila* or *P. ananatis*) in nine genotypes, cultured OTU 290 (*P. anthophila* or *P. ananatis*) in eight genotypes and cultured OTU 292 (*P. anthophila* or *P. ananatis*) in eight genotypes. This result suggests that V4-MiSeq OTU 25 may actually represent a narrow group of microbes which make up part of the TSM core. V4-MiSeq OTU 25 also increased in the base tissues after *F. graminearum* infection. 

As mentioned above, cultured OTU 375 appeared very consistently across silk samples. Cultured OTU 375 may represent an individual bacterial strain that is an important member of the core, as it matches V4-MiSeq core OTU 34.

#### 4.5.4. Overview of Cultured Bacteria Matching Non-Core V4-MiSeq *F. graminearum* Indicators

Interestingly, the healthy silks rarely gave rise to *F. graminearum*-indicators that were not also members of the V4-MiSeq predicted core (Figure S12). Specifically, nine of the 10 non-core V4-MiSeq *F. graminearum*-indicator taxa had many matching isolates cultured from *F. graminearum*-infected silks (57 isolates), while the healthy silks originated cultures from only two of the non-core *F. graminearum*-indicators (6 isolates). Of these two non-core *F. graminearum*-indicators, one was not found in the *F. graminearum*-treated silks (V4-MiSeq OTU 49), while the other (V4-MiSeq OTU 17) matched three cultured OTUs (OTU 130, OTU 138, and OTU 140), which were found in both the healthy and in the *F. graminearum*-treated silks, indicating some high resolution-level consistency within longer-read 16S sequences. The general absence of non-core, cultured *F. graminearum*-indicators in the healthy silks may be due to their low presence in these tissues, which would be consistent with the V4-MiSeq prediction wherein these OTUs increased in abundance with the *F. graminearum* infection and were prevalent across samples. These bacteria should be investigated for anti-*F. graminearum* potential.

#### 4.5.5. V4-MiSeq Taxa in the Context of Silk Tip and Base Locations

Cultures matching V4-MiSeq *F. graminearum*-indicators and core members were more frequently detected in the tip than the base tissues; this may be due to the larger number of isolates and OTUs that were found in the silk tips, as noted above. The *F. graminearum*-indicators may have been frequently found in the silk tips due to their association with *F. graminearum*, which was applied at the tips. As mentioned above, these microbes should be investigated further for plant defense potential.

In the *F. graminearum*-treated silks, the V4-MiSeq-predicted Base 2017 *F. graminearum*-indicator taxa were more widespread across host genotypes than the Tip 2017-specific *F. graminearum*-indicator taxa (Figure 9). It is possible that these Base *F. graminearum*-indicators may come from the host plant itself and play a key role in maize silks. The base of the silks is consistently close to developing seeds, and thus a location that one would assume should be protected from random colonization. Consistent with such a reproductive barrier, in the flowering tree *Mallotus japonicus*, the female flower, from which seeds and fruits develop, appeared to be more selective than male flowers in terms of accepting microbes from visitor insects [40]. 

### 4.6. Study Limitations and Future Experiments

This study faced limitations involving culturing (which can miss many bacterial species), sequencing (e.g., switching primers during the experimental pipeline) and the use of samples from a single field season and a single pooled replicate of each field block. In terms of the latter point, as a result, it is unknown whether the presence of individual strains may have been the result of host selectivity or random uptake from the environment.

There were additional challenges and limitations due to the *F. graminearum* treatments in this study. *Fusarium* infection can be inconsistent, and studies have previously faced challenges in achieving sufficient and consistent infection under experimental conditions (i.e., due to weather) [102,103,104], although this issue was mitigated by the application of three *F. graminearum* isolates and misting to encourage disease development.

Additionally, natural *Fusarium* infection is common, and infections can lack noticeable symptoms [29,105], meaning the adjacent healthy silk rows could have been unintentionally infected with wild *F. graminearum*, albeit with a much lower dose of inoculum than the artificially infected plants. A greater risk was transmission of *F. graminearum* inoculum between rows of maize via splashing from the misting system and rain, people and wild animals, although there were guard rows.

Although we could reveal diversity and consistency with respect to V4-MiSeq predictions, we could not make high-confidence comparisons about differences between the healthy and the *F. graminearum*-treated silk cultured microbiomes. This is due to the fact that many microbes would have been missed during culturing, and because the experiment was not designed to test this hypothesis, with only one replication of each pool. This allowed us to comment on the presence and consistency of some bacteria, but not confidently make statements about absences or differences. Additional replicates and years of data should be analyzed, as already noted, and/or a microbiome study should be undertaken involving newer full-length 16S next-generation sequencing (see below), before drawing such conclusions. Additionally, a microbiome analysis of the *F. graminearum* inoculum should also be conducted, though an attempt at this did not reveal the presence of bacteria (E. Khalaf and M.N. Raizada, unpublished); this result was contradicted by a later study [93]. Ali et al. [93] stated that in wheat, *F. graminearum* recruits endohyphal nitrogen-fixing bacteria from soil, which increases the pathogenicity of *F. graminearum*. These bacteria include *Stenotrophomonas pavanii* and *S. maltophilia,* which were cultured here; however, *S. pavanii* was abundant in both the *F. graminearum*-infected and the healthy silks [70], while *S. maltophilia* is also present in maize pollen [51] and in *Zea nicaraguensis* seeds [83]. Nevertheless, this observation indicates that the nitrogen-fixing bacteria identified in the *F. graminearum*-treated TSM should be carefully evaluated as to whether they may improve plant growth or aid *F. graminearum*.

A significant limitation of this study is that the origin of these bacteria is unclear. They could originate from pollen, silks, *F. graminearum* or simply enter the silks from the external environment. Studies focusing on the separated microbiomes of pollinated silks, pollen and unpollinated silks in more diverse landraces and related ancestors of maize are underway [106].

Another limitation of the study, by design, was that it did not address host impacts. It is unknown whether these bacteria are beneficial or pathogenic, whether they alleviate or worsen the effects of *F. graminearum* or if they are neutral [43]. These unknowns can be resolved with future experiments.

Moreover, some beneficial microbes may have been lost along the path of breeding modern varieties [107], so an interesting next step would be to culture microbes from pathogen-treated landraces and ancestors of maize to reintegrate them into modern breeding programs.

### 4.7. Lessons and Future Perspectives

The style tissue is critical for plant reproduction and disease, yet few studies have systematically cultured microbes from plant reproductive tissues, and these studies have typically been conducted on stigma or pollen [49,50,108]. This study suggests that the style, at least after pollination, contains significant bacterial diversity and is worth exploring in other plant species. More generally, the evidence suggests that pathogen treatment of a target tissue can result in a different diversity of culturable microbiomes compared to healthy tissue. Similar results have been observed in other studies [81,82]. What is more novel is that culturing bacteria at different distances from a pathogen inoculation site (i.e., silk tip versus base) can affect the diversity retrieved. This result warrants more trials. 

There has been a call for more research to better understand the interactions between *Fusarium* and crop microbiomes in order to manage disease and develop strategies including biological control [43], and the current research is a step towards a better understanding of not only *Fusarium*-host-microbiome interactions, but also the interpretation of next-generation sequencing results. Given the limitations shown here of short-read 16S sequencing (e.g., V4-MiSeq), future studies should use culture-independent, whole-length 16S sequencing (e.g., PacBio, Oxford Nanopore Technologies) [109] or the most up-to-date sequencing technology for a complete, systematic analysis.

Moving forward, there is interest in (living) biocontrol products, as well as applying their secondary metabolites to help prevent *Fusarium* crop diseases (either directly, or by inducing host resistance), although it is difficult to achieve consistency in field results [43,44]. Past attempts at finding biocontrol agents against *F. graminearum* have used microbes that were not isolated from the tissue targeted by the pathogen. Here, hundreds of bacterial strains were isolated and characterized that originated from pollinated silks: the tissue and developmental stage at which *F. graminearum* infects [9]. These bacterial strains thus provide a significant and novel resource for future biocontrol efforts against this pathogen and other pathogens that use the silk to infect maize grain, notably in Latin America and Africa, and deposit mycotoxins (including carcinogens) into grain, such as *Aspergillus flavus* Link, *Aspergillus parasiticus* Speare, *Fusarium verticillioides* (Sacc.) Nirenberg, *Ustilago maydis* (DC) Corda and *Stenocarpella maydis* (Berk.) Sutton [3]. The isolates which were matched to the V4-MiSeq core and *F. graminearum*-indicator OTUs constitute a resource for the future; cultured OTUs 375, 291, 117, 317, 7 and 62 are of special interest. Furthermore, the reproductive microbiome should be considered in the context of the many microbes co-existing in consortia. Microbial consortia and breeding for beneficial microbes are some of the most practical next areas of focus to enable crop improvement [43,56,57,60,64,110,111,112].

Crop breeders have traditionally used plant genome molecular markers linked to disease resistance for breeding. The results of this study may also enable breeders to use microbial marker-assisted selection to select for consortia of heritable anti-*F. graminearum* bacteria, by direct selection of 16S sequences of the promising taxa identified in this study. An earlier microbiome study [42] showed that certain bacterial taxa in silks were induced by *F. graminearum*, but their functions could not be validated, nor were DNA sequences available that would have been sufficient for breeders to use them as molecular markers for selection. Here, by culturing bacteria from the same tissue samples, undertaking longer-length 16S sequencing and matching isolates to the *Fusarium*-induced taxa, a list of candidate microbial molecular markers has now been identified that breeders can use to select a beneficial silk microbiome that combines potentially protective bacteria alongside plant genetic selection, including host compatibility alleles that promote these microbes.

## Data Availability

The 16S sequences corresponding to the strains in this study were deposited in the NCBI GenBank, and accession numbers can be found in Supplementary Table S1.

## Supplementary Materials

The following supporting information can be downloaded at: https://www.mdpi.com/article/10.3390/pathogens12111322/s1. Table S1: Spreadsheet of isolate details, accession numbers, and sequences; Supplementary Text S1: Further details for methods [113,114,115,116,117]; Supplementary Text S2: Taxa increasing and decreasing upon *F. graminearum* treatment [118,119,120,121,122,123,124,125,126,127,128,129,130,131,132,133,134,135,136,137,138,139,140,141,142,143,144,145,146,147,148,149,150,151,152,153,154,155,156,157,158,159,160,161,162,163,164,165,166]; Supplementary Text S3: Comparisons of cultured bacteria to V4-MiSeq-defined Core and *F. graminearum*-indicator taxa; Figure S1: Example microbes cultured from maize silks; Figure S2: Number of isolates belonging to the cultured *Fusarium graminearum*-infected transmitting silk microbiome at the class taxonomic level; Figure S3: Number of isolates belonging to the cultured *Fusarium graminearum*-infected transmitting silk microbiome at the order taxonomic level; Figure S4: Number of isolates belonging to the cultured *Fusarium graminearum*-infected transmitting silk microbiome at the family taxonomic level; Figure S5: Genus level taxonomic identification of the cultured transmitting silk microbiome from *Fusarium graminearum*-infected maize silks spanning diverse heterotic groups; Figure S6: Species level taxonomic identification of the cultured transmitting silk microbiome from *Fusarium graminearum*-infected maize silks spanning diverse heterotic groups; Figure S7: Number of isolates belonging to the cultured *Fusarium graminearum*-infected transmitting silk microbiome at the species taxonomic level; Figure S8: Comparison of number of isolates belonging to the cultured, healthy (non-infected) and *Fusarium graminearum*-infected transmitting silk microbiome at the family taxonomic level; Figure S9: Cultured OTUs identified within bacterial species in the cultured *Fusarium graminearum*-infected transmitting silk microbiome; Figure S10: Comparison of V4-MiSeq core OTUs to matching cultured OTUs identified from *Fusarium graminearum*-treated transmitting silks of maize; Figure S11: Comparison of V4-MiSeq core OTUs to matching cultured OTUs identified from healthy transmitting silks of maize; Figure S12: Comparison of *Fusarium graminearum*-induced V4-MiSeq OTUs (*F. graminearum*-indicators) to matching cultured OTUs isolated from untreated (healthy) transmitting silks of maize.
