# Supplementary material for: The Cultured Microbiome of Pollinated Maize Silks Shifts after Infection with Fusarium graminearum and Varies by Distance from the Site of Pathogen Inoculation
Source: Pathogens. 2023 Nov 6;12(11):1322. doi: 10.3390/pathogens12111322 (PMC10675081; doi:10.3390/pathogens12111322)
Supplement: Supplementary file 1 [file pathogens-12-01322-s001.zip › pathogens-2672130-supplementary.pdf]

Supplemental Materials

Supplementary Text S1: Further details for methods

S1.1. Growing plants, harvesting, and storing silks

The silks used in this study were from the same tissue pools described earlier [42]. Briefly, in a blind experiment, a selection of 14 maize genotypes (11 Agriculture and Agri-Food Canada inbred lines and 3 commercial Pioneer hybrids, Table 1 adapted from Khalaf et al. [42]) were grown in a field at the Ridgetown Campus of the University of Guelph, in Ridgetown, Ontario, Canada in 2017, as previously described [42]. These maize genotypes vary in resistance to *F. graminearum* and belong to heterotic groups including BSSS, Minnesota 13, E. Flint, P3990, Iodent, E. Butler, and Lancaster (Table 1). The maize lines were grown in a randomized, split-block design with guard rows (Hybrid MZ540, MaizeX, Canada) around each of the 3 blocks. Each block contained 2 rows, each being 2 m long and an independently randomized row of the 14 maize genotypes. Randomized across the 2 rows, 1 plot (up to 16 plants) of each genotype was treated with *F. graminearum*, while the other plot of the same genotype was left untreated. At silking stage, the plants were allowed to open-pollinate (no tassel bags or shoot bags were used). Plots were overhead misted before and after *Fusarium* inoculation until harvest as previously described [42] to promote disease progression. From each plot, 3 average-sized cobs were harvested on August 21, 2017. Cobs were transported to the University of Guelph main campus, Guelph, Ontario, Canada, where, under sterile conditions, the environmentally exposed silk tissues were first removed, followed by husk leaves, exposing the underlying silks. The exposed cobs were placed on sterile 150 mm diameter Petri dishes under sterile conditions. The silks were divided into equal thirds using sterile razor blades, from which the tip and base samples were separately taken (middle excluded) (Figure S1). Silk tips or base tissues were pooled from the 3 plants selected randomly from the field plots, and then a subsample of 10 silk tips or base tissues per pool were selected randomly for microbial isolation from block 1 only; some samples had fewer than 10 silks. These silks were frozen in sterile 40% glycerol in Parafilm-sealed Petri dishes at -80 ˚C on the day of harvest. In total, bacteria in this study were cultured from 42 plants (3 plants/plot x 1 plot per genotype x 14 genotypes) separately cultured from the silk base and tip for a total of 28 pools representing 84 individual tissue samples.

S1.2. Culturing Microbes

To culture the microbes, the silks were allowed to thaw completely before adding 600 μL of autoclaved 0.05 M sodium phosphate buffer (PBS: 14.425 mL of 1 M Na_2HPO_4, and 10.575 mL of 1 M NaH_2PO_4, in a final volume of 500 mL of autoclaved ddH_2O, at pH 7) to each original Petri dish. The thawed silks were swirled around with a pipette tip. A pipette was used to transfer the silks and 300 μL of the liquid mixture from the plate into a sterile mortar, where the silks were ground with a sterile pestle. Samples were diluted with sterile sodium phosphate buffer, and 100 μL aliquots were plated onto 15 mL LB agar plates (pH 7.2) and 15 mL potato dextrose agar (PDA) plates (pH 5.6). Control plates of PDA and LB agar were plated with the PBS which was used to make dilutions to check for background microbial contamination. Tip samples were plated as full concentration, 1/100 dilution, 1/1000 dilution, while base samples were plated as full concentration and 1/100 dilution due to a lower number of colony forming units (CFUs). The plates were incubated without parafilm for 3 days at 30˚C, and unique colonies were restreaked onto new agar plates, matching the media from which they came. The unique colonies were marked with a permanent marker on the back of the Petri dish. The original plates were returned to the 30˚C incubator for 2 additional days (total 5 days in incubator) to observe any new colonies (slow growers) or if 2 colonies that previously looked the same grew to appear different from one another. These new colonies were then restreaked onto fresh agar and incubated at 30˚C. A single colony from each restreak plate was transferred into a 3 mL liquid culture. Plates were sealed with Parafilm and

stored at 4˚C. The liquid cultures were used to make pairs of long-term storage cultures as 1 mL frozen glycerol stocks (400 μL liquid culture, 600 μL sterile 40% glycerol) in sterile screw-cap cryovials. In cases where microbes failed to grow in liquid culture, glycerol stocks were made by scraping the colonies from the agar plate and placing them into tubes containing 600 μL sterile 40% glycerol and 400 μL sterile liquid media (LB or PDA, according to the original media). The remaining liquid cultures were used to isolate bacterial genomic DNA for taxonomic analysis. When necessary, fresh liquid cultures were prepared for DNA isolation.

S1.3. Bacterial DNA Isolation, Polymerase Chain Reaction, and Amplicon Purification

DNA was isolated from liquid cultures of bacteria using the Qiagen QIAamp DNA mini kit (cat# 51306) (Figure S1). Subsequently, 2 μL of the isolated genomic DNA were quantified using a Qubit v1.2 fluorometer (#Q32857, Molecular Probes, Invitrogen by Life Technologies, United States) with two standards. Polymerase chain reaction (PCR) amplification of the V5-V9 regions of 16S rDNA was originally done using the primer set 799F [5′-AACMGGATTAGATACCCKG-3′] and 1492R [5′-GGTTACCTTGTTACGACTT-3′] in a PTC200 DNA Thermal Cycler (MJ Scientific, USA). Each PCR mixture contained approximately 100 ng of genomic DNA, 20 μL of GoTaq Green Master Mix (M712C, Promega, United States), 2 μL of each primer (10 μM working stock), and nuclease-free H_2O to a final volume of 40 μL. The PCR conditions were as follows: an initial denaturation at 94°C for 7 min, then 35 amplification cycles at 94°C for 45 sec, 48°C for 1 min, 72°C for 2 min, and a final extension at 72°C for 7 min [113]. PCR products were run in a 1.5% agarose gel for approximately 45 min, and bands of ~700 bp were visualized using a Gel Doc XR+ System (Universal Hood II, Bio-Rad, USA).

As the study progressed, the primer set was changed to encompass nearly the entire 16S rDNA gene (V1-V9) using the primer set, 27F [5′-AGAGTTTGATCMTGGCTCAG-3′] and 1492R [5′-GGTTACCTTGTTACGACTT-3′]. The reasoning behind this was to allow for direct comparison between the sequences from these cultured bacterial isolates and the 16S MiSeq sequences (V4 region, ~254 bp) generated from the same silk pools as the prior study [42], as well as to provide better taxonomic predictions (~700 base pairs vs. ~1400 base pairs, respectively). The PCR conditions were as follows: an initial denaturation at 96°C for 3 min, followed by 35 amplification cycles at 94°C for 30 sec, 48°C for 30 sec, 72°C for 90 sec, then a final extension at 72°C for 7 min [114]. The PCR amplicon size of ~1400 bp was confirmed via using gel electrophoresis or capillary electrophoresis (QIAxcel Advanced Instrument, QIAGEN, Germany). Every batch of PCR reactions had a negative control in which genomic DNA was omitted. Successful PCR amplicons were purified from liquid using the Illustra GFX DNA and Gel Band Purification Kit (#28-9034, GE Healthcare, United States). Purified samples were quantified using a Qubit v1.2 fluorometer (#Q32857, Molecular Probes, Invitrogen by Life Technologies, United States) with two standards. Purified amplicons were stored at -20°C.

S1.4. Sequencing and Sequence Processing

Purified amplicons were run in BigDye Terminator v3.1 Labeling Reactions (Applied Biosystems, USA). Each sample had two reactions, one for each of the forward and reverse primers. For each reaction, 28 ng per 1 kb of template DNA (19.40 ng for primer set 799F/1492R and 41.02 ng for primer set 27F/1492R) and 10 pmol of primer were dried in PCR tubes, strips, or 96-well plates on an open thermocycler at 96 °C for 10 min. A master mix of BigDye:buffer:water 1:2:9 was mixed, and 12 μL was added to each reaction vessel. The 96-well plates were sealed with ThermaSeal film, gently vortexed, and briefly centrifuged before thermocycling using the following conditions: an initial denaturation at 96°C for 2 min, followed by 30 amplification cycles at 96°C for 30 sec, 48 °C for 15 sec, 60°C for 4 min, followed by soaking at 10°C. Samples were then submitted to the AAC Genomics Facility (University of Guelph) where they were sequenced using a 3730 DNA analyzer (Applied Biosystems, USA). The forward and

reverse sequences were trimmed and used to create a contig in BioEdit software [115] version 7.0.5.3 (10/28/05). However, samples with no contig, and/or only one sequence (forward or reverse) were not discarded from the bioinformatics pipeline. Sequences were deposited in the NCBI GenBank (Accession numbers available in Supplemental Information).

S1.5. 16S Taxonomic Predictions

For both primer sets, Basic Local Alignment Search Tool Nucleotide (BLASTn) searches were performed to obtain taxonomic predictions based on the best matches to the 16S ribosomal RNA sequence (bacteria and archaea) database at NCBI. The top hit for genus and species were recorded in the "moderate stringency genus" and "moderate stringency species" columns, respectively. If there were 2 top matches with the same Max Score and %identity values, both were included in the column. Amongst the best matches, filtering criteria were applied to ensure quality control. For genus level assignments, the genus was also placed in the "high stringency genus prediction " column if it met either of the following criteria: (1) the top hit had a 100% identity match; or (2) alternatively, the top hit had at least 5 more base pair matches than the next best genus predicted, and additionally (2a) had at least a 98.5% identity match or (2b) that the genus was consistent amongst the top 20 results. If these conditions were not met, a zero was placed in the "high stringency genus prediction" column. For species level assignments, the species was also placed in the "high stringency species prediction" column if it met either of the following criteria: (1) the top hit had a 100% identity match; or (2) the corresponding genus was in the "high stringency genus prediction " column, and additionally, (2a) the top hit had a 98% identity score and at least 2 more base pair matches than the next species predicted, or (2b) the top hit had a 99% identity score and at least 1 more base pair match than the next species predicted.

For *Curtobacterium,* further investigation was needed to determine the identity. For NCBI BLAST results labelled [*Curtobacterium*] *plantarum,* the square brackets around the genus indicated that it was in the process of being changed to a different genus; in this case the strain was reassigned to *Pantoea agglomerans*. The full result on NCBI was "*[Curtobacterium] plantarum* strain CL63 16S ribosomal RNA, partial sequence", and according to the American Type Culture Collection, this strain belongs to *P. agglomerans* (ATCC: 49174).

S1.6. Assigning Cultured Microbes to OTUs

16S sequence comparisons amongst the cultured microbes were used to determine which samples were unique and which were conserved and thus likely to belong to the same species/strain. Sequences were analyzed using FASTA data input into BioEdit Software [115] and compared using the Clustal-W alignment tool and BLASTn. Isolates were binned into unique OTUs. Isolates shared an OTU unless: (1) they had a unique single base insertion or deletion compared to each other, or (2) they had a single base pair difference compared to one another (excluding an "N" in either sequence, or a mismatch located adjacent to an "N" in either sequence, or a mismatch located within 20 bases from the end of a sequence after trimming excess "N's"). The cultured OTUs were also individually compared against a compiled FASTA file of the entire list of cultured OTUs from the study using BLASTn to ensure there were no redundancies. Again, samples with no contig and/or only one sequence (forward or reverse) were retained for subsequent analyses, but low-quality sequences were omitted. Some sequences were assigned to multiple cultured OTUs, possibly because they were short or had "N's" in key locations.

The cultured OTU sequences were of varying lengths and quality. To enable subsequent comparative analysis, sometimes contigs were created from the best sequences amongst isolates sharing the same OTU. For further analyses, isolate sequences and cultured OTU's were only kept if they were ≥500 bp and the percentage of "N's" in a sequence was ≤5%. Sample sequences and cultured OTU's that

had no contig and the combined length of their forward and reverse sequences added up to ≥500 bp were also kept for analyses.

S1.7. Construction of Phylogenetic Trees

S1.7.1. Simple tree

Samples were assigned at the levels of phylum, class, order, and family (Figure 1B). Samples with multiple, equal first matches from NCBI were only assigned to the taxonomic level which were shared by the equal first matches (i.e., A sample with the first matches *Rahnella variigena* and *Raoultella terrigena* would receive the assignments: phylum= Pseudomonadota, class= Gammaproteobacteria, order= Enterobacterales, family=unassigned. Family here would be unassigned because *Rahnella* and *Raoultella* belong to different families.).

S1.7.2. Detailed tree

The sequences of cultured strains were aligned with MEGA 11 software [116] using align by MUSCLE, and a maximum likelihood (ML) phylogenetic tree in circle-format was generated using the default parameters. The phylogenetic tree was exported in Newick format and illustrated in The Interactive Tree of Life online tool version 6.8 [117].

S1.8. Matching 16S sequences from cultured microbes to prior V4-MiSeq data

This study also involved comparing 16S data from microbes cultured from transmitting silks to Illumina V4-MiSeq data from the same tissues generated in an earlier study [42]. For this comparison, only cultured microbial sequences generated using the primer set 27F and 1492R could be compared to the earlier defined "core" transmitting silk microbiome [42]. The V4-MiSeq core OTUs in Khalaf et al. included taxa which were detected in at least 50% of samples and had a ≥1% relative abundance, taxa which had a ≥1% relative abundance but were less prevalent, and also taxa with a <1% relative abundance, together representing the most prevalent and dominant taxa [42]. Most V4-MiSeq core OTUs were detected across tip and base tissues in the MiSeq results. Matches with ≥96.06 % identity to core silk V4-MiSeq-amplicon sequence variants (V4-MiSeq-ASVs) with only N mismatches (or a single gap directly adjacent to multiple N's) were matched to a V4-MiSeq-ASV. When a sequence had multiple matches, the V4-MiSeq OTUs with the higher percent match was assigned to the isolate. To differentiate the cultured data from the MiSeq data, the OTUs were referred to as cultured OTUs and V4-MiSeq OTUs, respectively.

Supplementary Text S2: Taxa increasing and decreasing upon *F. graminearum* treatment

S2.1. Taxa increasing upon *F. graminearum* treatment - Phyla and class level

Compared to healthy silks, there was a distinct shift in *F. graminearum*-treated silks towards cultured Pseudomonadota (Proteobacteria), and specifically more Gammaproteobacteria (Figure 1B; Figure S2). Pseudomonadota and Gammaproteobacteria were also common in the cultured isolates from healthy silks, and in both treatments analyzed using V4-MiSeq [42,70]. Wheat is commonly infected by *Fusarium* sp., and wheat has been shown to have observed an abundance of Pseudomonadota [118]. The above cited study by Bakker and McCormick [81] showed the bacteria taxonomy of wheat heads to be dominated by Pseudomonadota (largely gammaproteobacterial). Gammaproteobacteria are known to live in a wide variety of environments, and at times in extreme conditions [119,120]. Perhaps *F. graminearum* infection represents a stressor that many Gammaproteobacteria are well-adapted to survive.

S2.2. Taxa increasing upon *F. graminearum* treatment - Family level

Bacteria of the family *Oxalobacteraceae* were cultured from *F. graminearum*-treated silks but not healthy silks (Figure S4). Perhaps some of the *Oxalobacteraceae* in *F. graminearum*-treated silks are anti-*Fusarium*. Members of the *Oxalobacteraceae* family (*Janthinobacterium* and *Duganella* spp.) are well-known antifungal taxa which encode secondary metabolites and chitinases [121]. The microbiomes of *Fusarium*-infected and control cucumber were previously shown to contain predominantly Pseudomonadota, and at the family level, *Oxalobacteraceae*, followed by *Rhodanobacteraceae [122]* – the composition also found in *F. graminearum*-treated silks but not in healthy silks. A member of the *Rhodanobacteraceae* family, namely *Rhodanobacter ginsengiterrae*, has been found to be antagonistic to *Fusarium solani* [123], which indicates a potential for the *Rhodanobacteraceae* found in *F. graminearum*-treated silks in this study to be anti-*Fusarium*. However, some *Oxalobacteraceae* species have also been found to grow inside of endophytic Ascomycota, termed endohyphal bacteria [124], raising the question of whether the *Oxalobacteraceae* bacteria in the *F. graminearum*-treated TSM could have been introduced via *F. graminearum*. Alternatively, the presence of *Oxalobacteraceae* may only be indirectly related to *Fusarium*. *Oxalobacteraceae* are well-known plant root- and rhizosphere-colonizers [125], and have been shown to be recruited by maize roots under nitrogen stress where they stimulate lateral root formation to improve the uptake of nitrogen [126], suggestive of a host growth promotion effect (e.g., auxin secretion).

Some bacterial families were cultured in both healthy and *F. graminearum*-treated silks, but were found much more frequently in *F. graminearum*-treated silks, namely *Enterobacteriaceae, Erwiniaceae, Xanthomonadaceae, Comamonadaceae*, and *Pseudomonadaceae. Enterobacteriaceae* have been associated with specific cultivars of cucumber which are resistant to *Fusarium oxysporum*, with many cultured strains showing antagonism [122]. In a study investigating the endospheric microbiome of banana, *Enterobacteriaceae* were detected in all samples, and potted experiments suggested that 4 of the strains that were inoculated improved resistance to Fusarium wilt disease [127]. Male *Mallotus japonicus* flower samples have been shown to be dominated by *Erwiniaceae* and *Enterobacteriaceae [40]*. In a study investigating detached wheat spikes which were inoculated with *F. graminearum*, 11 of the 14 most promising anti-*F. graminearum* isolates belonged to the family *Erwiniaceae* [53].

The literature shows a pattern of *Xanthomonadaceae* increasing upon *Fusarium* infection. In a previous study, *Xanthomonadaceae* was found in greater abundance in the rhizosphere of tomato that had Fusarium wilt disease [82]. Some of the most highly connected OTUs in *Arabidopsis thaliana* infected with *Fusarium oxysporum* belonged to the family *Xanthomonadaceae.* Additionally, soil microbiomes associated with Fusarium wilt have been shown to have higher abundances of *Xanthomonadaceae* [128]. Some members of *Xanthomonadaceae* are known phytopathogens, and it has been posited that they can worsen the impacts of Fusarium wilt disease [128]. However, *Xanthomonadaceae* were found most abundantly when banana plants were treated with anti-*Fusarium* bacterium *Bacillus velezensis* HN03, healthy soil, and

Fusarium oxysporum, and decreased significantly when treated with only healthy soil and *Fusarium oxysporum* [129], which may reveal the potential for *Xanthomonadaceae* to be working alongside *Fusarium*-suppressive bacteria. When looking at the literature in relation to maize, *Xanthomonadaceae* have been identified as keystone taxa in maize and faba bean intercropping [130]. In another study on maize stalks, a bacterial OTU had negative correlations with *Fusarium* OTUs, and also had negative correlations with the *Xanthomonadaceae* family [131].

Generally, *Comamonadaceae* have been associated with healthy soil microbiomes [128]. Both *Fusarium* spp. and bacteria in the family *Comamonadaceae* produce SCOOP-like (Serine-rich endogenous peptide-like) peptides which modulate plant immunity [132], which is interesting because *Comamonadaceae* isolates were more frequently cultured in *F. graminearum*-treated silks than healthy silks. By contrast, in a previous study, *Comamonadaceae* were more commonly found in healthy tomato plants, rather than tomatoes with Fusarium wilt disease [82]. Cucumber, which is also susceptible to Fusarium wilt disease, is thought to recruit *Comamonadaceae* endophytes via secretion of organic acids [133]. The *Comamonadaceae* and *Pseudomonadaceae* families are thought to include many potential endophytes with biocontrol activity [134], and in this study, they were more frequently cultured in *F. graminearum*-treated silks than healthy silks. Members of the family *Pseudomonadaceae*, including the genus *Pseudomonas* cultured from crops (which was cultured more frequently in the *F. graminearum*-treated maize in the current study), have long been thought to be a good source for biocontrol agents to prevent *Fusarium* diseases [52,135–137]. Indigenous soil *Pseudomonas* are also important in regards to suppression of Fusarium wilt disease in banana [138]. Of particular relevance, a strain of *Pseudomonas* isolated from wheat heads has been shown to be an effective biocontrol for Fusarium head blight [139].

S2.3. Taxa increasing upon *F. graminearum* treatment - Genus level

At the genus level, in addition to *Pseudomonas,* other genera which were cultured more frequently from *F. graminearum*-treated silks in this study include *Delftia, Klebsiella, Pantoea,* and *Stenotrophomonas* (Figure 4).

A *Delftia* strain has been previously identified as a potential biocontrol agent against many plant pathogens, including *Fusarium oxysporum* [85], and a bacterium of *Delftia/Comamonas* group taxonomy has previously been shown to break down fumonisin B1 [86], indicating the potential of the *F. graminearum*-associated *Delftia* in the current study to be anti-*Fusarium*.

Some members of the genus *Klebsiella* are maize pathogens [140,141], while others have beneficial relationships with maize and other crops [142–144]. *Klebsiella* are known to fix nitrogen and show evidence of being transmitted by maize seeds [145–148]. In Thompson et al. [70], some of the *Klebsiella* isolates from healthy maize silks were indeed shown to grow without nitrogen and possessed nitrogen fixation genes, indicating the possibility that these bacteria may be contributing nitrogen to silks, pollen, or pollen tubes. Therefore, the *Klebsiella* found in *F. graminearum*-treated silks may be beneficial, harmful, neutral, or a combination of the three.

As mentioned in Thompson et al. [70], the genus *Pantoea* contains some maize pathogens including *P. ananatis* [149–154], *P. agglomerans* [155], and *P. stewartii* [156], although the literature does not mention silks as a route of entry for these pathogens, and they are not common maize pathogens in Ontario. These three *Pantoea* species are also known to be endophytes in maize or grasses [89,157–159], leaving open the possibility that the *Pantoea* present in *F. graminearum*-treated silks could be beneficial to the host (e.g., anti-fungal).

Stenotrophomonas are known to act as endophytes of maize or grasses [95,96]. *Stenotrophomonas* has been previously shown to be an effective biocontrol agent against *F. graminearum* in wheat [97]. Another strain of *Stenotrophomonas* has been shown to act as a strong beneficial bacterium in wheat (plant growth and defense), but only in the presence of *F. graminearum,* suggesting that crops may recruit *Stenotrophomonas* upon infection [98]. Beneficial strains of *Stenotrophomonas* have been isolated from the

maize rhizosphere, including some with antagonism against *Fusarium* [99,100]. Combined, these observations lend support for the hypothesis that the *Stenotrophomonas* that increase in relative abundance in *F. graminearum*-infected maize silks have anti-*Fusarium* activity.

S2.4. Taxa decreasing upon *F. graminearum* treatment - Genus level

Some genera were cultured from both healthy and *F. graminearum*-treated silks, but were notably reduced in *F. graminearum*-treated silks, including *Chryseobacterium, Exiguobacterium, Lactococcus,* and *Microbacterium* (Figure 4). However, lactic acid bacteria, such as *Lactococcus,* are of interest for decontaminating *Fusarium* and mycotoxins in grain [160]. *Lactococcus* species have also been observed to decrease the levels of fumonisin B2 and roquefortine C in maize silage, although fusaric acid increased [161]. *Lactococcus lactis* from diseased date palm have been observed to have antifungal activity against *Fusarium* [162]. More generally, *Lactococcus, Chryseobacterium,* and *Microbacterium* have been shown to be endophytes in maize [163–165], and *Exiguobacterium* is a known endophyte of wheat [166]. In the current study, these genera may have appeared less frequently simply due to a low titre and culturing limitations, or because they were incompatible with *Fusarium* infection or may provide other benefits to maize, aside from defense against *F. graminearum*.

Supplementary Text S3: Comparisons of cultured bacteria to V4-MiSeq-defined Core and *F. graminearum*-indicator taxa

S3.1. Comparisons of cultured bacteria to V4-MiSeq-defined Core and F. graminearum-indicator taxa

Previously, 16S V4-MiSeq results (~254 bp) from the same silk samples were used to define the transmitting silk microbiome, including core taxa and *F. graminearum*-induced taxa (*F. graminearum*-indicators) [42]. Some V4-MiSeq taxa were members of both the core and *F. graminearum*-indicator groups. Here we used the longer 16S reads of the culture collection to improve the taxonomic resolution of the culture-independent TSM. Midway through this study, 16S primers for individual isolate PCR and Sanger sequencing were changed from 799F/1492R to 27F/1492R so that the longer reads overlapped with the V4 region and could be directly aligned to the V4-MiSeq results, and thus directly compared. Both healthy silks and *F. graminearum*-treated silks contained cultured isolates matching many V4-MiSeq predicted core taxa (Figure S10; Figure S11), supporting the prediction that transmitting silks have a set of conserved taxa.

S3.2. Clarifying taxonomic predictions

The taxonomic resolution of both the V4-MiSeq *F. graminearum*-indicator taxa and core taxa, which were previously assigned to the genus or family level, were improved in this study due to the long-read 16S sequences of cultured isolates. For example, V4-MiSeq OTU 31 was previously defined by V4-MiSeq as *Acinetobacter*, but here matched cultured sequences belonging to *Acinetobacter oleivorans* (2 from healthy silks, 4 from *F. graminearum*-treated silks) (Figure S10; Figure S11). Most notably, V4-MiSeq *F. graminearum*-indicators which were not members of the core received updated taxonomic predictions, thanks to the cultured isolates from *F. graminearum*-treated silks, as most were absent from healthy silk cultures (Figure 8). For example, V4-MiSeq *F. graminearum*-indicator OTU 9 was previously identified by V4-MiSeq as belonging to the *Burkholderiaceae* family, but 9 isolates from *F. graminearum*-treated silks matched this OTU and were identified more specifically as *Comamonas sediminis*.

Cultures from *F. graminearum*-treated silks also expanded or broadened some taxonomic predictions in comparison to the healthy silk results. In healthy silks, 8 isolates matching V4-MiSeq OTU 2 were cultured, and all were identified as *Atlantibacter hermannii*. However, in *F. graminearum*-treated silks, isolates matching V4-MiSeq OTU 2 were identified as *Atlantibacter hermannii* and *Kosakonia cowanii*, indicating that V4-MiSeq OTU 2 may have a somewhat broader taxonomic prediction than previously observed in Thompson et al. [70].

In some cases, the broad taxonomic level prediction of V4-MiSeq was revealed to be accurate by the longer-read 16S matches from cultures. For example, V4-MiSeq OTU 25 was previously predicted as *Pantoea*, and here, cultured isolates revealed a variety of taxa from the genus *Pantoea*. This example shows that rather than representing an individual bacterial strain, V4-MiSeq OTU 25 likely represents a group of *Pantoea* that are part of the core TSM and increase upon *F. graminearum*-infection.

V4-MiSeq OTU 6 (core and base 2017 *F. graminearum*-indicator) was cultured from neither healthy nor *F. graminearum*-treated silks in this study. V4-MiSeq was only identified it to the family level, Enterobacteriaceae, making it difficult to explain why this indicator was not recovered in culturing. Perhaps it was incompatible with the culturing conditions.

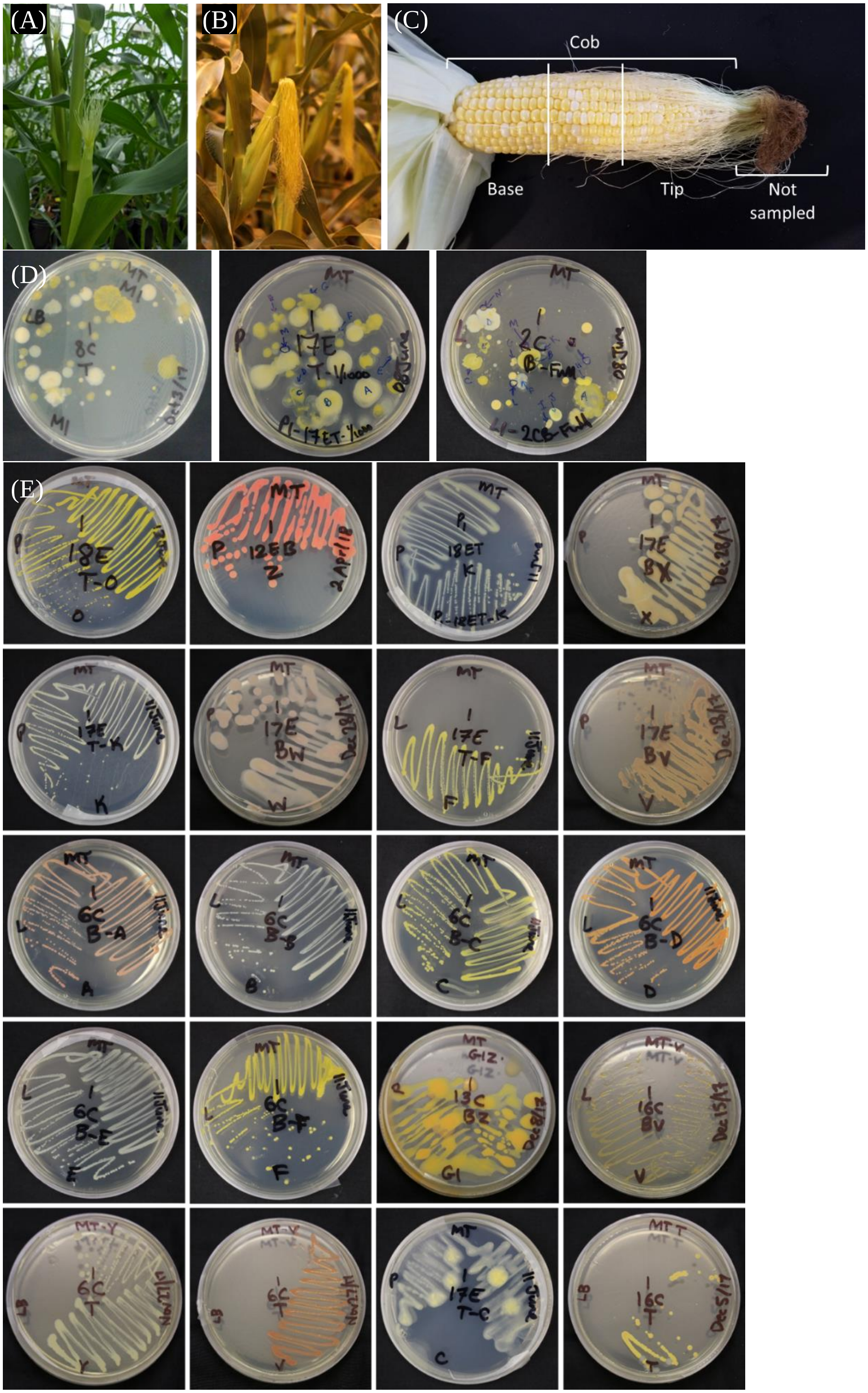
(A)
(B)
(C)
Cob
Base
Tip
Not sampled
(D)
(E)

Figure S1. Example microbes cultured from maize silks. **(a)** Photo of young silks. **(b)** Photos of extended silks. **(c)** Transmitting silk samples were taken from the tip and the base of each cob within the portion covered by husk leaves. **(d)** Photos showing examples of original culture plates from open-pollinated maize silks. **(e)** Photos showing examples of the microbial diversity cultured from open-pollinated maize silks.

Heterotic Group		European Flint	Early Butler	BSSS	BSSS/Minnesota 13	Minnesota 13	Minnesota 13	Minnesota 13	P3990	P3990/Iodent	Iodent	Lancaster	Pioneer Hybrid	Pioneer Hybrid	Pioneer Hybrid				
Maize Inbred/ Hybrid Line / Bacterial Class	Tip/Base	CO444	CO325	CO452	CO462	CO449	CO433	CO432	CO430	CO448	CO431	CO441	P35837	P38157	P9855HR	Total isolates	Total isolates, merged Tip and Base	Prevalence	Prevalance, merged Tip and Base
Actinomycetia	T	0	0	0	0	1	0	1	2	0	0	0	0	0	0	4		3	
	B	0	0	1	0	0	0	1	1	0	0	0	0	0	0	3	7	3	4
Alphaproteobacteria	T	1	0	0	3	3	0	4	2	0	1	1	1	0	0	16		8	
	B	0	0	0	0	0	0	2	1	1	3	0	0	1	0	8	24	5	10
Bacilli	T	0	0	0	0	2	1	1	0	0	0	0	0	0	3	7		4	
	B	0	0	0	0	1	0	1	0	0	0	0	2	0	0	4	11	3	5
Betaproteobacteria	T	6	0	3	3	6	0	1	0	4	0	2	1	0	0	26		8	
	B	0	0	1	0	1	0	1	0	0	0	0	0	0	0	3	29	3	8
Cytophagia	T	0	0	0	0	0	0	0	0	0	0	0	0	0	0	0		0	
	B	0	0	0	0	0	0	0	0	0	0	0	0	0	0	0	0	0	0
Flavobacteriia	T	0	0	0	1	0	0	0	0	0	2	0	0	0	1	4		3	
	B	0	0	0	0	0	0	1	0	0	2	0	0	0	0	3	7	2	4
Gammaproteobacteria	T	18	14	17	18	15	20	10	14	8	12	9	22	14	21	212		14	
	B	11	2	3	5	14	4	6	10	4	13	4	8	7	14	105	317	14	14
Sphingobacteriia	T	0	0	0	0	1	0	0	0	0	0	0	0	0	0	1		1	
	B	0	0	0	0	1	0	0	0	0	0	0	0	0	0	1	2	1	1
unassigned	T	0	0	0	0	1	0	0	0	0	0	0	0	0	0	1		1	
	B	0	0	0	0	0	0	0	0	0	0	0	0	0	0	0	1	0	1
Total isolates		36	16	25	30	46	25	29	30	17	33	16	34	22	39	398	398	14	14
Total classes		3	1	3	4	6	2	6	3	3	3	3	4	2	3				

Figure S2. Number of isolates belonging to the cultured *Fusarium graminearum*-infected transmitting silk microbiome at the class taxonomic level. Isolates were cultured separately from the tip (T) and base (B) of maize silks spanning diverse host inbred/hybrid lines and heterotic groups. Yellow cells indicate the presence of isolate(s). Green cells indicate that the class is unique to the host genotype. Prevalence refers to the number of maize genotypes that gave rise to at least one cultured isolate from that class. Further details are in Table S1.

Heterotic Group		European Flint	Early Butler	BSSS	BSSS/Minnesota 13	Minnesota 13	Minnesota 13	Minnesota 13	P3990	P3990/Iodent	Iodent	Lancaster	Pioneer Hybrid	Pioneer Hybrid	Pioneer Hybrid				
Maize Inbred/ Hybrid Line / Bacterial Orders	Tip/Base	CO444	CO325	CO452	CO462	CO449	CO433	CO432	CO430	CO448	CO431	CO441	P35837	P38157	P9855HR	Total isolates	Total isolates, merged Tip and Base	Prevalence	Prevalance, merged Tip and Base
Bacillales	T	0	0	0	0	0	0	0	0	0	0	0	0	0	0	0		0	
	B	0	0	0	0	0	0	1	0	0	0	0	2	0	0	3	3	2	2
Burkholderiales	T	6	0	3	3	6	0	1	0	4	0	2	1	0	0	26		8	
	B	0	0	1	0	1	0	1	0	0	0	0	0	0	0	3	29	3	8
Caulobacterales	T	0	0	0	0	0	0	1	0	0	0	0	0	0	0	1		1	
	B	0	0	0	0	0	0	0	0	0	0	0	0	0	0	0	1	0	1
Cytophagales	T	0	0	0	0	0	0	0	0	0	0	0	0	0	0	0		0	
	B	0	0	0	0	0	0	0	0	0	0	0	0	0	0	0	0	0	0
Enterobacterales	T	9	12	14	11	7	16	9	13	6	6	7	13	8	13	144		14	
	B	9	2	1	1	12	2	5	8	1	10	3	4	2	9	69	213	14	14
Flavobacteriales	T	0	0	0	1	0	0	0	0	0	2	0	0	0	1	4		3	
	B	0	0	0	0	0	0	1	0	0	2	0	0	0	0	3	7	2	4
Hyphomicrobiales	T	0	0	0	0	2	0	0	0	0	0	0	1	0	0	3		2	
	B	0	0	0	0	0	0	0	0	0	3	0	0	0	0	3	6	1	3
Lactobacillales	T	0	0	0	0	2	1	1	0	0	0	0	0	0	3	7		4	
	B	0	0	0	0	1	0	0	0	0	0	0	0	0	0	1	8	1	4
Lysobacterales	T	0	0	0	4	0	0	0	0	0	0	0	0	0	0	4		1	
	B	0	0	0	0	0	0	0	0	0	0	0	0	0	0	0	4	0	1
Micrococcales	T	0	0	0	0	1	0	1	2	0	0	0	0	0	0	4		3	
	B	0	0	0	0	0	0	1	1	0	0	0	0	0	0	2	6	2	3
Mycobacteriales	T	0	0	0	0	0	0	0	0	0	0	0	0	0	0	0		0	
	B	0	0	1	0	0	0	0	0	0	0	0	0	0	0	1	1	1	1
Propionibacteriales	T	0	0	0	0	0	0	0	0	0	0	0	0	0	0	0		0	
	B	0	0	0	0	0	0	0	0	0	0	0	0	0	0	0	0	0	0
Pseudomonadales	T	2	2	0	1	5	2	0	0	0	4	0	6	1	3	26		9	
	B	1	0	2	0	0	0	1	0	2	0	0	1	2	3	12	38	7	12
Rhodospirillales	T	0	0	0	3	0	0	0	0	0	0	0	0	0	0	3		1	
	B	0	0	0	0	0	0	0	0	1	0	0	0	0	0	1	4	1	2
Sphingobacteriales	T	0	0	0	0	1	0	0	0	0	0	0	0	0	0	1		1	
	B	0	0	0	0	1	0	0	0	0	0	0	0	0	0	1	2	1	1
Sphingomonadales	T	1	0	0	0	1	0	3	2	0	1	1	0	0	0	9		6	
	B	0	0	0	0	0	0	2	1	0	0	0	0	1	0	4	13	3	7
Xanthomonadales	T	7	0	3	2	3	2	1	1	2	2	2	3	5	5	38		13	
	B	1	0	0	4	2	2	0	2	1	3	1	3	3	2	24	62	11	13
unassigned	T	0	0	0	0	1	0	0	0	0	0	0	0	0	0	1		1	
	B	0	0	0	0	0	0	0	0	0	0	0	0	0	0	0	1	0	1
Total isolates		36	16	25	30	46	25	29	30	17	33	16	34	22	39	398	398	14	14
Total orders		5	2	5	7	9	4	10	4	5	6	4	6	4	5				

Figure S3. Number of isolates belonging to the cultured *Fusarium graminearum*-infected transmitting silk microbiome at the order taxonomic level. Isolates were cultured separately from the tip (T) and base (B) of maize silks spanning diverse host inbred/hybrid lines and heterotic groups. Yellow cells indicate the presence of isolate(s). Green cells indicate that the order is unique to the host genotype. Prevalence refers to the number of maize genotypes that gave rise to at least one cultured isolate from that order. Further details are in Table S1.

Heterotic Group		European Flint	Early Butler	BSSS	BSSS/Minnesota 13	Minnesota 13	Minnesota 13	Minnesota 13	P3990	P3990/Iodent	Iodent	Lancaster	Pioneer Hybrid	Pioneer Hybrid	Pioneer Hybrid				
Maize Inbred/ Hybrid Line / Bacterial Families	Tip/Base	CO444	CO325	CO452	CO462	CO449	CO433	CO432	CO430	CO448	CO431	CO441	P35837	P38157	P9855HR	Total isolates	Total isolates, merged Tip and Base	Prevalence	Prevalance, merged Tip and Base
Acetobacteraceae	T	0	0	0	3	0	0	0	0	0	0	0	0	0	0	3		1	
	B	0	0	0	0	0	0	0	0	1	0	0	0	0	0	1	4	1	2
Alcaligenaceae	T	0	0	0	0	0	0	0	0	0	0	0	0	0	0	0		0	
	B	0	0	0	0	0	0	0	0	0	0	0	0	0	0	0	0	0	0
Aurantimonadaceae	T	0	0	0	0	0	0	0	0	0	0	0	0	0	0	0		0	
	B	0	0	0	0	0	0	0	0	0	0	0	0	0	0	0	0	0	0
Bacillaceae	T	0	0	0	0	0	0	0	0	0	0	0	0	0	0	0		0	
	B	0	0	0	0	0	0	0	0	0	0	0	2	0	0	2	2	1	1
Beutenbergiaceae	T	0	0	0	0	0	0	0	0	0	0	0	0	0	0	0		0	
	B	0	0	0	0	0	0	0	0	0	0	0	0	0	0	0	0	0	0
Caulobacteraceae	T	0	0	0	0	0	0	1	0	0	0	0	0	0	0	1		1	
	B	0	0	0	0	0	0	0	0	0	0	0	0	0	0	0	1	0	1
Comamonadaceae	T	6	0	3	3	6	0	0	0	4	0	2	1	0	0	25		7	
	B	0	0	1	0	1	0	1	0	0	0	0	0	0	0	3	28	3	8
Enterobacteriaceae	T	3	4	12	3	0	16	5	8	4	2	6	7	2	6	78		13	
	B	2	1	0	1	2	1	0	5	1	0	2	2	1	2	20	98	11	14
Enterococcaceae	T	0	0	0	0	0	0	0	0	0	0	0	0	0	0	0		0	
	B	0	0	0	0	0	0	0	0	0	0	0	0	0	0	0	0	0	0
Erwiniaceae	T	6	8	2	8	6	0	4	5	2	4	1	6	6	7	65		13	
	B	5	1	0	0	5	0	5	3	0	10	1	1	1	6	38	103	10	13
Flavobacteriaceae	T	0	0	0	1	0	0	0	0	0	0	0	0	0	0	1		1	
	B	0	0	0	0	0	0	0	0	0	0	0	0	0	0	0	1	0	1
Gordoniaceae	T	0	0	0	0	0	0	0	0	0	0	0	0	0	0	0		0	
	B	0	0	0	0	0	0	0	0	0	0	0	0	0	0	0	0	0	0
Hafniaceae	T	0	0	0	0	0	0	0	0	0	0	0	0	0	0	0		0	
	B	0	0	0	0	0	0	0	0	0	0	0	1	0	1	2	2	2	2
Intrasporangiaceae	T	0	0	0	0	0	0	0	0	0	0	0	0	0	0	0		0	
	B	0	0	0	0	0	0	0	0	0	0	0	0	0	0	0	0	0	0
Methylobacteriaceae	T	0	0	0	0	0	0	0	0	0	0	0	0	0	0	0		0	
	B	0	0	0	0	0	0	0	0	0	0	0	0	0	0	0	0	0	0
Microbacteriaceae	T	0	0	0	0	1	0	1	2	0	0	0	0	0	0	4		3	
	B	0	0	0	0	0	0	1	1	0	0	0	0	0	0	2	6	2	3
Micrococcaceae	T	0	0	0	0	0	0	0	0	0	0	0	0	0	0	0		0	
	B	0	0	0	0	0	0	0	0	0	0	0	0	0	0	0	0	0	0
Moraxellaceae	T	1	0	0	0	3	1	0	0	0	1	0	3	0	0	9		5	
	B	0	0	0	0	0	0	0	0	0	0	0	0	0	0	0	9	0	5
Nocardioidaceae	T	0	0	0	0	0	0	0	0	0	0	0	0	0	0	0		0	
	B	0	0	1	0	0	0	0	0	0	0	0	0	0	0	1	1	1	1
Oxalobacteraceae	T	0	0	0	0	0	0	1	0	0	0	0	0	0	0	1		1	
	B	0	0	0	0	0	0	0	0	0	0	0	0	0	0	0	1	0	1
Paenibacillaceae	T	0	0	0	0	0	0	0	0	0	0	0	0	0	0	0		0	
	B	0	0	0	0	0	0	1	0	0	0	0	0	0	0	1	1	1	1
Pseudomonadaceae	T	1	2	0	1	2	1	0	0	0	3	0	3	1	3	17		9	
	B	1	0	2	0	0	0	1	0	2	0	0	1	2	3	12	29	7	12
Rhizobiaceae	T	0	0	0	0	2	0	0	0	0	0	0	1	0	0	3		2	
	B	0	0	0	0	0	0	0	0	0	3	0	0	0	0	3	6	1	3
Rhodanobacteraceae	T	0	0	0	4	0	0	0	0	0	0	0	0	0	0	4		1	
	B	0	0	0	0	0	0	0	0	0	0	0	0	0	0	0	4	0	1
Sphingobacteriaceae	T	0	0	0	0	1	0	0	0	0	0	0	0	0	0	1		1	
	B	0	0	0	0	1	0	0	0	0	0	0	0	0	0	1	2	1	1
Sphingomonadaceae	T	1	0	0	0	1	0	3	2	0	1	1	0	0	0	9		6	
	B	0	0	0	0	0	0	2	1	0	0	0	0	1	0	4	13	3	7
Spirosomaceae	T	0	0	0	0	0	0	0	0	0	0	0	0	0	0	0		0	
	B	0	0	0	0	0	0	0	0	0	0	0	0	0	0	0	0	0	0
Streptococcaceae	T	0	0	0	0	2	1	1	0	0	0	0	0	0	3	7		4	
	B	0	0	0	0	1	0	0	0	0	0	0	0	0	0	1	8	1	4
Weeksellaceae	T	0	0	0	0	0	0	0	0	0	2	0	0	0	1	3		2	
	B	0	0	0	0	0	0	1	0	0	2	0	0	0	0	3	6	2	3
Xanthomonadaceae	T	7	0	3	2	3	2	1	1	2	2	2	3	5	5	38		13	
	B	1	0	0	4	2	2	0	2	1	3	1	3	3	2	24	62	11	13
Yersiniaceae	T	0	0	0	0	1	0	0	0	0	0	0	0	0	0	1		1	
	B	2	0	1	0	5	1	0	0	0	0	0	0	0	0	9	10	4	4
unassigned	T	0	0	0	0	1	0	0	0	0	0	0	0	0	0	1		1	
	B	0	0	0	0	0	0	0	0	0	0	0	0	0	0	0	1	0	1
Total isolates		36	16	25	30	46	25	29	30	17	33	16	34	22	39	398	398	14	14
Total families		8	3	7	8	12	6	12	5	6	8	5	9	5	7				

Figure S4. Number of isolates belonging to the cultured *Fusarium graminearum*-infected transmitting silk microbiome at the family taxonomic level. Isolates were cultured separately from the tip (T) and base (B) of maize silks spanning diverse host inbred/hybrid lines and heterotic groups. Yellow cells indicate the presence of isolate(s). Green cells indicate that the family is unique to the host genotype. Prevalence refers to the number of maize genotypes that gave rise to at least one cultured isolate from that family. Further details are in Table S1.

(A)

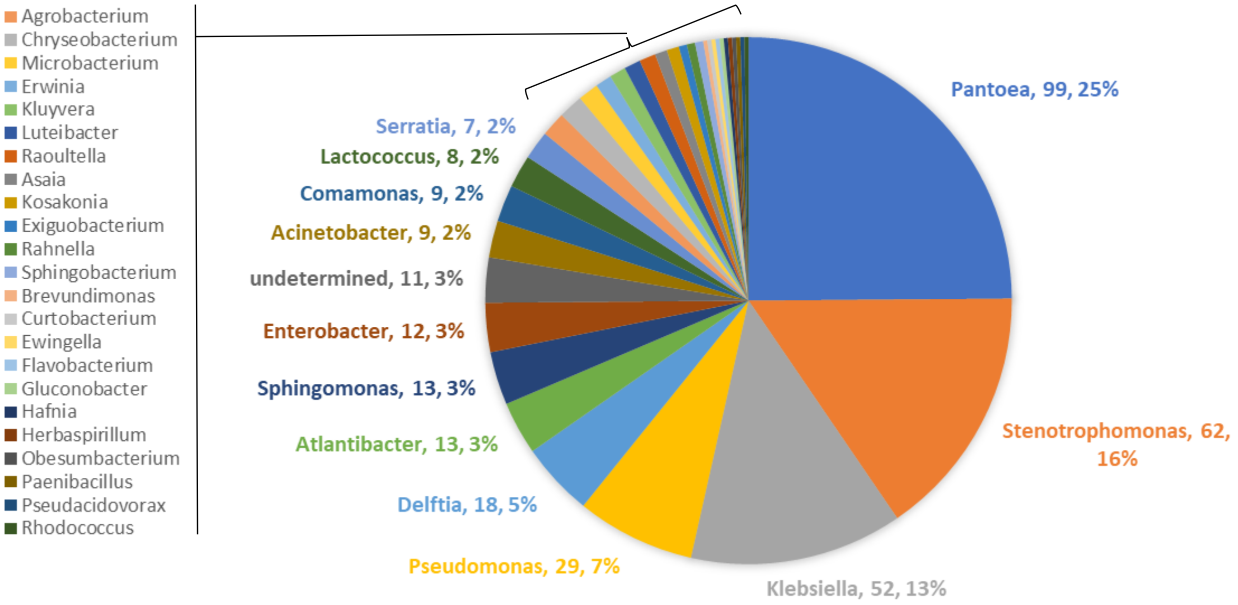

(B)

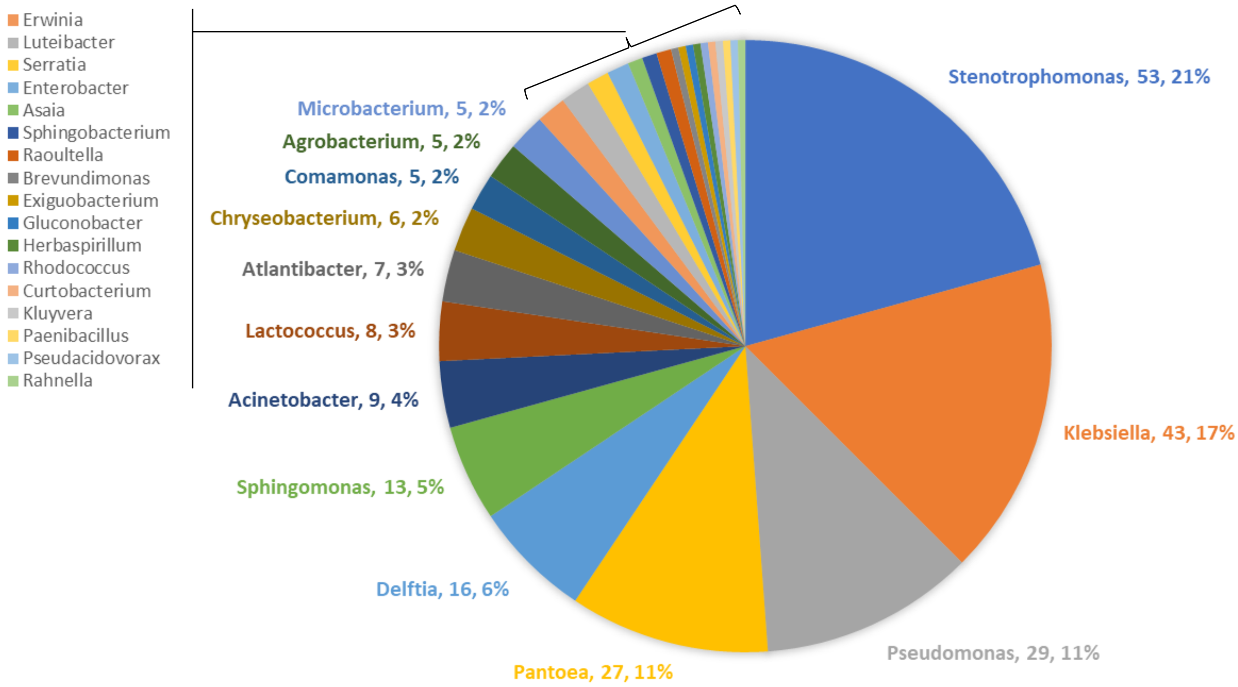

Figure S5. Genus level taxonomic identification of the cultured transmitting silk microbiome from *Fusarium graminearum*-infected maize silks spanning diverse heterotic groups. **(A)** Predicted genera (top match) at the moderate stringency threshold (398 isolates). **(B)** Predicted genera at the high threshold stringency (256 isolates). See Methods for threshold definitions. Further details are in Table S1.

(A)

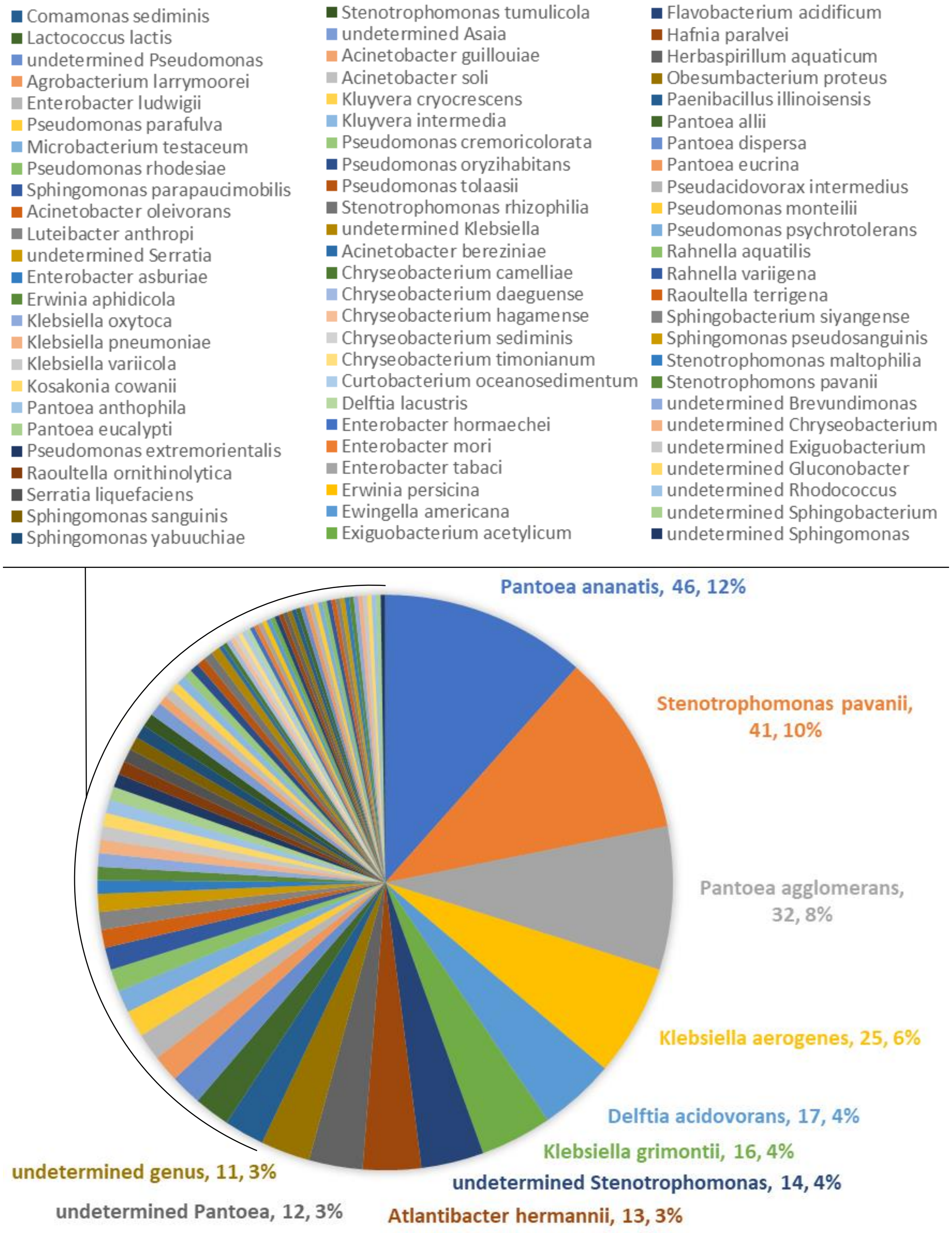
Comamonas sediminis
Lactococcus lactis
undetermined Pseudomonas
Agrobacterium larrymoorei
Enterobacter ludwigii
Pseudomonas parafulva
Microbacterium testaceum
Pseudomonas rhodesiae
Sphingomonas parapaucimobilis
Acinetobacter oleivorans
Luteibacter anthropi
undetermined Serratia
Enterobacter asburiae
Erwinia aphidicola
Klebsiella oxytoca
Klebsiella pneumoniae
Klebsiella variicola
Kosakonia cowanii
Pantoea anthophila
Pantoea eucalypti
Pseudomonas extremorientalis
Raoultella ornithinolytica
Serratia liquefaciens
Sphingomonas sanguinis
Sphingomonas yabuuchiae
Stenotrophomonas tumulicola
undetermined Asaia
Acinetobacter guillouiae
Acinetobacter soli
Kluyvera cryocrescens
Kluyvera intermedia
Pseudomonas cremoricolorata
Pseudomonas oryzihabitans
Pseudomonas tolaasii
Stenotrophomonas rhizophilia
undetermined Klebsiella
Acinetobacter bereziniae
Chryseobacterium camelliae
Chryseobacterium daeguense
Chryseobacterium hagamense
Chryseobacterium sediminis
Chryseobacterium timonianum
Curtobacterium oceanosedimentum
Delftia lacustris
Enterobacter hormaechei
Enterobacter mori
Enterobacter tabaci
Erwinia persicina
Ewingella americana
Exiguobacterium acetylicum
Flavobacterium acidificum
Hafnia paralvei
Herbaspirillum aquaticum
Obesumbacterium proteus
Paenibacillus illinoisensis
Pantoea allii
Pantoea dispersa
Pantoea eucrina
Pseudacidovorax intermedius
Pseudomonas monteilii
Pseudomonas psychrotolerans
Rahnella aquatilis
Rahnella variigena
Raoultella terrigena
Sphingobacterium siyangense
Sphingomonas pseudosanguinis
Stenotrophomonas maltophilia
Stenotrophomons pavanii
undetermined Brevundimonas
undetermined Chryseobacterium
undetermined Exiguobacterium
undetermined Gluconobacter
undetermined Rhodococcus
undetermined Sphingobacterium
undetermined Sphingomonas
Pantoea ananatis, 46, 12%
Stenotrophomonas pavanii, 41, 10%
Pantoea agglomerans, 32, 8%
Klebsiella aerogenes, 25, 6%
Delftia acidovorans, 17, 4%
Klebsiella grimontii, 16, 4%
undetermined Stenotrophomonas, 14, 4%
Atlantibacter hermannii, 13, 3%
undetermined Pantoea, 12, 3%
undetermined genus, 11, 3%

(B)

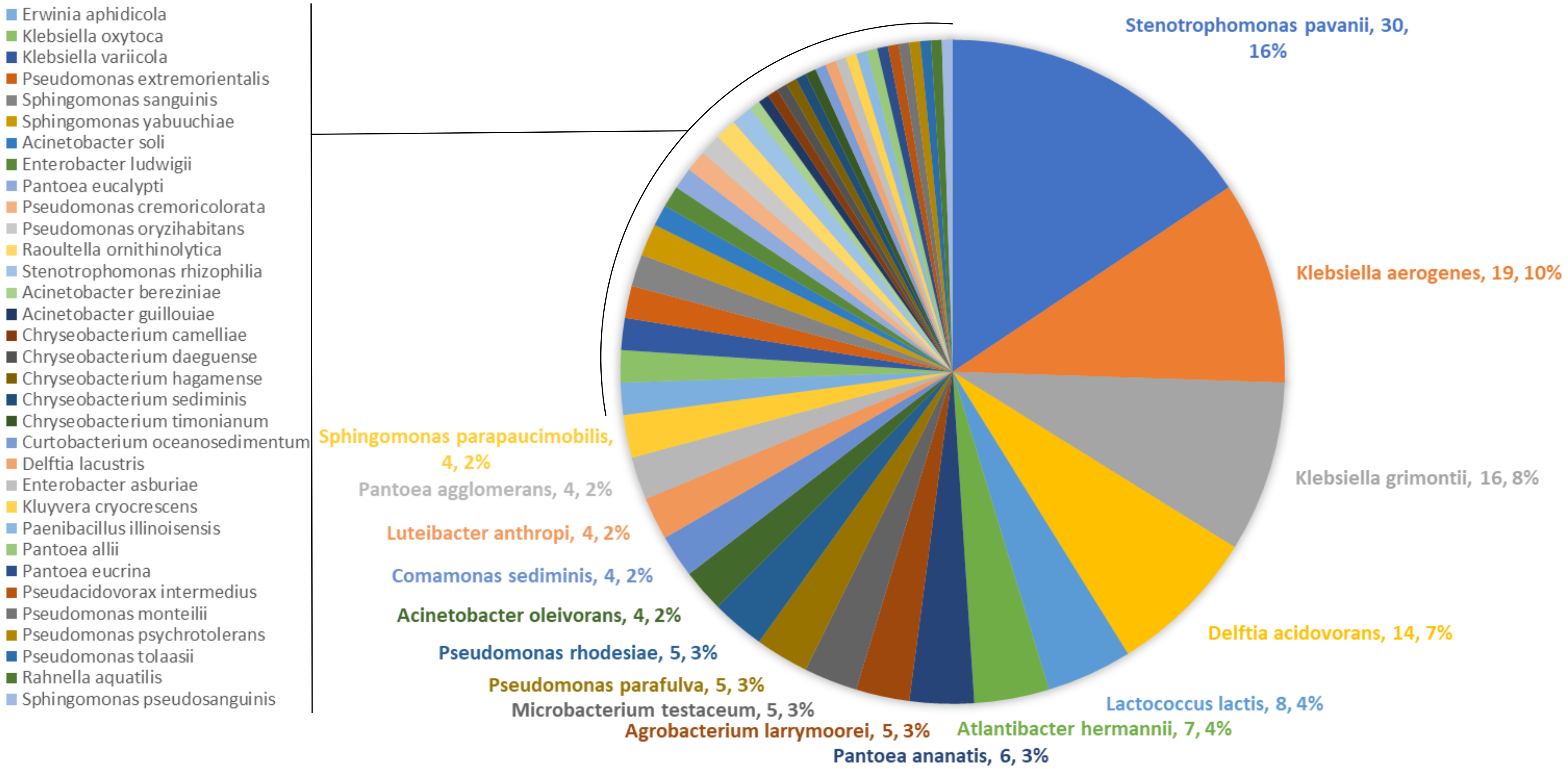

Erwinia aphidicola
Klebsiella oxytoca
Klebsiella variicola
Pseudomonas extremorientalis
Sphingomonas sanguinis
Sphingomonas yabuuchiae
Acinetobacter soli
Enterobacter ludwigii
Pantoea eucalypti
Pseudomonas cremoricolorata
Pseudomonas oryzihabitans
Raoultella ornithinolytica
Stenotrophomonas rhizophilia
Acinetobacter bereziniae
Acinetobacter guillouiae
Chryseobacterium camelliae
Chryseobacterium daeguense
Chryseobacterium hagamense
Chryseobacterium sediminis
Chryseobacterium timonianum
Curtobacterium oceanosedimentum
Delftia lacustris
Enterobacter asburiae
Kluyvera cryocrescens
Paenibacillus illinoisensis
Pantoea allii
Pantoea eucrina
Pseudacidovorax intermedius
Pseudomonas monteilii
Pseudomonas psychrotolerans
Pseudomonas tolaasii
Rahnella aquatilis
Sphingomonas pseudosanguinis
Stenotrophomonas pavanii, 30, 16%
Klebsiella aerogenes, 19, 10%
Klebsiella grimontii, 16, 8%
Delftia acidovorans, 14, 7%
Lactococcus lactis, 8, 4%
Atlantibacter hermannii, 7, 4%
Pantoea ananatis, 6, 3%
Agrobacterium larrymoorei, 5, 3%
Microbacterium testaceum, 5, 3%
Pseudomonas parafulva, 5, 3%
Pseudomonas rhodesiae, 5, 3%
Acinetobacter oleivorans, 4, 2%
Comamonas sediminis, 4, 2%
Luteibacter anthropi, 4, 2%
Pantoea agglomerans, 4, 2%
Sphingomonas parapaucimobilis, 4, 2%

(C)

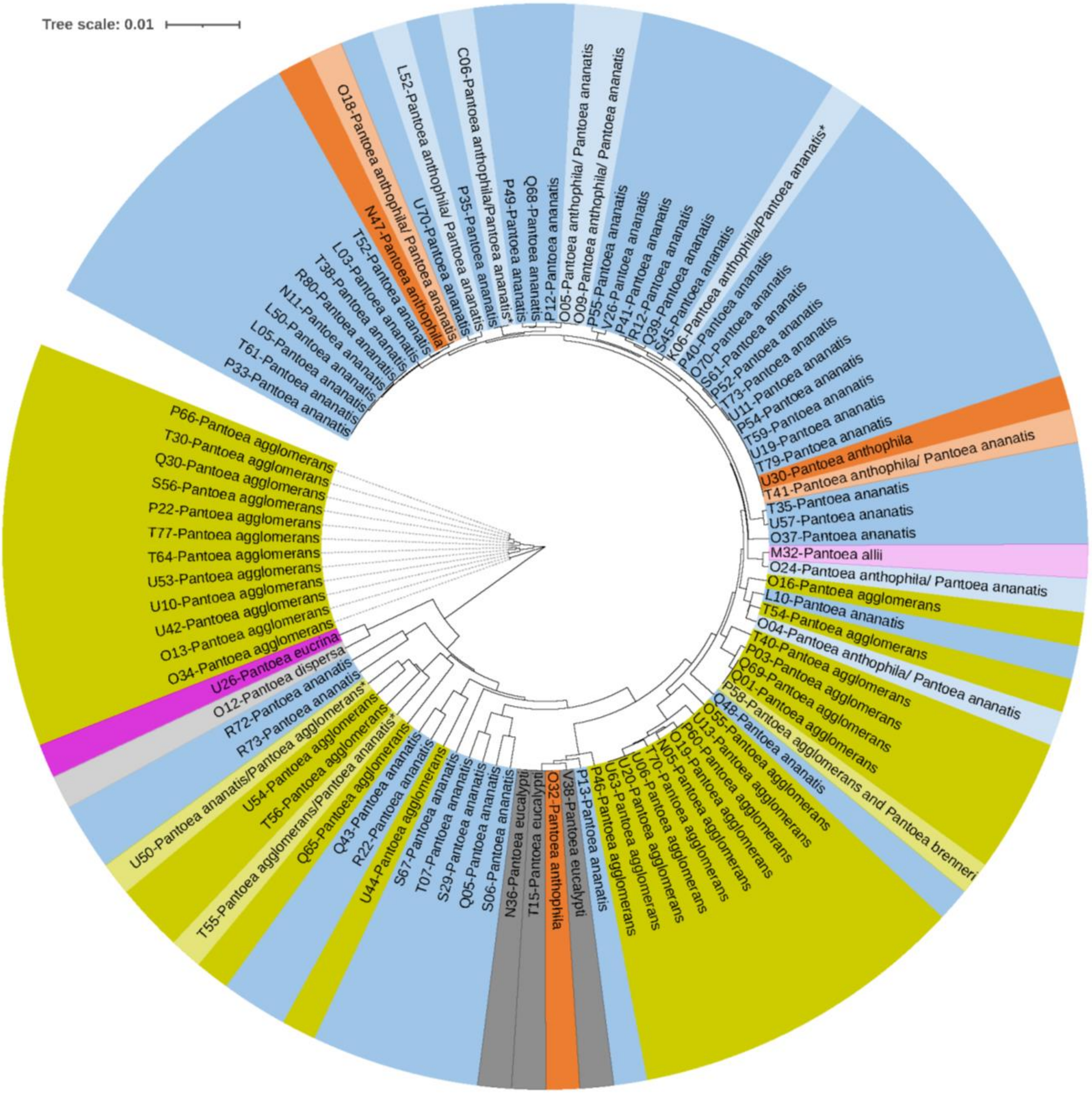

Figure S6. Species level taxonomic identification of the cultured transmitting silk microbiome from *Fusarium graminearum*-infected maize silks spanning diverse heterotic groups. **(A)** Predicted species (top match) at the moderate stringency threshold (398 isolates). **(B)** Predicted species at the high threshold stringency (192 isolates). See Methods for threshold definitions. **(C)** Phylogenetic tree of the isolates identified as *Pantoea* from the cultured microbiome of *F. graminearum*-infected transmitting maize silks. Isolates were cultured separately from the tip (T) and base (B) of maize silks spanning diverse host inbred/hybrid lines and heterotic groups. Species were predicted based on first matches from NCBI BLAST searches. Isolates that were not grouped alongside isolates with the same species name (e.g. *Pantoea anthophila*) are not likely misaligned; it is more likely that the species assignment was inaccurate due to short sequence lengths. Due to a short sequence length, isolate R15 (*Pantoea ananatis*) could not be included in the tree. Further details are in Table S1.

Heterotic Group		European Flint	Early Butler	BSSS	BSSS/Minnesota 13	Minnesota 13	Minnesota 13	Minnesota 13	P3990	P3990/Iodent	Iodent	Lancaster	Pioneer Hybrid	Pioneer Hybrid	Pioneer Hybrid				
Maize Inbred/ Hybrid Line / Bacterial Species	Tip/Base	CO444	CO325	CO452	CO462	CO449	CO433	CO432	CO430	CO448	CO431	CO441	P35837	P38157	P9855HR	Total isolates	Total isolates, merged Tip and Base	Prevalence	Prevalance, merged Tip and Base
Agrobacterium larrymoorei	T	0	0	0	0	2	0	0	0	0	0	0	1	0	0	3		2	
	B	0	0	0	0	0	0	0	0	0	3	0	0	0	0	3	6	1	3
Atlantibacter hermannii	T	0	0	0	1	0	0	1	0	1	0	0	4	0	2	9		5	
	B	1	0	0	0	0	0	0	1	0	0	0	0	0	2	4	13	3	7
Delftia acidovorans	T	6	0	3	1	0	0	0	0	4	0	2	1	0	0	17		6	
	B	0	0	0	0	0	0	0	0	0	0	0	0	0	0	0	17	0	6
Enterobacter asburiae	T	0	1	0	1	0	1	0	0	0	0	0	0	0	0	3		3	
	B	0	0	0	0	0	0	0	0	0	0	0	0	0	0	0	3	0	3
Klebsiella aerogenes	T	0	0	8	0	0	6	0	3	3	0	3	0	0	0	23		5	
	B	0	0	0	0	0	0	0	1	0	0	1	0	0	0	2	25	2	5
Klebsiella grimontii	T	0	3	2	0	0	4	4	0	0	0	0	0	1	1	15		6	
	B	1	0	0	0	0	0	0	0	0	0	0	0	0	0	1	16	1	7
Lactococcus lactis	T	0	0	0	0	2	1	1	0	0	0	0	0	0	3	7		4	
	B	0	0	0	0	1	0	0	0	0	0	0	0	0	0	1	8	1	4
Microbacterium testaceum	T	0	0	0	0	1	0	0	2	0	0	0	0	0	0	3		2	
	B	0	0	0	0	0	0	1	1	0	0	0	0	0	0	2	5	2	3
Pantoea agglomerans	T	1	4	0	4	0	0	2	1	0	2	0	0	0	3	17		7	
	B	5	1	0	0	2	0	4	0	0	2	0	0	0	1	15	32	6	8
Pantoea ananatis	T	3	3	2	2	4	0	2	4	1	2	1	5	2	3	34		13	
	B	0	0	0	0	1	0	0	3	0	7	0	0	0	1	12	46	4	13
Pantoea anthophila	T	1	0	0	0	0	0	0	0	0	0	0	1	0	0	2		2	
	B	0	0	0	0	1	0	0	0	0	0	0	0	0	0	1	3	1	3
Pantoea eucalypti	T	0	0	0	0	1	0	0	0	0	0	0	0	1	1	3		3	
	B	0	0	0	0	0	0	0	0	0	0	0	0	0	0	0	3	0	3
Pseudomonas parafulva	T	1	0	0	0	1	1	0	0	0	1	0	0	1	0	5		5	
	B	1	0	0	0	0	0	0	0	0	0	0	0	0	0	1	6	1	5
Pseudomonas rhodesiae	T	0	0	0	1	0	0	0	0	0	0	0	0	0	0	1		1	
	B	0	0	0	0	0	0	0	0	2	0	0	0	2	0	4	5	2	3
Sphingomonas parapaucimobilis	T	1	0	0	0	0	0	2	1	0	1	0	0	0	0	5		4	
	B	0	0	0	0	0	0	0	0	0	0	0	0	0	0	0	5	0	4
Sphingomonas yabuuchiae	T	0	0	0	0	1	0	1	0	0	0	1	0	0	0	3		3	
	B	0	0	0	0	0	0	0	0	0	0	0	0	0	0	0	3	0	3
Stenotrophomonas pavanii	T	7	0	3	0	0	1	1	0	2	2	2	2	5	3	28		10	
	B	1	0	0	2	2	0	0	2	0	3	1	1	0	1	13	41	8	13

Figure S7. Number of isolates belonging to the cultured *Fusarium graminearum*-infected transmitting silk microbiome at the species taxonomic level. Isolates were cultured separately from the tip (T) and base (B) of maize silks spanning diverse host inbred/hybrid lines and heterotic groups. Only species which were cultured from 3 or more maize genotypes were included in this figure. Yellow-to-green cells indicate the presence of isolate(s). Red cells indicate the absence of isolates. Prevalence refers to the number of maize genotypes that gave rise to at least one cultured isolate from that species. Further details are in Table S1.

Silk Type		Healthy		*Fg*-infected	
Maize Inbred/ Hybrid Line / Bacterial Families	Tip/Base	Total isolates	Total isolates, merged Tip and Base	Total isolates	Total isolates, merged Tip and Base
Acetobacteraceae	T	0	1	3	4
	B	1		1	
Alcaligenaceae	T	0	1	0	0
	B	1		0	
Aurantimonadaceae	T	0	1	0	0
	B	1		0	
Bacillaceae	T	8	13	0	2
	B	5		2	
Beutenbergiaceae	T	0	1	0	0
	B	1		0	
Caulobacteraceae	T	1	4	1	1
	B	3		0	
Comamonadaceae	T	2	7	25	28
	B	5		3	
Enterobacteriaceae	T	38	69	78	98
	B	31		20	
Enterococcaceae	T	4	6	0	0
	B	2		0	
Erwiniaceae	T	42	78	65	103
	B	36		38	
Flavobacteriaceae	T	1	1	1	1
	B	0		0	
Gordoniaceae	T	0	1	0	0
	B	1		0	
Hafniaceae	T	0	0	0	2
	B	0		2	
Intrasporangiaceae	T	2	2	0	0
	B	0		0	
Methylobacteriaceae	T	0	1	0	0
	B	1		0	
Microbacteriaceae	T	23	39	4	6
	B	16		2	

Silk Type		Healthy		*Fg*-infected	
Maize Inbred/ Hybrid Line / Bacterial Families	Tip/Base	Total isolates	Total isolates, merged Tip and Base	Total isolates	Total isolates, merged Tip and Base
Micrococcaceae	T	0	1	0	0
	B	1		0	
Moraxellaceae	T	3	5	9	9
	B	2		0	
Nocardioidaceae	T	1	5	0	1
	B	4		1	
Oxalobacteraceae	T	0	0	1	1
	B	0		0	
Paenibacillaceae	T	0	1	0	1
	B	1		1	
Pseudomonadaceae	T	5	12	17	29
	B	7		12	
Rhizobiaceae	T	8	13	3	6
	B	5		3	
Rhodanobacteraceae	T	0	0	4	4
	B	0		0	
Sphingobacteriaceae	T	2	4	1	2
	B	2		1	
Sphingomonadaceae	T	5	11	9	13
	B	6		4	
Spirosomaceae	T	1	1	0	0
	B	0		0	
Streptococcaceae	T	17	24	7	8
	B	7		1	
Weeksellaceae	T	10	17	3	6
	B	7		3	
Xanthomonadaceae	T	12	24	38	62
	B	12		24	
Yersiniaceae	T	3	5	1	10
	B	2		9	
unassigned	T	0	2	1	1
	B	2		0	
Total isolates		350		398	
Total families		28		23	

Figure S8. Comparison of number of isolates belonging to the cultured healthy (non-infected) and *Fusarium graminearum*-infected transmitting silk microbiome at the family taxonomic level. Isolates were cultured separately from the tip (T) and base (B) of maize silks spanning diverse host inbred/hybrid lines and heterotic groups. Yellow-to-green cells indicate the presence of isolate(s). Red cells indicate the absence of isolates. Further details are in Table S1.

Heterotic Group		European Flint	Early Butler	BSSS	BSSS/ Minnesota 13	Minnesota 13	Minnesota 13	Minnesota 13	P3990	P3990/Iodent	Iodent	Lancaster	Pioneer Hybrid	Pioneer Hybrid	Pioneer Hybrid			
Bacterial Species \ Maize Inbred/ Hybrid Line	Tip/Base	CO444	CO325	CO452	CO462	CO449	CO433	CO432	CO430	CO448	CO431	CO441	P35837	P38157	P9855HR	Prevalence of species within tip/base divisions of genotypes	Prevalance of species within genotypes, merged Tip and Base	Number of unique OTUs within the species
Agrobacterium larrymoorei	T	0	0	0	0	12, 14, 393	0	0	0	0	0	0	13	0	0	2		
	B	0	0	0	0	0	0	0	0	0	12, 390	0	0	0	0	1	3	5
Atlantibacter hermannii	T	0	0	0	23	0	0	18, 403	0	17, 404	0	0	17, 28	0	22, 27	5		
	B	30	0	0	0	0	0	0	26	0	0	0	0	0	29, 154, 403, 404	3	7	12
Delftia acidovorans	T	62, 63, 65, 67, 392	0	62, 392	62, 63, 392	0	0	0	0	62, 65, 67, 69, 392	0	62, 66	64, 70	0	0	6		
	B	0	0	0	0	0	0	0	0	0	0	0	0	0	0	0	6	9
Enterobacter asburiae	T	0	203	0	71, 74, 203	0	73	0	0	0	0	0	0	0	0	3		
	B	0	0	0	0	0	0	0	0	0	0	0	0	0	0	0	3	4
Klebsiella aerogenes	T	0	0	117, 118, 119, 121, 122, 124, 125, 399, 405	0	0	117, 118, 119, 121, 122, 124, 125, 126	0	118, 127	121, 124, 128	0	117, 118, 122, 125	0	0	0	5		
	B	0	0	0	0	0	0	0	123	0	0	118	0	0	0	2	5	13
Klebsiella grimontii	T	0	134, 136, 138	130, 132	0	0	130, 132, 133, 138, 407	130, 132, 133, 138, 397, 407	0	0	0	0	0	130, 136, 138	129	6		
	B	130, 138	0	0	0	0	0	0	0	0	0	0	0	0	0	1	7	9
Lactococcus lactis	T	0	0	0	0	158	158	158, 159	0	0	0	0	0	0	158, 159, 160, 161, 162, 163	4		
	B	0	0	0	0	158	0	0	0	0	0	0	0	0	0	1	4	6
Microbacterium testaceum	T	0	0	0	0	187	0	0	190, 191	0	0	0	0	0	0	2		
	B	0	0	0	0	0	0	191	190, 191	0	0	0	0	0	0	2	3	3
Pantoea agglomerans	T	202, 204, 205	203, 227, 246, 402	0	204, 232, 233, 238, 241, 256, 401	0	0	201	250	0	230, 235	0	0	0	201, 257	7		
	B	201, 248, 260, 261	293	0	0	202, 205	0	200, 253, 259, 260, 261	0	0	214, 230	0	0	0	237	6	8	27

Heterotic Group		European Flint	Early Butler	BSSS	BSSS/ Minnesota 13	Minnesota 13	Minnesota 13	Minnesota 13	P3990	P3990/Iodent	Iodent	Lancaster	Pioneer Hybrid	Pioneer Hybrid	Pioneer Hybrid	Prevalence of species within tip/base divisions of genotypes	Prevalance of species within genotypes, merged Tip and Base	Number of unique OTUs within the species
Maize Inbred/ Hybrid Line / Bacterial Species	Tip/Base	CO444	CO325	CO452	CO462	CO449	CO433	CO432	CO430	CO448	CO431	CO441	P35837	P38157	P9855HR			
Pantoea ananatis	T	289, 290, 291, 292	272, 289, 290, 291, 292	268, 270, 271, 289, 290, 291, 292	271, 289, 290, 291, 292	264, 265, 267, 270, 276, 291, 292	0	280, 396	263, 264, 265, 267, 270, 276, 291, 296	289, 290, 291, 292	270, 276, 291	268, 271, 289	270, 271, 276, 277, 281, 284, 285, 289, 290, 291, 292	264, 265, 267, 270, 276, 291	262, 270, 271, 279, 289, 290, 291, 292	13		
	B	0	0	0	0	289, 290, 291, 292	0	0	219, 265, 267, 270, 276, 291	0	264, 265, 267, 271, 276, 288, 291, 410	0	0	0	289, 290, 291, 292	4	13	25
Pantoea anthophila	T	289, 290, 291, 292	0	0	0	0	0	0	0	0	0	0	288, 289, 290, 291, 292, 410	0	0	2		
	B	0	0	0	0	219, 220	0	0	0	0	0	0	0	0	0	1	3	8
Pantoea eucalypti	T	0	0	0	0	215	0	0	0	0	0	0	0	219, 220, 223	220	3		
	B	0	0	0	0	0	0	0	0	0	0	0	0	0	0	0	3	4
Pseudomonas parafulva	T	317	0	0	0	317	314	0	0	0	312	0	0	317	0	5		
	B	317	0	0	0	0	0	0	0	0	0	0	0	0	0	1	5	3
Pseudomonas rhodesiae	T	0	0	0	320	0	0	0	0	0	0	0	0	0	0	1		
	B	0	0	0	0	0	0	0	0	318, 320	0	0	0	320	0	2	3	2
Sphingomonas parapaucimobilis	T	363	0	0	0	0	0	358, 359	358, 359, 360	0	361	0	0	0	0	4		
	B	0	0	0	0	0	0	0	0	0	0	0	0	0	0	0	4	5
Sphingomonas yabuuchiae	T	0	0	0	0	357	0	362	0	0	0	356	0	0	0	3		
	B	0	0	0	0	0	0	0	0	0	0	0	0	0	0	0	3	3
Stenotrophomonas pavanii	T	366, 367, 368, 370, 375	0	367, 370, 373, 374, 375	0	0	375	375	0	374, 375	374, 375	367, 374	367, 368, 370, 372, 373, 375	367, 368, 370, 374, 375	374, 375	10		
	B	375	0	0	368, 374, 375	367, 368, 375	0	0	375	0	372, 373, 374, 375	370	375	0	367, 368, 370, 375	8	13	8
Total unique OTUs (from all species, not just those listed above)		31	18	34	38	46	29	36	28	25	31	28	46	30	55			

Figure S9. Cultured OTUs identified within bacterial species in the cultured *Fusarium graminearum*-infected transmitting silk microbiome. Isolates were cultured separately from the tip (T) and base (B) of maize silks spanning diverse host inbred/hybrid lines and heterotic groups. Only species which were cultured 3 or more times (isolates) were included. Yellow cells indicate the presence of isolate(s). Prevalence refers to the number of maize genotypes that gave rise to at least one cultured isolate from that species. Further details are in Table S1.

Fg -treated			Heterotic Group	European Flint	Early Butler	BSSS	BSSS/ Minnesota 13	Minnesota 13	Minnesota 13	Minnesota 13	P3990
V4 MiSeq OTUs \ Maize Inbred Hybrid Line	Cultured taxa predictions	# of isolates matching V4 MiSeq OTU	Tip/Base	CO444	CO325	CO452	CO462	CO449	CO433	CO432	CO430
OTU2 (MiSeq predicted Enterobacteriaceae family)	*Atlantibacter hermannii* or *Kosakonia cowanii*	15	T	0	0	0	OTU23, OTU33	0	0	OTU18, OTU403	0
			B	OTU30	0	0	0	0	0	0	OTU26
OTU3 (MiSeq predicted Enterobacteriaceae family)	Taxa from the order Enterobacterales*	46	T	OTU91, OTU204, OTU205, OTU202	OTU203	OTU117, OTU118, OTU121, OTU122, OTU124, OTU141, OTU405	OTU74, OTU203, OTU71, OTU204	0	OTU73, OTU117, OTU118, OTU120, OTU146	OTU201	OTU75, OTU118, OTU127
			B	OTU201	0	0	0	OTU202, OTU205	0	0	OTU76, OTU123
OTU4 (MiSeq predicted Enterobacteriaceae family)	*Pantoea agglomerans, Pantoea eucalytpi,* and *Pantoea brenneri*	7	T	0	OTU246, OTU402	0	OTU232, OTU233, OTU238, OTU401	0	0	0	0
			B	0	0	0	0	0	0	0	0
OTU6 (MiSeq predicted Enterobacteriaceae family)	n/a	0	T	0	0	0	0	0	0	0	0
			B	0	0	0	0	0	0	0	0
OTU25 (MiSeq predicted *Pantoea*)	*Pantoea ananatis, Pantoea agglomerans,* or *Pantoea anthophila*	37	T	OTU289, OTU290, OTU291, OTU292	OTU289, OTU290, OTU291, OTU292	OTU268, OTU289, OTU290, OTU291, OTU292	OTU271, OTU289, OTU290, OTU291, OTU292	OTU292, OTU291, OTU267, OTU264, OTU276, OTU265, OTU270	0	0	OTU291, OTU267, OTU264, OTU276, OTU265, OTU270
			B	OTU260, OTU261	0	0	0	OTU289, OTU290, OTU291, OTU292	0	0	OTU265, OTU267, OTU270, OTU276, OTU291
OTU26 (MiSeq predicted *Serratia*)	n/a	0	T	0	0	0	0	0	0	0	0
			B	0	0	0	0	0	0	0	0
OTU28 (MiSeq predicted *Pseudomonas*)	*Pseudomonas parafulva* or *Pseudomonas monteilii*	6	T	OTU317	0	0	0	OTU313, OTU317	OTU314	0	0
			B	OTU317	0	0	0	0	0	0	0
OTU30 (MiSeq predicted *Acinetobacter*)	n/a	0	T	0	0	0	0	0	0	0	0
			B	0	0	0	0	0	0	0	0
OTU31 (MiSeq predicted *Acinetobacter*)	*Acinetobacter oleivorans*	4	T	0	0	0	0	0	0	0	0
			B	0	0	0	0	0	0	0	0
OTU34 (MiSeq predicted *Stenotrophomonas*)	*Stenotrophomonas pavanii* △	40	T	OTU368, OTU370, OTU375	0	OTU367, OTU370, OTU373, OTU374, OTU375	0	0	OTU364, OTU375	OTU375	0
			B	OTU375	0	0	0	OTU367, OTU368, OTU375	0	0	OTU375
OTU35 (MiSeq predicted *Sphingomonas*)	*Sphingomonas yabuuchiae, Sphingomonas*	10	T	OTU363	0	0	0	OTU357	0	OTU358, OTU359, OTU362	OTU358, OTU359, OTU360
			B	0	0	0	0	0	0	0	OTU353

Fg -treated		Heterotic Group		P3990/Iodent	Iodent	Lancaster	Pioneer Hybrid	Pioneer Hybrid	Pioneer Hybrid				
V4 MiSeq OTUs \ Maize Inbred Hybrid Line	Cultured taxa predictions	# of isolates matching V4 MiSeq OTU	Tip/Base	CO448	CO431	CO441	P35837	P38157	P9855HR	Total cultured OTUs	Total cultured OTUs, merged Tip and Base	Prevalence	Prevalance, merged Tip and Base
OTU2 (MiSeq predicted Enterobacteriaceae family)	*Atlantibacter hermannii* or *Kosakonia cowanii*	15	T	OTU17, OTU404	OTU22, OTU24, OTU16, OTU17, OTU29, OTU27, OTU30	0	OTU17	0	OTU21, OTU22, OTU23, OTU24, OTU26, OTU16, OTU17, OTU29, OTU30, OTU27, OTU33	14		6	
			B	0	0	0	0	0	OTU29	3	14	3	8
OTU3 (MiSeq predicted Enterobacteriaceae family)	Taxa from the order Enterobacterales*	46	T	OTU124	0	OTU117, OTU118, OTU122, OTU142, OTU143	OTU80	OTU92, OTU93	OTU201	25		12	
			B	0	0	OTU118, OTU143	0	0	0	7	27	4	13
OTU4 (MiSeq predicted Enterobacteriaceae family)	*Pantoea agglomerans*, *Pantoea eucalyptus*, and *Pantoea brenneri*	7	T	0	OTU230, OTU235	0	0	0	OTU220	9		4	
			B	0	OTU214, OTU230, OTU235	0	0	0	0	3	10	1	4
OTU6 (MiSeq predicted Enterobacteriaceae family)	n/a	0	T	0	0	0	0	0	0	0		0	
			B	0	0	0	0	0	0	0	0	0	0
OTU25 (MiSeq predicted *Pantoea*)	*Pantoea ananatis*, *Pantoea agglomerans*, or *Pantoea anthophila*	37	T	OTU289, OTU290, OTU291, OTU292	OTU270, OTU276, OTU291	OTU268, OTU271, OTU289	OTU270, OTU271, OTU276, OTU289, OTU290, OTU291, OTU292	OTU291, OTU267, OTU264, OTU276, OTU265, OTU270	OTU257, OTU270, OTU271, OTU289, OTU290, OTU291, OTU292	12		12	
			B	0	OTU291, OTU267, OTU265, OTU264, OTU271, OTU276	0	0	0	OTU289, OTU290, OTU291, OTU292	12	14	5	12
OTU26 (MiSeq predicted *Serratia*)	n/a	0	T	0	0	0	0	0	0	0		0	
			B	0	0	0	0	0	0	0	0	0	0
OTU28 (MiSeq predicted *Pseudomonas*)	*Pseudomonas parafulva* or *Pseudomonas monteilii*	6	T	0	0	0	0	OTU317	0	3		4	
			B	0	0	0	0	0	0	1	3	1	4
OTU30 (MiSeq predicted *Acinetobacter*)	n/a	0	T	0	0	0	0	0	0	0		0	
			B	0	0	0	0	0	0	0	0	0	0
OTU31 (MiSeq predicted *Acinetobacter*)	*Acinetobacter oleivorans*	4	T	0	OTU7	0	OTU7, OTU386	0	0	2		2	
			B	0	0	0	0	0	0	0	0	0	0
OTU34 (MiSeq predicted *Stenotrophomonas*)	*Stenotrophomonas pavanii* △	40	T	OTU374, OTU375	OTU374, OTU375	OTU367, OTU374	OTU367, OTU368, OTU370, OTU374, OTU375	OTU367, OTU368, OTU370, OTU374, OTU375	OTU374, OTU375	8		10	
			B	0	OTU374, OTU375	OTU370	OTU375	0	OTU367, OTU368, OTU370, OTU374, OTU375	5	8	7	12
OTU35 (MiSeq predicted *Sphingomonas*)	*Sphingomonas yabuuchiae*, *Sphingomonas*	10	T	0	OTU361	OTU356	0	0	0	8		6	
			B	0	0	0	0	0	0	1	9	1	6

Figure S10. Comparison of V4-MiSeq core OTUs to matching cultured OTUs identified from *Fusarium graminearum*-treated transmitting silks of maize. V4-MiSeq core OTUs from Khalaf et al. [42] were taxa which were determined to be the most dominant and prevalent taxa based on the V4-MiSeq data. Isolates were cultured separately from the tip (T) and base (B) of maize silks spanning diverse host inbred/hybrid lines and heterotic groups. The asterisk (*) indicates that Enterobacterales isolate predictions include *Pantoea agglomerans, Klebsiella aerogenes, Klebsiella variicola, Klebsiella pneumoniae, Enterobacter asburiae, Enterobacter ludwigii, Erwinia persicina, Enterobacter mori,* and *Erwinia aphidicola*. The triangle (△) indicates that 37 of the isolates matching V4-MiSeq OTU 34 were clearly identified as *Stenotrophomonas pavanii*, while 2 isolates had equal first matches to *S. pavanii* and *S. maltophilia*, and a remaining isolate had a lower (96.45%) first match to *S. maltophilia*. Thus, *S. pavanii* is a high confidence taxonomic prediction for V4-MiSeq OTU 34 based on sequence data of 40 cultured isolates. Yellow cells indicate the presence of isolate(s). Red text indicates that no cultured isolate matched the V4-MiSeq OTU. Prevalence refers to the number of maize genotypes that gave rise to at least one cultured isolate from that OTU. Further details are in Table S1.

Healthy

V4 MiSeq OTUs \ Maize Inbred Hybrid Line	Cultured taxa predictions	# of isolates matching V4 MiSeq OTU	Tip/Base	CO444	CO325	CO452	CO462	CO449	CO433	CO432	CO430
		Heterotic Group		European Flint	Early Butler	BSSS	BSSS/ Minnesota 13	Minnesota 13	Minnesota 13	Minnesota 13	P3990
OTU2 (MiSeq predicted Enterobacteriaceae family)	*Atlantibacter hermannii*	8	T	0	0	0	0	OTU25	0	0	0
			B	0	0	OTU24, OTU26	0	0	0	OTU31	0
OTU3 (MiSeq predicted Enterobacteriaceae family)	Taxa from the order Enterobacterales*	18	T	0	OTU119	0	0	OTU92	0	OTU117, OTU118	0
			B	0	0	OTU117	0	OTU117, OTU118	0	OTU71, OTU167	0
OTU4 (MiSeq predicted Enterobacteriaceae family)	*Pantoea ananatis, Pantoea agglomerans,* and *Pantoea brenneri*	18	T	0	0	OTU246, OTU402	0	OTU209, OTU218, OTU246, OTU402	0	0	0
			B	0	0	0	0	0	0	OTU211, OTU217, OTU228, OTU230, OTU232, OTU231, OTU239	OTU225, OTU228, OTU235, OTU230
OTU6 (MiSeq predicted Enterobacteriaceae family)	n/a	0	T	0	0	0	0	0	0	0	0
			B	0	0	0	0	0	0	0	0
OTU25 (MiSeq predicted *Pantoea*)	*Pantoea ananatis, Pantoea agglomerans,* or *Pantoea anthophila*	7	T	0	0	0	0	0	0	OTU270, OTU271, OTU276, OTU290, OTU292	0
			B	0	0	0	0	OTU265, OTU270, OTU271, OTU276	0	OTU292	0
OTU26 (MiSeq predicted *Serratia*)	n/a	0	T	0	0	0	0	0	0	0	0
			B	0	0	0	0	0	0	0	0
OTU28 (MiSeq predicted *Pseudomonas*)	*Pseudomonas cremoricolorata*	1	T	0	0	OTU316	0	0	0	0	0
			B	0	0	0	0	0	0	0	0
OTU30 (MiSeq predicted *Acinetobacter*)	n/a	0	T	0	0	0	0	0	0	0	0
			B	0	0	0	0	0	0	0	0
OTU31 (MiSeq predicted *Acinetobacter*)	*Acinetobacter oleivorans*	2	T	0	0	0	0	0	0	0	0
			B	0	0	0	0	0	0	0	0
OTU34 (MiSeq predicted *Stenotrophomonas*)	*Stenotrophomonas pavanii*	13	T	0	0	OTU375	0	0	0	OTU375	0
			B	0	0	OTU371, OTU374, OTU375	0	0	0	OTU375	0
OTU35 (MiSeq predicted *Sphingomonas*)	*Sphingomonas yabuuchiae*	2	T	0	0	0	0	0	0	0	0
			B	0	0	0	0	0	0	0	0

Healthy		Heterotic Group		P3990/Iodent	Iodent	Lancaster	Pioneer Hybrid	Pioneer Hybrid	Pioneer Hybrid	Total cultured OTUs	Total cultured OTUs, merged Tip and Base	Prevalence	Prevalance, merged Tip and Base	Prevalence from the MiSeq Study (2017 healthy silks) (mean relative abundance)
V4 MiSeq OTUs \ Maize Inbred Hybrid Line	Cultured taxa predictions	# of isolates matching V4 MiSeq OTU	Tip/Base	CO448	CO431	CO441	P35837	P38157	P9855HR					
OTU2 (MiSeq predicted Enterobacteriaceae family)	*Atlantibacter hermannii*	8	T	0	0	0	0	0	OTU16, OTU21	3		2		3.3
			B	0	0	OTU19	0	0	0	4	7	3	5	4.52
OTU3 (MiSeq predicted Enterobacteriaceae family)	Taxa from the order Enterobacterales*	18	T	OTU144, OTU167	0	0	OTU73, OTU203	0	OTU144	8		6		12.19
			B	0	0	0	OTU73, OTU203	0	0	6	9	4	7	15.75
OTU4 (MiSeq predicted Enterobacteriaceae family)	*Pantoea ananatis, Pantoea agglomerans,* and *Pantoea brenneri*	18	T	OTU216	OTU242	0	0	OTU221, OTU222	OTU233, OTU235, OTU238	11		6		4.78
			B	0	OTU246	OTU246, OTU402	0	0	0	11	19	4	9	n/a
OTU6 (MiSeq predicted Enterobacteriaceae family)	n/a	0	T	0	0	0	0	0	0	0		0		5.4
			B	0	0	0	0	0	0	0	0	0	0	3.22
OTU25 (MiSeq predicted *Pantoea*)	*Pantoea ananatis, Pantoea agglomerans,* or *Pantoea anthophila*	7	T	OTU288, OTU289, OTU291, OTU271, OTU290, OTU292	0	0	0	0	0	8		2		15.43
			B	0	OTU258	0	0	0	0	6	10	3	4	25.98
OTU26 (MiSeq predicted *Serratia*)	n/a	0	T	0	0	0	0	0	0	0		0		n/a
			B	0	0	0	0	0	0	0	0	0	0	2.95
OTU28 (MiSeq predicted *Pseudomonas*)	*Pseudomonas cremoricolorata*	1	T	0	0	0	0	0	0	1		1		n/a
			B	0	0	0	0	0	0	0	1	0	1	1.39
OTU30 (MiSeq predicted *Acinetobacter*)	n/a	0	T	0	0	0	0	0	0	0		0		n/a
			B	0	0	0	0	0	0	0	0	0	0	n/a
OTU31 (MiSeq predicted *Acinetobacter*)	*Acinetobacter oleivorans*	2	T	0	OTU8	OTU7	0	0	0	2		2		1.78
			B	0	0	0	0	0	0	0	2	0	2	n/a
OTU34 (MiSeq predicted *Stenotrophomonas*)	*Stenotrophomonas pavanii*	13	T	OTU375	0	OTU375	OTU369	0	OTU374	3		6		2.99
			B	0	0	0	0	0	0	3	4	2	6	1.42
OTU35 (MiSeq predicted *Sphingomonas*)	*Sphingomonas yabuuchiae*	2	T	0	0	OTU357	0	0	0	1		1		n/a
			B	0	0	OTU357	0	0	0	1	1	1	1	1.05

Figure S11. Comparison of V4-MiSeq core OTUs to matching cultured OTUs identified from healthy transmitting silks of maize. V4-MiSeq core OTUs from Khalaf et al. [42] were taxa which were determined to be the most dominant and prevalent taxa, based on the V4-MiSeq data. Isolates were cultured separately from the tip (T) and base (B) of maize silks spanning diverse host inbred/hybrid lines and heterotic groups. The asterisk (*) indicates that Enterobacterales isolate predictions include *Enterobacter asburiae, Klebsiella aerogenes, Klebsiella variicola, Leclercia adecarboxylata, Enterobacter ludwigii,* and *Erwinia aphidicola*. Yellow cells indicate the presence of isolate(s). Red text indicates that no cultured isolate matched the V4-MiSeq OTU. Orange text indicates OTUs which were not present when healthy silks were analysed alone, but were added when analysed alongside *F. graminearum*-treated silk samples because the isolates matched multiple longer or higher quality 16S sequences from the *F. graminearum*-treated population that were revealed to be distinct. Prevalence refers to the number of maize genotypes that gave rise to at least one cultured isolate from that OTU. Further details are in Table S1.

Healthy			Heterotic Group		European Flint	Early Butler	BSSS	BSSS/ Minnesota 13	Minnesota 13	Minnesota 13	Minnesota 13	P3990	P3990/Iodent
V4-MiSeq *Fg* -induced taxa and core taxa grouping	Maize Inbred Hybrid Line / V4 MiSeq OTUs	Cultured taxa predictions	# of isolates matching V4 MiSeq OTU	Tip/Base	CO444	CO325	CO452	CO462	CO449	CO433	CO432	CO430	CO448
Core & Consistent indicator	OTU2 (MiSeq predicted Enterobacteriaceae family)	*Atlantibacter hermannii*	8	T	0	0	0	0	OTU25	0	0	0	0
				B	0	0	OTU24, OTU26	0	0	0	OTU31	0	0
Core & Consistent indicator	OTU3 (MiSeq predicted Enterobacteriaceae family)	Taxa from the order Enterobacterales*	18	T	0	OTU119	0	0	OTU92	0	OTU117, OTU118	0	OTU144, OTU167
				B	0	0	OTU117	0	OTU117, OTU118	0	OTU71, OTU167	0	0
Core & Consistent indicator	OTU4 (MiSeq predicted Enterobacteriaceae family)	*Pantoea ananatis, Pantoea agglomerans,* and *Pantoea brenneri*	18	T	0	0	OTU246, OTU402	0	OTU209, OTU218,	0	0	0	OTU216
				B	0	0	0	0	0	0	OTU211, OTU217, OTU228, OTU230, OTU232, OTU231, OTU239	OTU225, OTU228, OTU235, OTU230	0
Core & Consistent indicator	OTU34 (MiSeq predicted *Stenotrophomonas*)	*Stenotrophomonas pavanii*	13	T	0	0	OTU375	0	0	0	OTU375	0	OTU375
				B	0	0	OTU371, OTU374, OTU375	0	0	0	OTU375	0	0
Core & Consistent indicator	OTU35 (MiSeq predicted *Sphingomonas*)	*Sphingomonas yabuuchiae*	2	T	0	0	0	0	0	0	0	0	0
				B	0	0	0	0	0	0	0	0	0
Core & Base 2017 indicator	OTU6 (MiSeq predicted Enterobacteriaceae family)	n/a	0	T	0	0	0	0	0	0	0	0	0
				B	0	0	0	0	0	0	0	0	0
Core & Base 2017 indicator	OTU25 (MiSeq predicted *Pantoea*)	*Pantoea ananatis, Pantoea agglomerans,* or *Pantoea anthophila*	7	T	0	0	0	0	0	0	OTU270, OTU271, OTU276, OTU290, OTU292	0	OTU288, OTU289, OTU291, OTU271, OTU290, OTU292
				B	0	0	0	0	OTU265, OTU270, OTU271, OTU276	0	OTU292	0	0
Consistent indicator	OTU7 (MiSeq predicted *Herbaspirillum*)	n/a	0	T	0	0	0	0	0	0	0	0	0
				B	0	0	0	0	0	0	0	0	0
Consistent indicator	OTU8 (MiSeq predicted *Delftia*)	n/a	0	T	0	0	0	0	0	0	0	0	0
				B	0	0	0	0	0	0	0	0	0
Tip 2017 indicator	OTU5 (MiSeq predicted Enterobacteriaceae family)	n/a	0	T	0	0	0	0	0	0	0	0	0
				B	0	0	0	0	0	0	0	0	0
Tip 2017 indicator	OTU39 (MiSeq predicted *Luteibacter*)	n/a	0	T	0	0	0	0	0	0	0	0	0
				B	0	0	0	0	0	0	0	0	0
Tip 2017 indicator	OTU9 (MiSeq predicted Burkholderiaceae family)	n/a	0	T	0	0	0	0	0	0	0	0	0
				B	0	0	0	0	0	0	0	0	0
Tip 2017 indicator	OTU49 (MiSeq predicted *Rhizobium*)	*Rhizobium nepotum*	1	T	0	0	0	0	0	0	0	0	0
				B	0	0	0	0	0	0	0	0	0
Tip 2017 indicator	OTU45 (MiSeq predicted *Sphingobacterium*)	n/a	0	T	0	0	0	0	0	0	0	0	0
				B	0	0	0	0	0	0	0	0	0
Tip 2017 indicator	OTU32 (MiSeq predicted *Acinetobacter*)	n/a	0	T	0	0	0	0	0	0	0	0	0
				B	0	0	0	0	0	0	0	0	0
Tip 2017 indicator	OTU50(MiSeq predicted *Pseudomonas*)	n/a	0	T	0	0	0	0	0	0	0	0	0
				B	0	0	0	0	0	0	0	0	0
Base 2017 indicator	OTU17(MiSeq predicted Enterobacteriaceae family)	*Klebsiella grimontii* or *Klebsiella oxytoca*	5	T	0	0	0	0	0	0	0	0	0
				B	0	0	0	0	0	0	OTU140	0	0

Healthy

V4-MiSeq *Fg*-induced taxa and core taxa grouping	V4 MiSeq OTUs / Maize Inbred Hybrid Line	Cultured taxa predictions	# of isolates matching V4 MiSeq OTU	Tip/Base	Iodent CO431	Lancaster CO441	Pioneer Hybrid P35837	Pioneer Hybrid P38157	Pioneer Hybrid P9855HR	Total cultured OTUs	Total cultured OTUs, merged Tip and Base	Prevalence	Prevalance, merged Tip and Base
Core & Consistent indicator	OTU2 (MiSeq predicted Enterobacteriaceae family)	*Atlantibacter hermannii*	8	T	0	0	0	0	OTU16, OTU21	3		2	
				B	0	OTU19	0	0	0	4	7	3	5
Core & Consistent indicator	OTU3 (MiSeq predicted Enterobacteriaceae family)	Taxa from the order Enterobacterales*	18	T	0	0	OTU73, OTU203	0	OTU144	8		6	
				B	0	0	OTU73, OTU203	0	0	6	9	4	7
Core & Consistent indicator	OTU4 (MiSeq predicted Enterobacteriaceae family)	*Pantoea ananatis, Pantoea agglomerans,* and *Pantoea brenneri*	18	T	OTU242	0	0	OTU221, OTU222	OTU233, OTU235, OTU238	11		6	
				B	OTU246	OTU246, OTU402	0	0	0	11	19	4	9
Core & Consistent indicator	OTU34 (MiSeq predicted *Stenotrophomonas*)	*Stenotrophomonas pavanii*	13	T	0	OTU375	OTU369	0	OTU374	3		6	
				B	0	0	0	0	0	3	4	2	6
Core & Consistent indicator	OTU35 (MiSeq predicted *Sphingomonas*)	*Sphingomonas yabuuchiae*	2	T	0	OTU357	0	0	0	1		1	
				B	0	OTU357	0	0	0	1	1	1	1
Core & Base 2017 indicator	OTU6 (MiSeq predicted Enterobacteriaceae family)	n/a	0	T	0	0	0	0	0	0		0	
				B	0	0	0	0	0	0	0	0	0
Core & Base 2017 indicator	OTU25 (MiSeq predicted *Pantoea*)	*Pantoea ananatis, Pantoea agglomerans,* or *Pantoea anthophila*	7	T	0	0	0	0	0	8		2	
				B	OTU258	0	0	0	0	6	10	3	4
Consistent indicator	OTU7 (MiSeq predicted *Herbaspirillum*)	n/a	0	T	0	0	0	0	0	0		0	
				B	0	0	0	0	0	0	0	0	0
Consistent indicator	OTU8 (MiSeq predicted *Delftia*)	n/a	0	T	0	0	0	0	0	0		0	
				B	0	0	0	0	0	0	0	0	0
Tip 2017 indicator	OTU5 (MiSeq predicted Enterobacteriaceae family)	n/a	0	T	0	0	0	0	0	0		0	
				B	0	0	0	0	0	0	0	0	0
Tip 2017 indicator	OTU39 (MiSeq predicted *Luteibacter*)	n/a	0	T	0	0	0	0	0	0		0	
				B	0	0	0	0	0	0	0	0	0
Tip 2017 indicator	OTU9 (MiSeq predicted Burkholderiaceae family)	n/a	0	T	0	0	0	0	0	0		0	
				B	0	0	0	0	0	0	0	0	0
Tip 2017 indicator	OTU49 (MiSeq predicted *Rhizobium*)	*Rhizobium nepotum*	1	T	0	0	0	OTU331	0	1		1	
				B	0	0	0	0	0	0	1	0	1
Tip 2017 indicator	OTU45 (MiSeq predicted *Sphingobacterium*)	n/a	0	T	0	0	0	0	0	0		0	
				B	0	0	0	0	0	0	0	0	0
Tip 2017 indicator	OTU32 (MiSeq predicted *Acinetobacter*)	n/a	0	T	0	0	0	0	0	0		0	
				B	0	0	0	0	0	0	0	0	0
Tip 2017 indicator	OTU50(MiSeq predicted *Pseudomonas*)	n/a	0	T	0	0	0	0	0	0		0	
				B	0	0	0	0	0	0	0	0	0
Base 2017 indicator	OTU17(MiSeq predicted Enterobacteriaceae family)	*Klebsiella grimontii* or *Klebsiella oxytoca*	5	T	OTU130	0	0	OTU130, OTU138	OTU130, OTU138	1		3	
				B	0	0	0	0	0	1	2	1	4

Figure S12| Comparison of *Fusarium graminearum*-induced V4-MiSeq OTUs (*F. graminearum*-indicators) to matching cultured OTUs isolated from untreated (healthy) transmitting silks of maize. Isolates were cultured separately from the tip (T) and base (B) of maize silks spanning diverse host inbred/hybrid lines and heterotic groups. V4-MiSeq *F. graminearum*-induced OTUs (noted as *F. graminearum* indicators in extreme left column) from Khalaf et al. [42] were taxa which had elevated abundance in transmitting silks treated with *F. graminearum* compared to untreated silks (healthy silks); it is indicated whether these taxa were elevated in 2017 silk tip or base tissues exclusively, or consistent indicators across tip tissues in both 2016 and 2017. Some *F. graminearum*-induced indicator taxa were also members of the predicted core transmitting silk microbiome from Khalaf et al. [42] (white text in extreme left column). The asterisk (*) indicates that Enterobacterales isolate predictions include *Enterobacter asburiae, Klebsiella aerogenes, Klebsiella variicola, Leclercia adecarboxylata, Enterobacter ludwigii,* and *Erwinia aphidicola*. Yellow cells indicate the presence of isolate(s). Red text indicates that no cultured isolate matched the V4-MiSeq OTU. Orange text indicates OTUs which were not present when healthy silks were analysed alone, but were added when analysed alongside *F. graminearum*-treated silk samples because the isolates matched multiple longer or higher quality 16S sequences from the *F. graminearum*-treated population that were revealed to be distinct. Prevalence refers to the number of maize genotypes that gave rise to at least one cultured isolate from that OTU. Further details are in Table S1.
